# Precise targeting of human HIV broadly neutralizing antibody precursors

**DOI:** 10.1126/science.adv5572

**Published:** 2025-07-31

**Authors:** Tom G. Caniels, Madhu Prabhakaran, Gabriel Ozorowski, Kellie J. MacPhee, Weiwei Wu, Karlijn van der Straten, Sashank Agrawal, Ronald Derking, Emma I.M.M. Reiss, Katrina Millard, Martina Turroja, Aimee Desrosiers, Jeffrey Bethony, Elissa Malkin, Marinus H. Liesdek, Annelou van der Veen, Michelle Klouwens, Jonne L. Snitselaar, Joey H. Bouhuijs, Rhianna Bronson, Jalen Jean-Baptiste, Suprabhath Gajjala, Zahra Rikhtegaran Tehrani, Alison Benner, Mukundhan Ramaswami, Michael O. Duff, Yung-Wen Liu, Alicia H. Sato, Ju Yeong Kim, Isabel J.L. Baken, Catarina Mendes Silva, Tom P. L. Bijl, Jacqueline van Rijswijk, Judith A. Burger, Albert Cupo, Anila Yasmeen, Swastik Phulera, Wen-Hsin Lee, Kipchoge N. Randall, Shiyu Zhang, Martin M. Corcoran, Isabel Regadas, Alex C. Sullivan, David M. Brown, Jennifer A. Bohl, Kelli M. Greene, Hongmei Gao, Nicole L. Yates, Sheetal Sawant, Jan M. Prins, Neeltje A. Kootstra, Stephen M. Kaminsky, Burc Barin, Farhad Rahaman, Margaret Meller, Vince Philiponis, Dagna S. Laufer, Angela Lombardo, Lindsey Mwoga, Solmaz Shotorbani, Drienna Holman, Richard A. Koup, Per Johan Klasse, Gunilla B. Karlsson Hedestam, Georgia D. Tomaras, Marit J. van Gils, David C. Montefiori, Adrian B. McDermott, Ollivier Hyrien, John P. Moore, Ian A. Wilson, Andrew B. Ward, David J. Diemert, Godelieve J. de Bree, Sarah F. Andrews, Marina Caskey, Rogier W. Sanders

**Affiliations:** 1 Department of Medical Microbiology and Infection Prevention, Amsterdam UMC, location AMC; Amsterdam, the Netherlands.; 2 Amsterdam institute for Immunology and Infectious diseases; Amsterdam, the Netherlands.; 3 Vaccine Research Center, National Institute of Allergy and Infectious Diseases, National Institutes of Health; Bethesda, MD, USA.; 4 Department of Integrative Structural and Computational Biology, The Scripps Research Institute; La Jolla, CA, USA.; 5 Vaccine and Infectious Disease Division, Fred Hutchinson Cancer Center; Seattle, WA, USA.; 6 Laboratory of Molecular Immunology, The Rockefeller University; New York, NY, USA.; 7 Vaccine Research Unit, The George Washington University; Washington, DC, USA.; 8 Department of Internal Medicine, Amsterdam UMC, location AMC; Amsterdam, the Netherlands.; 9 Department of Microbiology and Immunology, Weill Cornell Medicine; New York, NY, USA.; 10 Department of Microbiology, Tumor and Cell Biology, Karolinska Institutet; Stockholm, Sweden.; 11 Foundation for the National Institutes of Health, Inc.; Bethesda, MD, USA.; 12 Department of Surgery, Duke University Medical Center; Durham, NC, USA.; 13 Department of Experimental Immunology, Amsterdam UMC, location AMC; Amsterdam, the Netherlands.; 14 Department of Genetic Medicine, Weill Cornell Medicine; New York, NY, USA.; 15 The Emmes Company, Rockville, MD, USA.; 16 International AIDS Vaccine Initiative (IAVI); New York, NY, USA.; 17 The Skaggs Institute for Chemical Biology, The Scripps Research Institute; La Jolla, CA, USA.

## Abstract

A protective HIV vaccine will need to induce broadly neutralizing antibodies (bnAbs) in humans, but priming rare bnAb precursor B cells has been challenging. In a double-blinded, placebo-controlled phase 1 human clinical trial, the recombinant, germline-targeting envelope glycoprotein (Env) trimer BG505 SOSIP.v4.1-GT1.1, adjuvanted with AS01_B_, induced bnAb precursors of the VRC01-class at a high frequency in the majority of vaccine recipients. These bnAb precursors, that target the CD4 receptor binding site, had undergone somatic hypermutation characteristic of the VRC01-class. A subset of isolated VRC01-class monoclonal antibodies neutralized wild-type pseudoviruses and was structurally extremely similar to bnAb VRC01. These results further support germline-targeting approaches for human HIV vaccine design and demonstrate atomic-level manipulation of B cell responses with rational vaccine design.

Despite decades of research, an HIV vaccine remains elusive. A major hurdle is the extensive viral sequence diversity of HIV, in particular in its envelope glycoprotein (Env) trimer, the target for neutralizing antibodies (nAbs). Substantial efforts have focused on the induction of broadly neutralizing antibodies (bnAbs) that can cope with this diversity (1–3). bnAbs have been isolated from people with HIV (PWH) and can protect against virus challenge in non-human primates (NHPs) (4–7). Furthermore, the AMP trials showed that bnAb VRC01, which targets the conserved CD4 receptor binding site (CD4bs) on HIV Env, could prevent infection by sensitive HIV isolates when present at high concentrations (8, 9).

During infection, HIV bnAbs can arise in a coevolutionary process between virus and the host immune system. As the virus escapes from narrow specificity nAbs by mutating the epitope(s), renewed rounds of B cell affinity maturation triggered by the mutated epitope(s) can drive the emergence of bnAbs that can neutralize diverse viruses (10–12). However, most B cells do not have the intrinsic genetic capacity to become bnAb-producing B cells, and those B cells that do have the desired properties are generally not activated by recombinant Env immunogens (13, 14). These observations have prompted the design of immunogens that can activate low-affinity and rare bnAb precursor B cells. This ‘germline-targeting’ (GT) approach aims to expand such germline bnAb precursors through priming with a GT priming immunogen, followed by shaping and polishing boosts with increasingly native Env immunogens to guide a bnAb maturation pathway (3).

VRC01-class bnAbs in particular are attractive for GT vaccination strategies, because they have been isolated from multiple PWH, suggesting that they can be generated reproducibly in humans, and because they include the broadest and most potent HIV bnAbs described to date (15–19). Moreover, VRC01-class bnAbs have very distinct and targetable genetic and structural characteristics. Specifically, they have restricted immunoglobulin heavy chain variable region alleles (IGHV1–2*02 or *04) and a short five amino acid light chain complementarity determining region 3 (CDRL3). VRC01-class bnAbs are generally highly mutated and can accommodate the most prominent glycan shielding the CD4bs, at position 276 (N276 glycan). However, VRC01-class bnAb precursors are extremely rare, with frequency estimates of 1 in 200,000 to 1 in 1,200,000 B cells in the naive repertoire (20, 21). One priming immunogen designed to bind to VRC01-class B cell precursors is the eOD-GT8–60mer, which consists of a modified and multimerized gp120 fragment. A first-in-human clinical trial (IAVI G001) revealed that eOD-GT8–60mer has the ability to activate germline VRC01-class B cells in humans providing proof-of-concept for the first (priming) step of the GT strategy (14, 21–23).

We hypothesized that a native-like Env trimer offers advantages over other GT platforms because it allows for presentation of multiple bnAb epitopes while imposing structural constraints similar to those of the functional, virus-associated Env trimer. Accordingly, based on the prototypic BG505 SOSIP trimer, we designed GT1.1 to engage bnAb precursors against the trimer apex and the CD4bs, specifically those of the VRC01-class (24–27). GT1.1 was able to activate diverse VRC01-class bnAb precursor B cells in knock-in mouse models and CD4bs-directed B cells in macaques (24). Moreover, GT1.1 priming, followed by boosting with fully glycosylated Env trimers in both infant and adult macaques elicited a CD4bs-directed humoral response that included nAbs with some breadth (24, 25).

## GT1.1 in combination with AS01_B_ has an acceptable safety and reactogenicity profile

**Fig. 1.**
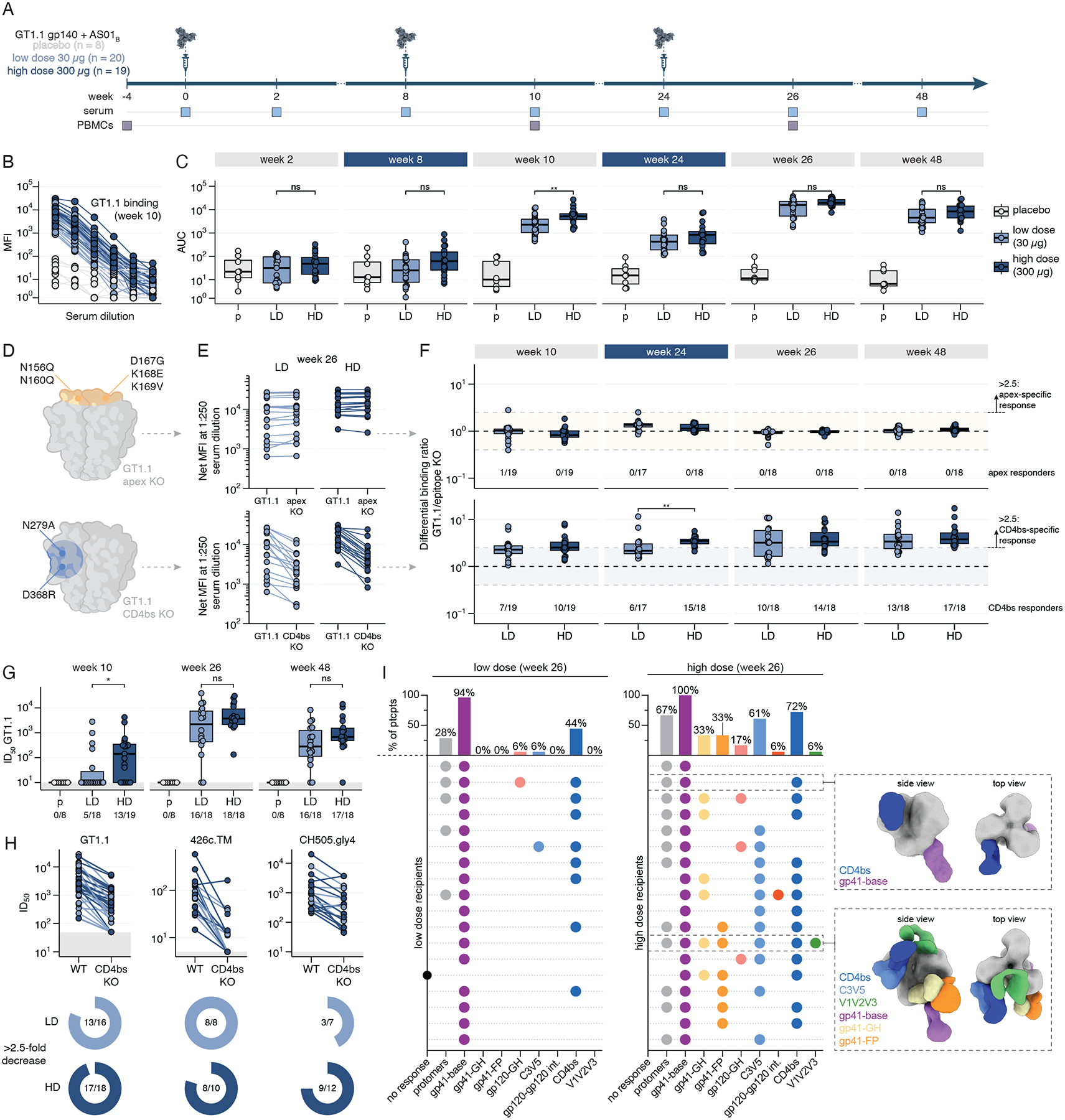
Immunogenicity of GT1.1 in adults without HIV. All statistical tests: Wilcoxon rank-sum test: ns, not significant (p > 0.05); *, p < 0.05; **, p < 0.01; ****, p < 0.0001. (**A**) Overview of vaccination schedule and sample collection. (**B**) Example of BAMA data that were used to generate area under the curve (AUC) calculations. (**C**) GT1.1 binding AUC for placebo (p), low dose (LD) and high dose (HD) recipients at the indicated time points. (**D**) Design of knockout (KO) proteins to measure epitope-specific responses towards the apex and CD4-binding site (CD4bs). The colored region schematically represents the epitope that is knocked out; the mutations are changes from GT1.1 to knock out respective epitopes. (**E**) Example of binding (in background-removed, i.e., net, MFI) to GT1.1, GT1.1 apex KO and GT1.1 CD4bs KO proteins, which were used to calculate the binding ratios. (**F**) Differential binding ratios (GT1.1/GT1.1 apex KO, top panel; GT1.1/GT1.1 CD4bs KO, bottom panel) calculated based on net MFI values (see Methods). A ratio >2.5 (shaded area) was considered a significantly positive CD4bs-specific response. The numbers in the graphs represent the number of participants with a ratio >2.5 at each time point. (**G**) Serum neutralizing antibody (nAb) titers (50% inhibitory dilutions; ID50) at week 26 to GT1.1 pseudovirus. The numbers on the x-axis indicate the participants with a positive titer (ID50 > 10). (**H**) Serum nAb titers (ID50) to GT1.1, 426c.TM and CH505.gly4 viruses and their CD4bs-KO mutants that were designed to reduce or eliminate neutralization mediated by CD4bs-directed antibodies. The pie charts summarize the number of participants in each dose group that have a >2.5-fold decrease in nAb activity against the CD4bs-KO mutant viruses. (**I**) Electron microscopy-based polyclonal epitope mapping (EMPEM) analysis of week 26 samples from low and high dose recipients. Each horizontal line depicts one low dose recipient (left) or high dose recipient (right). The bars represent the frequency of participants with a certain polyclonal epitope response. The 3D reconstructions on the right show epitopes targeted in representative participants.

The IAVI C101 study (www.ClinicalTrials.gov, NCT04224701), was a first-in-human, multicenter, randomized, double-blinded, placebo-controlled, dose-escalation phase 1 trial to evaluate the safety and immunogenicity of the BG505 SOSIP GT1.1 gp140 (GT1.1) vaccine (28) adjuvanted with AS01_B_, in healthy adults without HIV infection. 47 participants were enrolled in the study from October 2020 to January 2022 at three sites and randomized to receive 30 μg (low dose, n = 20) or 300 μg (high dose, n = 19) of GT1.1 with 50 μg of AS01_B_, or placebo (n = 8) administered at weeks 0, 8 and 24 (Fig. 1A, fig. S1, tables S1–S5). 44 participants (94%) received all scheduled vaccinations (fig. S1). No serious adverse events (SAEs) or potential immune mediated disorders considered related to vaccination were reported (table S2). 95% of vaccine recipients reported at least one adverse event (AE; fig. S2 tables S3–S4, Supplementary Text). Reactogenicity events were similar between dose groups and vaccinations, and the majority were mild (grade 1) or moderate (grade 2) severity (72%; table S2). Four vaccine recipients (10%) reported grade 3 local reactogenicities that improved or were fully resolved within three days. No skin abnormalities at the injection site were reported other than transient grade 1 or 2 erythema or one grade 2 edema that resolved within eight days (table S3). Four participants developed vaccine-induced seropositivity. There was no HIV acquisition during the study. Overall, the vaccine had an acceptable safety and tolerability profile.

## GT1.1 induces CD4bs-specific antibody responses

All vaccine recipients (38/38) generated serum IgG antibodies to GT1.1 after two vaccinations, as detected by a binding antibody multiplex assay (BAMA) (Fig. 1B–C, figs. S3–S5). Vaccine-induced IgG responses peaked after the third vaccination and all vaccine recipients displaying serum IgG binding titers for at least six months thereafter (Fig. 1C). We did not detect apex-specific IgG antibodies in serum (Fig. 1D–F), but the majority of participants developed detectable serum antibodies against the CD4bs after three GT1.1 vaccinations (low dose: 10/18 (56%); high dose: 14/18 (78%), Fig. 1F). Similarly, the majority of vaccine recipients generated a response directed towards the trimer base as measured by differential binding to a BG505 SOSIP base knockout (KO) trimer (fig. S5B).

Three GT1.1 vaccinations induced nAbs against the engineered autologous GT1.1 virus in 16/18 (89%) and 18/18 (100%) of low and high dose recipients, respectively (Fig. 1G). In all but one participant across dose groups, GT1.1 neutralization persisted until at least six months after the third vaccination (Fig. 1G). In 10/15 (67%) and 16/18 (89%) of low and high dose participants, respectively, GT1.1 neutralization was at least in part mediated by CD4bs-specific serum antibodies, as the neutralization was reduced when a KO mutation (N279K) was present in the CD4bs (Fig. 1H). A subset of sera neutralized heterologous early CD4bs bnAb precursor viruses 426c.TM and CH505TF.gly4 that were designed to measure VRC01-class and CH103-class precursor bnAbs, respectively (Fig. 1H). Electron microscopy-based polyclonal epitope mapping (EMPEM) confirmed that CD4bs-specific serum antibodies were one of the dominant serum specificities in both low and high dose recipients after three vaccinations. Thus, 8/18 (44%) and 13/18 (72%) of low and high dose recipients had this dominant antibody specificity, respectively (Fig. 1I). We conclude that GT1.1 was highly immunogenic and induced neutralizing antibody responses that target the CD4bs.

## GT1.1 induces memory B cells against the CD4bs and trimer apex

**Fig. 2.**
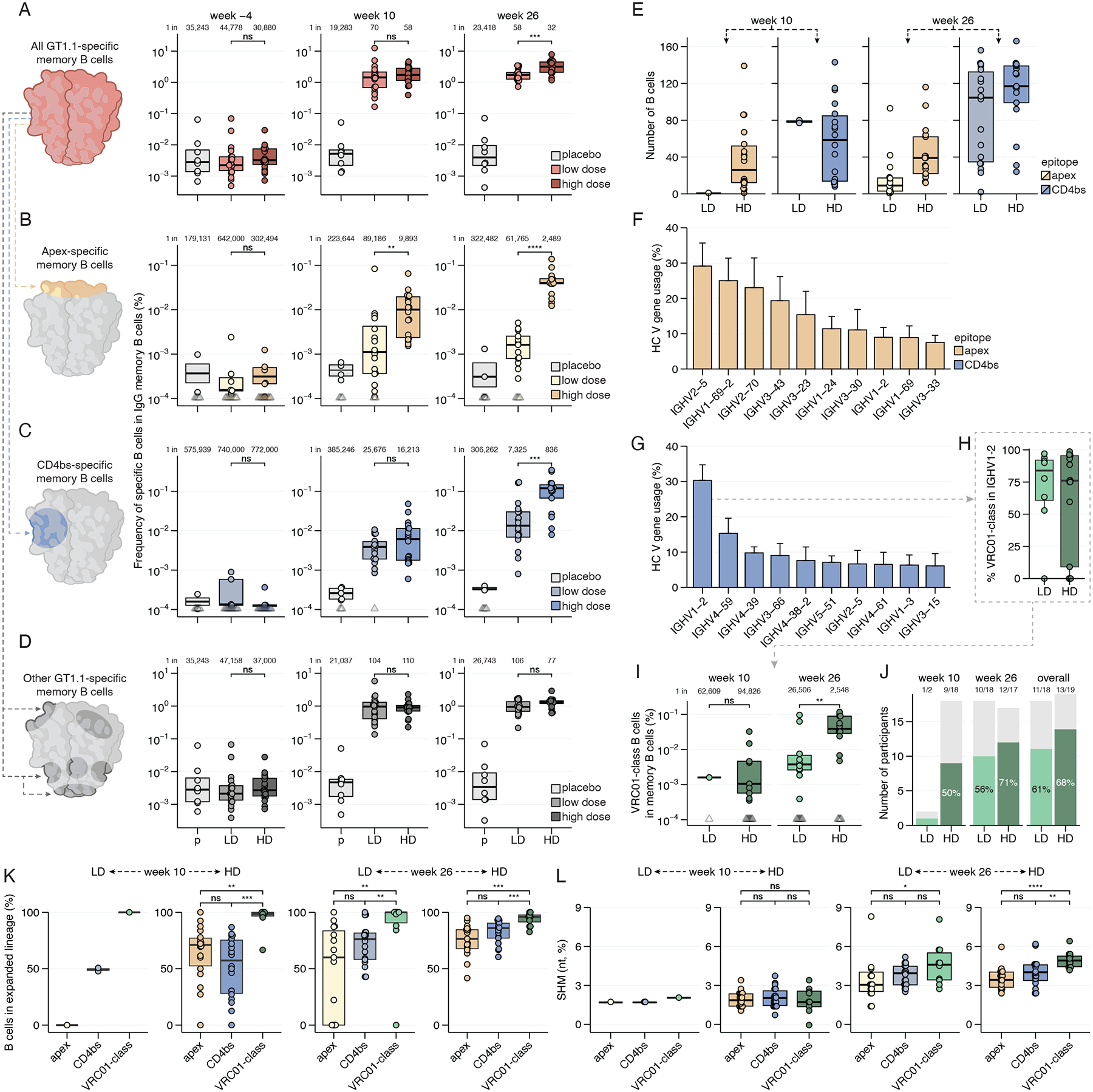
Induction of epitope-specific IgG memory B cells after GT1.1 vaccination. Each dot represents one trial participant in all panels; all statistical tests: Wilcoxon rank-sum test: ns, not significant (p > 0.05); *, p < 0.05; **, p < 0.01; ****, p < 0.0001. **(A-D)** Frequency of GT1.1-specific IgG memory B cells (**A**), apex-specific IgG memory B cells (**B**), CD4bs-specific IgG memory B cells (**C**) and non-apex, non-CD4bs but GT1.1-specific IgG memory B cells (“other specificities”, (**D**)) present in peripheral blood mononuclear cells (PBMCs) from the placebo (p), low dose (LD) and high dose (HD) groups. The numbers on top of the graphs represent the median frequency of the indicated population in the total IgG memory B cell compartment. Wilcoxon rank-sum test: ns, not significant (p > 0.05); ***, p < 0.001; ****, p < 0.0001. **(E)** Number of apex-specific (yellow/orange) and CD4bs-specific (blue) B cell receptor (BCR) sequences obtained from high dose recipients at weeks 10 and 26. **(F)** Mean ± SEM immunoglobulin heavy chain variable gene (IGHV) usage for apex-specific B cells in both groups and timepoints combined. Frequency of gene usage was determined for each vaccine recipient; the bars represent the mean gene usage across individuals. IGHV genes were included when present in at least 10 vaccine recipients, and only the top 10 expressed IGHV genes are shown. (**G**) Mean ± SEM immunoglobulin heavy chain variable gene (IGHV) usage for CD4bs-specific B cells in both groups and timepoints combined as in (**F**). (**H**) Frequency of VRC01-class B cells, defined as a BCR signature of IGHV1–2 with a five residue CDRL3, in CD4bs-specific B cells that use IGHV1–2. **(I)** Frequency of VRC01-class memory B cells in total memory B cells. The numbers on top of the graph represent the median frequency of the VRC01-class B cells in the total IgG memory B cell compartment. Boxplots were constructed using positive VRC01-class responders. **(J)** Number of participants with a VRC01-class response, defined as having detected ≥1 VRC01-class IgG memory B cell. **(K)** Frequency of B cells in an expanded lineage. Expanded lineages are defined as consisting of ≥2 members. **(L)** Percent somatic hypermutation (SHM) expressed as a frequency of nucleotides mutated compared to germline sequences.

GT1.1 was designed to elicit apex- and CD4bs-specific bnAb precursor B cells (24, 26). To enable analysis of these specificities, we used apex and CD4bs epitope KO versions of GT1.1, as well as a “super knockout” antigen that combined both sets of KO mutations (Methods, Fig. 1D). This approach enabled the classification of antigen-specific B cells into three categories: CD4bs-specific, apex-specific, and other specificities (fig. S8). We analyzed peripheral blood mononuclear cell (PBMC) samples from all trial participants at three time points: pre-vaccination (week −4), two weeks after the second vaccination (week 10) and two weeks following the third vaccination (week 26) (Fig. 1A and fig. 2, figs. S6–12, tables S6–S10).

All vaccine recipients generated GT1.1-specific memory B cells after two vaccinations, with median frequencies of 1.4% and 1.7% (range 0.2–12%) of all memory B cells in low and high dose recipients, respectively (Fig. 2A, figs. S9–11). After the third vaccination, high dose recipients had significantly higher frequencies of GT1.1-specific memory B cells than low dose recipients (3.2% *versus* 1.7%, respectively, *p* = 0.00095, fig. 2A). All high dose recipients generated apex-specific memory B cells at a high frequency (~1 in 10,000 and ~1 in 2,500 of memory B cells after the second and third vaccinations, respectively). In low dose recipients, the corresponding frequencies were 9-fold lower at week 10 and 25-fold lower at week 26 (Fig. 2B). After the second vaccination, all but one low dose recipient and all high dose recipients generated CD4bs-specific memory B cells (Fig. 2C). The third vaccination increased CD4bs-specific memory B cell frequencies 3.7-fold for low dose recipients and 19-fold for high dose recipients (Fig. 2C). Notably, at week 26, ~1 in 800 of all memory B cells in high dose recipients targeted the CD4bs, 9-fold higher than in the low dose group (p = 0.00083, fig. 2C). The majority of GT1.1-specific B cells were classified as targeting other epitopes and were thus not directed to either the apex or CD4bs (Fig. 2D). That finding is consistent with observations on serum antibody specificities (Fig. 1I) and reinforces the notion that bnAb epitopes are subdominant compared to, for example, the trimer base or BG505 strain-specific glycan holes (24, 29, 30). Nevertheless, we conclude that GT1.1 recipients, particularly those receiving a high dose, consistently developed CD4bs and apex-specific IgG memory B cells at high frequencies.

## GT1.1 primes VRC01-class precursor B cells

To investigate whether GT1.1-induced CD4bs-specific and apex-specific B cells were genetically similar to bnAbs, we sequenced the B cell receptors (BCRs) of memory B cells from the high dose group at weeks 10 and 26 and from the low dose group at weeks −4 (pre-vaccination) and 26 (fig. S6, tables S). Two low dose recipients had low cell numbers at week 26; therefore, their week 10 BCRs were also sequenced. We also genotyped trial participants for their IGHV allele content using IgDiscover to allow precise allele assignments and optimal BCR sequence analyses (table S11) (31). In total, we obtained 1,706 apex-specific paired BCR sequences and 4,633 CD4bs-specific paired BCR sequences from memory B cells (Fig. 2E, fig. S13). Among all vaccine recipients, the predominant IGHV genes used by apex-specific B cells were IGHV2–5 (29%) and IGHV1-69-2 (25%) while IGHV3 genes, found in multiple apex bnAbs (3, 32–34), were also observed (Fig. 2F, fig. S14). Although long CDRH3s were present in apex-specific B cells, there was no apparent enrichment for them (fig. S15). In contrast, CD4bs-specific BCRs had high frequencies of IGHV1–2 (30%), IGHV4–59 (15%) and IGHV4–39 (10%; Fig. 2G, fig. S14). Notably, >75% of IGHV1–2 memory B cells in both low and high dose recipients were paired with a short five amino acid CDRL3 typical of VRC01-class bnAbs (Fig. 2H), and were thus categorized as VRC01-class. VRC01-class B cells made up 4–74% of the CD4bs-specific memory B cell repertoire, with a median of 17% and 33% for low and high dose recipients, respectively (fig. S16). The frequency of VRC01-class memory B cells in high dose vaccine recipients was significantly higher (~10-fold) in high dose vaccine recipients than in low dose recipients, with medians of ~1 in 2,500 for high dose *versus* ~1 in 26,000 for low dose recipients (*p* = 0.009, fig. 2I). The overall proportion of vaccine recipients with a VRC01-class response was similar between dose groups (LD: 11/18 (61%); HD: 13/19 (68%)) (Fig. 2J, fig. S16). In pre-vaccination time points from 21 trial participants, we were unable to detect any GT1.1-specific VRC01-class B cells in the naive IgD or memory IgG B cell compartment, despite sampling 300 million PBMCs per participant (fig. S12C), indicating that GT1.1 vaccination caused a considerable enrichment of VRC01-class B cells that increased after the third vaccination.

## VRC01-class responses depend on IGHV1–2 alleles

In addition to the requirement of expressing IGHV1–2, VRC01-class bnAbs are further restricted in their allele usage. IGHV1–2 has six known alleles, of which three are known to be compatible with VRC01-class recognition of the CD4bs: *02, *02 with a synonymous polymorphism *02_S4953, and *04 (35). Using individualized germline databases (31), we identified five alleles in our study (*02, *02_S4953, *04, *05 and *06) that are used by GT1.1-specific B cells at different frequencies (fig. S17, table S11). IGHV1–2*02 was the dominant IGHV1–2 allele, followed by *04, *05 and *06, consistent with other studies (31, 35, 36). Among participants with at least one *02 allele and across both dose groups, 19/22 vaccine recipients (86%) developed a detectable VRC01-class B cell response (low dose: 9/12 (75%); high dose: 10/10 (100%); fig. S18), comparable with the response induced by eOD-GT8–60mer (22). In contrast, in participants lacking a high-expressing *02 allele, 5/15 (33%) of participants had detectable levels of VRC01-class B cells (fig. S18B). One high dose recipient lacked any permissive allele (*05/*05) and could therefore not generate VRC01-class B cells (table S12). We conclude that GT1.1 can induce VRC01-class IgG B cell responses in healthy adults, irrespective of dose, but at higher frequencies in high dose vaccine recipients and depending on the presence of particular IGHV1–2 alleles.

## Epitope-specific B cells are clonally expanded and hypermutated

A GT vaccine should ideally induce clonally diverse bnAb precursor B cells that persist over time and that can re-expand with subsequent booster vaccinations (3, 37). We observed highly expanded lineages (detection of ≥2 cells within a certain lineage) across apex-specific, CD4bs-specific and VRC01-class B cells, across time points and in all vaccine recipients (Fig. 2K). VRC01-class lineages in particular exhibited a substantial degree of clonal expansion, with only 4% of B cells belonging to a single-member lineage (Fig. 2K, fig. S19A–B). This is in stark contrast with results obtained with eOD-GT8–60mer, in which >80% of VRC01-class lineages were single-member, although clonotype assignment may have been different (22). On average, expanded VRC01-class lineages had greater than 10 members (fig. S19A–C), indicative of extensive activation and proliferation. Moreover, 34% of VRC01-class lineages, accounting for 65% of VRC01-class B cells, were detected at both weeks 10 and 26, suggesting that these lineages may either be long-lived or selected for re-entry in germinal centers (fig. S19D–E).

HIV bnAbs, and in particular VRC01-class bnAbs, often require high levels of somatic hypermutation (SHM) (16, 17). At week 10, SHM was in the range of 0 to 3.7% across apex-specific and CD4bs-specific B cells and across all participants (Fig. 2L, figs. S20–21). At week 26, SHM increased significantly for each class and median levels per participant were significantly higher for VRC01-class B cells (4.9%) than for apex-specific B cells (3.4%) or other CD4bs-specific B cells (4.0%) (Fig. 2L). Moreover, in extreme cases, we detected SHM levels over 10% in VRC01-class B cells, approaching the SHM levels of VRC01-class bnAbs PCIN63 (10–16% SHM) and BG24 (13% SHM) (38–40). We conclude that GT1.1 induced large and expanded B cell lineages, including those of the VRC01-class, that persist over time and accumulate substantial levels of SHM.

## GT1.1-induced VRC01-class BCRs resemble VRC01

**Fig. 3.**
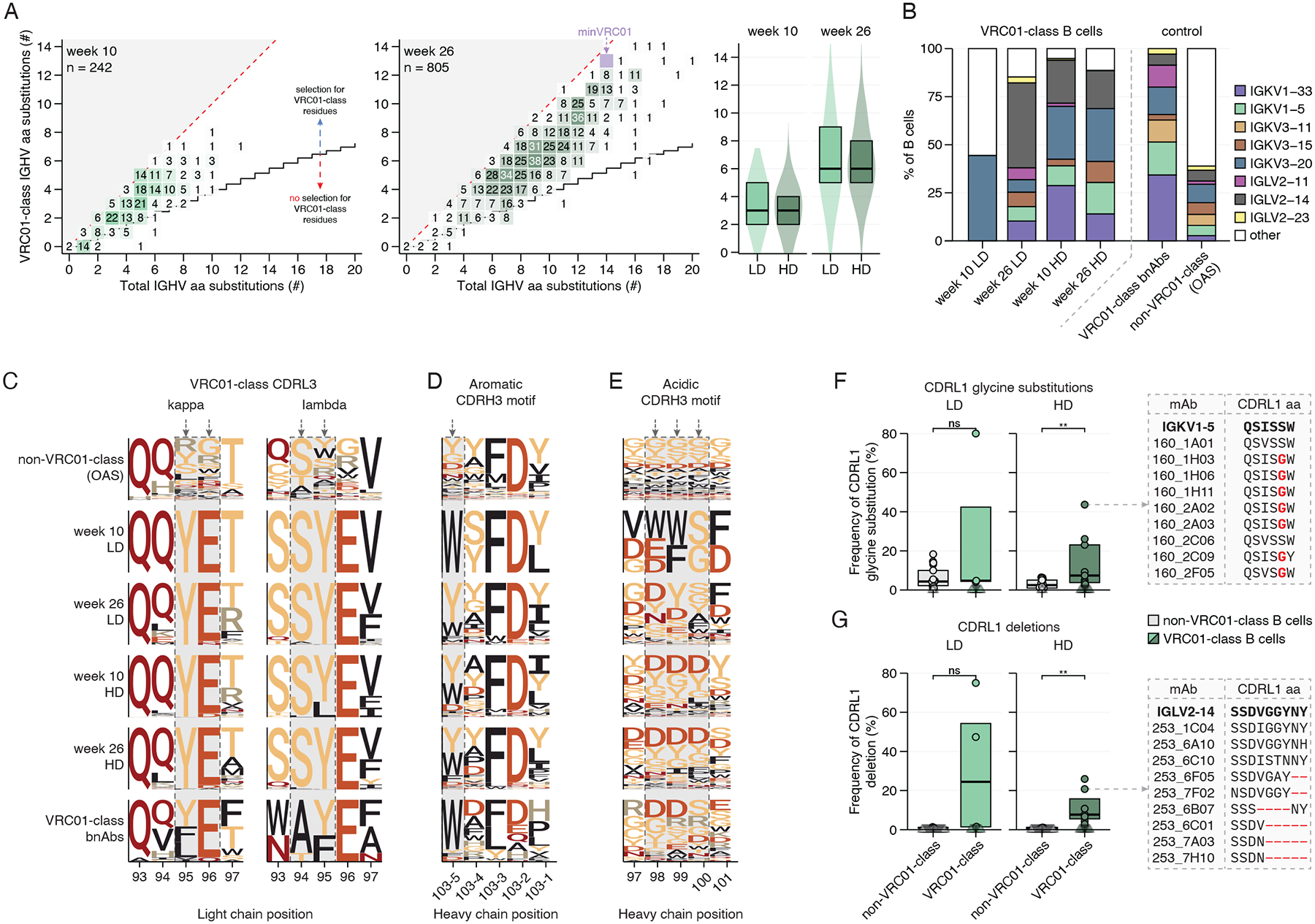
Properties of GT1.1-induced VRC01-class BCRs. **(A)** Left and middle panels: total and VRC01-class amino acid mutations in IGHV1–2 of VRC01-class B cells isolated at week-10 (left) and week-26 (right) for low and high dose recipients combined. The values represent the number of B cells at a particular coordinate. The staggered black line shows the expected level of VRC01-class mutations expected to be introduced by random SHM in IGHV1–2. On track VRC01-class mutations are defined as mutations that are shared with bnAbs VRC01, PGV04, PGV20, VRC-CH31, 3BNC60 or 12A12 (41). Right panel: number of VRC01-class for each dose group. The coordinates of minimally mutated VRC01 (minVRC01) are shown in purple. minVRC01 is a synthetic bnAb that has the minimal number of SHM necessary for broad and potent neutralization (99). **(B)** Proportion of BCRs using light chain variable genes that are associated with VRC01-class bnAbs (colored), from this study, VRC01-class bnAbs and a control data set encompassing >500,000 paired HC/LC antibody sequences (OAS; see Methods) (42). **(C)** Sequence logos for five-residue CDRL3s, non-VRC01-class five-residue CDRL3s from OAS (42), VRC01-class BCRs from this study per group and time point, and VRC01-class bnAbs. The amino acids are colored by physiochemical characteristics and the logos are separated into kappa (left) and lambda (right) light chains. **(D)** Sequence logos for the conserved motif at CDRH3 position 103–5, for each of the groups in panel C. **(E)** Sequence logos for the conserved acidic motif at CDRH3 positions 98–100, for each of the groups in panel C. **(F)** Left panel: frequency of CDRL1 glycine substitutions in non-VRC01-class BCRs (grey) and VRC01-class BCRs (green). Each dot represents one individual. Triangles represent participants without CDRL1 glycine substitutions. Wilcoxon rank-sum test: ns, not significant (p > 0.05); **, p < 0.01. Right panel: example of a lineage showing a high frequency of glycine substitutions (red). **(G)** Left panel: frequency of CDRL1 deletions in non-VRC01-class BCRs (grey) and VRC01-class BCRs (green). Each dot represents one individual. Wilcoxon rank-sum test: **, p < 0.01. Inset: example lineage from one participant showing CDRL1 deletions (red dashes) of various sizes.

VRC01-class bnAbs have specific mutations that confer breadth and potency. After two vaccinations, >90% of VRC01-class BCRs selected specifically for VRC01-class mutations, defined by those that are present in a set of six VRC01-class bnAbs (Fig. 3A, fig. S22) (41). After three vaccinations, the median number of VRC01-class mutations was six (Fig. 3A, fig. S22B). Compared to eOD-GT8–60mer, GT1.1 induced higher levels of VRC01-class mutations across time points (median of 3 *versus* 1 at week 10 across studies; fig. S22C).

VRC01-class bnAbs demonstrate a preference for certain immunoglobulin kappa or lambda light chain (IGKV/IGLV) genes (3). >85% of GT1.1-induced VRC01-class B cells used kappa or lambda variable light chains that have been previously associated with VRC01-class bnAbs (Fig. 3B, fig. S23). Those light chains were thus highly enriched compared to the Observed Antibody Space (OAS) data set that encompasses >500,000 paired BCR sequences (42) (Fig. 3B).

Next, we monitored three regions that are shared between VRC01-class bnAbs: their shorter-than-average five residue CDRL3, a conserved aromatic residue at position 103–5 (previously W100_B_) in the CDRH3, and a negatively charged motif in the CDRH3. First, VRC01-class bnAbs typically have a tyrosine at position 95 (Y95_CDRL3_) and a glutamic acid at position 96 (E_96_), which are important contact residues with the conserved Env loop D (N279_Env_/N280_Env_). Irrespective of their LC usage, the GT1.1-induced VRC01-class B cells almost exclusively (median 100%) contain the typical Y95_CDRL3_ and E96_CDRL3_ residues (Fig. 3C), while control sequences with a five amino acid CDRL3 did not (fig. S24A). Next, the CDRH3 of VRC01-class bnAbs is diverse in terms of both length and composition. Nevertheless, typical features of VRC01-class bnAbs include a conserved tryptophan residue five residues before the end of CDRH3 that contacts Env loop D. GT1.1-induced VRC01-class BCRs had substantially selected for W103–5_CDRH3_ or a similar aromatic amino acid (Y103–5_CDRH3_), with 85% of VRC01-class B cells having either one of these aromatic residues while non-VRC01-class B cells (29%) or sequences from the OAS control data set did not (29%; fig. 3D, fig. S24B). Third, GT1.1 was designed to engage a conserved negatively charged CDRH3 residue in putative VRC01-class precursor B cells (24). 61% of VRC01-class BCRs had an aspartic acid (D) residue in their CDRH3, whereas this frequency was lower in non-VRC01-class B cells from the same participants (22%) or the OAS control data set (22%; fig. 3E, fig. S24C). Our findings suggest GT1.1 elicits VRC01-class B cells with bnAb BCR features.

An important hallmark of VRC01-class bnAbs is their ability to accommodate the N276 glycan, which is achieved through specific SHM events as germline variants of VRC01-class bnAbs do not bind to Env with the N276 glycan (13, 24, 27). These rare but highly necessary SHM events include shortening of the CDRL1 loop or increasing CDRL1 flexibility. After GT1.1 vaccination, glycine substitutions in the CDRL1 of VRC01-class B cells were observed significantly more frequently than in non-VRC01-class B cells, suggestive of positive selection (p = 0.0077, Fig. 3F). In one GT1.1 recipient, 24/55 (44%) of VRC01-class B cells had at least one CDRL1 glycine substitution (Fig. 3F, right panel; fig. S25A–B). Similarly, we frequently observed deletions in the CDRL1 in VRC01-class but not other B cells (Fig. 3G, fig. S25C–D). CDRL1 deletions varied in size, ranging from one to five residues (Fig. 3G, fig. S25). 7/13 (54%) of high dose recipients had at least one VRC01-class lineage with a CDRL1 deletion, indicating that GT1.1 can reproducibly induce and/or select for such rare SHM events (Fig. 3G). At least seven separate VRC01-class lineages had distinct deletions in terms of size and composition, highlighting that these events occurred repeatedly and independently within maturing lineages (Fig. 3G).

## Selected VRC01-class mAbs recognize fully glycosylated Env trimers

**Fig. 4.**
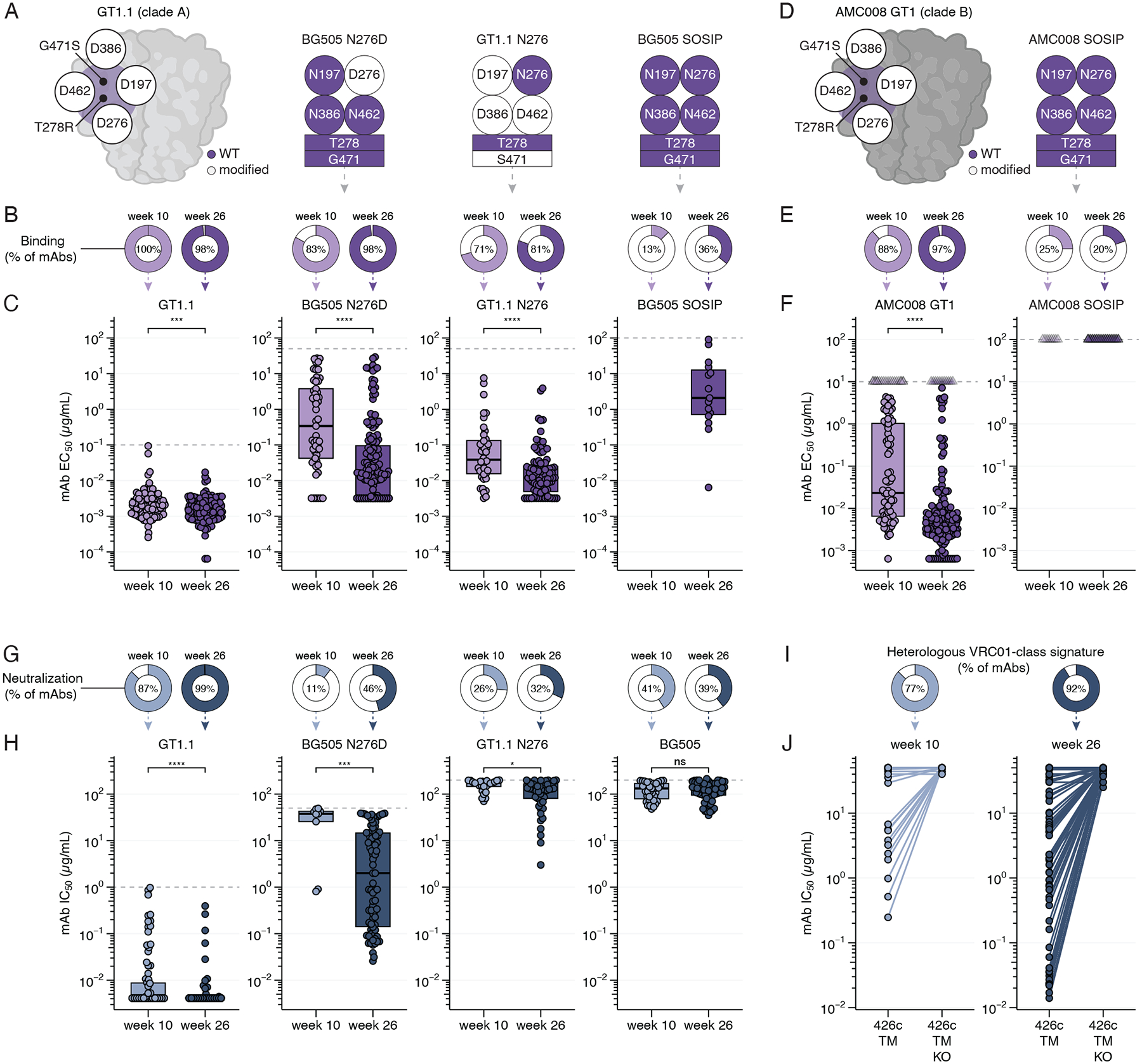
Binding and neutralization by VRC01-class mAbs after GT1.1 vaccination. Each dot represents a single tested mAb. All statistical tests: Wilcoxon rank-sum test: ns, not significant (p > 0.05); *, p < 0.05; ***, p < 0.001. **(A)** Schematic of GT1.1 (grey) highlighting the CD4bs (yellow), surrounding potential N-linked glycosylation sites (white) and GT mutations T278R and G471S. GT1.1 lacks all four CD4bs glycans (absence indicated in white) but does have two GT mutations T278R and G471S. BG505 N276D has three CD4bs glycans (presence indicated in purple), and two GT mutations that are reverted to WT (indicated in purple). GT1.1 N276 has the N276 glycan restored and GT mutation R278T, indicated in purple, but lacks the other CD4bs-proximal glycans. BG505 SOSIP is a wild-type, fully glycosylated trimer. **(B)** Pie charts representing the proportion of mAbs that showed binding to the indicated Env trimer at least ≥3-fold above background at the highest concentration. **(C)** Half-maximal binding concentrations (EC_50_s in μg/mL) of 276 VRC01-class mAbs against the trimers indicated on top of the graphs and in panel A. The dashed lines indicate the maximum mAb concentration tested. **(D-F)** As in panel A-C, but for clade B trimer AMC008. The triangles in panels E indicate mAbs that do not bind the indicated trimer. **(G-H)** As in panel B-C, but depicting the half-maximal inhibitory concentrations (IC_50_ in μg/mL) in a pseudovirus (pseudovirus) neutralization assay against the indicated pseudoviruses in panel A. **(I)** Pie charts representing the proportion of mAbs that showed a ≥2.5-fold decrease in neutralizing concentration (IC_50_) of pseudovirus 426c.TM *versus* its CD4bs KO 426.TM.KO. **(J)** Half-maximal inhibitory concentrations (IC_50_ in μg/mL) in a pseudovirus neutralization assay against 426c.TM and its CD4bs KO 426c.TM.KO.

Vaccines need to drive affinity maturation to enable strong recognition of vaccine antigen(s). However, in a sequential GT vaccination strategy, it is essential that affinity is increased not only to the priming immunogen, but also to candidate shaping and/or polishing immunogens. The latter include antigens that more closely resemble the wild-type (WT) functional Env trimers on circulating viruses. For VRC01-class B cells, there is a need to target conserved CD4bs residues and to accommodate the glycan shield around the CD4bs, in particular the N276 glycan. We expressed a total of 276 recombinant VRC01-class mAbs from high dose GT1.1 recipients and tested their binding to different Env trimers based on both GT1.1 and the prototypical, fully glycosylated BG505 SOSIP trimer (Fig. 4A, figs. S26–S29). The selected mAbs were derived from 11 high dose recipients and 49 unique B cell lineages and can be considered representative of the VRC01-class repertoire (fig. S26). The Env trimers tested included the GT1.1 trimer itself, which lacks four glycans around the CD4bs; the BG505 N276D trimer, which closely resembles WT trimer BG505 SOSIP but lacks the N276 glycan; the GT1.1 N276 trimer, which resembles GT1.1 but has the N276 glycan; and fully glycosylated BG505 SOSIP trimer (Fig. 4A). Both BG505 N276D and GT1.1 N276 proteins are candidate shaping immunogens following GT1.1 priming as they provide steric constraints that may help drive maturation of VRC01-class B cell lineages towards WT Env recognition.

All but two mAbs demonstrated potent binding to GT1.1 with half-maximal binding concentrations (EC_50_s) substantially lower than 1 μg/mL, corroborating the flow cytometry data of the B cells these mAbs are derived from (Fig. 4B–C, fig. S27). mAbs isolated after the third vaccination showed a statistically significant increase in affinity (Fig. 4B), which correlated with an increase in nonsynonymous mutations as well as VRC01-class mutations (fig. S28). Of note, 83% and 98% of mAbs isolated at weeks 10 and 26 were able to bind the BG505 N276D trimer (Fig. 4B–C), albeit with weaker apparent affinities. Furthermore, the median apparent affinity of mAbs from week 26 for BG505 N276D was >30-fold higher than of those from week 10 (0.01 *versus* 0.3 μg/mL), suggesting that the third vaccination drove affinity maturation towards this candidate shaping immunogen. Indeed, higher SHM levels correlated with an increased affinity for BG505 N276D (fig. S28). Thus, GT1.1 not only activates and expands bnAb precursor B cells, but also drives the affinity maturation of VRC01-class mAbs that facilitates their binding to increasingly glycosylated Env trimers.

VRC01-class mAbs isolated at week 10 had a 20-fold lower apparent affinity for GT1.1 N276 than for GT1.1, confirming the N276 glycan as a major obstacle for VRC01-class antibodies to engage the CD4bs (Fig. 4B–C). Nevertheless, the majority of mAbs, in particular at week 26 (81%), could accommodate the N276 glycan to some extent and bind to GT1.1 N276. mAbs from week 26 had a median 5-fold increase compared to mAbs from week 10, suggesting that additional SHM following the third vaccination helped to accomodate the N276 glycan (Fig. 4C). For a substantial subset of mAbs (13% and 36% for weeks 10 and 26, respectively), accomodating N276 translated into recognition of fully glycosylated BG505 SOSIP trimers, albeit with only moderate apparent affinity (median EC_50_: 2 μg/mL, Fig. 4C). Notably, five mAbs exhibited detectable binding to the fully glycosylated heterologous Q23 trimer from clade A (range 4.5–92 μg/mL EC_50_), while none of the mAbs bound detectably to fully glycosylated clade B or C trimers (fig. S29). We next tested binding to clade B trimer AMC008 and its engineered GT1 variant, which includes all GT1 mutations of BG505 GT1 (26). AMC008 GT1 has a lower affinity for VRC01-class precursors than for BG505 GT1.1 (24, 27) and is a candidate shaping immunogen (Fig. 4D). 88% and 97% of VRC01-class mAbs from weeks 10 and 26, respectively, bind this heterologous Env trimer, and mAbs from week 26 bind with a 5-fold higher apparent affinity for AMC008-GT1 than those from week 10 (Fig. 4E–F, fig. S29). None of a selected subset of mAbs bound to fully glycosylated AMC008 SOSIP (Fig. 4E–F, fig. S29). We conclude that GT1.1 vaccination leads to VRC01-class antibodies that can recognize increasingly glycosylated trimers, which may serve as shaping immunogens to drive such a response towards broad recognition of diverse and fully glycosylated Envs.

## Selected VRC01-class mAbs neutralize wild-type viruses

While not an explicit goal of a GT priming vaccination, neutralization of pseudoviruses with different glycosylation levels provides insights into the maturation level of bnAb precursor mAbs. To assess the neutralizing potential of VRC01-class mAbs we used pseudovirus expressing full length Envs with antigenic properties similar to the aforementioned SOSIP trimers. The GT1.1 pseudovirus was neutralized by 87% and 99% of the VRC01-class mAbs isolated at weeks 10 and 26, respectively (Fig. 4G). The median half-maximal inhibition concentrations (IC_50_s) were at the lower limit of detection (<0.004 μg/mL), illustrating strong recognition of the CD4bs (Fig. 4G–H). Hence, we further tested their neutralization of the more glycosylated pseudoviruses BG505 N276D, GT1.1 N276, and the fully glycosylated BG505 pseudovirus (Fig. 4G–H). Consistent with the binding data, a subset of mAbs could accommodate the CD4bs-proximate glycans and neutralize the BG505 N276D and GT1.1 N276 pseudoviruses. However, the number of mAbs that neutralized these viruses and their potency were lower than the number and potency of mAbs detectably binding to the matching Env trimers (Fig. 4G–H). Nevertheless, 39% of VRC01-class mAbs neutralized the fully glycosylated, tier-2 pseudovirus BG505.T332N, albeit weakly (median IC_50_ 133 μg/mL; range 35–198 μg/mL; Fig. 4H). We observed weak but detectable neutralization of clade B tier 2 pseudovirus AMC011 and consensus pseudovirus ConM (IC_50_ > 100 μg/mL) by a subset of mAbs (fig. S30A) (43, 44).

We next monitored VRC01-class signature neutralization using 426c.TM, and its CD4bs KO variant, derived from the clade C virus 426c (Fig. 4I–J) (45). Notably, 77% and 92% of mAbs from week 10 and week 26, respectively, that neutralized 426c.TM exhibited a ≥2.5-fold decrease to the 426c.TM KO pseudovirus, indicative of a heterologously neutralizing CD4bs response (Fig. 4I–J). We further tested 45_01dG5, a WT virus naturally lacking N276 from the PWH from which bnAb VRC01 was isolated, and its closely related lineage member 45_01dH1, in which the N276 glycan is restored (46). A subset of VRC01-class mAbs after the third GT1.1 vaccination neutralized N276-lacking pseudovirus 45_01dG5 (17% of mAbs, range 0.07–34 μg/mL) and neutralizing concentrations were higher against N276-containing pseudovirus 45_01dH1 (13% of mAbs, range 13–99 μg/mL) (fig. S30B). Furthermore, 68% of mAbs from week 26 had potent neutralizing activity against tier 2 clade B pseudovirus 1HD2−051916−B4−S62 (range 0.003–15 μg/mL) that also naturally lacks the N276 glycan (47). Two other tier 1B and tier 2 pseudoviruses lacking the N276 glycan were neutralized by a small subset of tested mAbs (fig. S30C). Thus, GT1.1 vaccination elicits VRC01-class mAbs that acquire the capacity to neutralize the fully glycosylated BG505.T332N pseudovirus as well as diverse heterologous WT pseudoviruses, in particular ones that naturally lack the N276 glycan.

## VRC01-class mAbs from GT1.1 vaccination structurally resemble bnAb VRC01

**Fig. 5.**
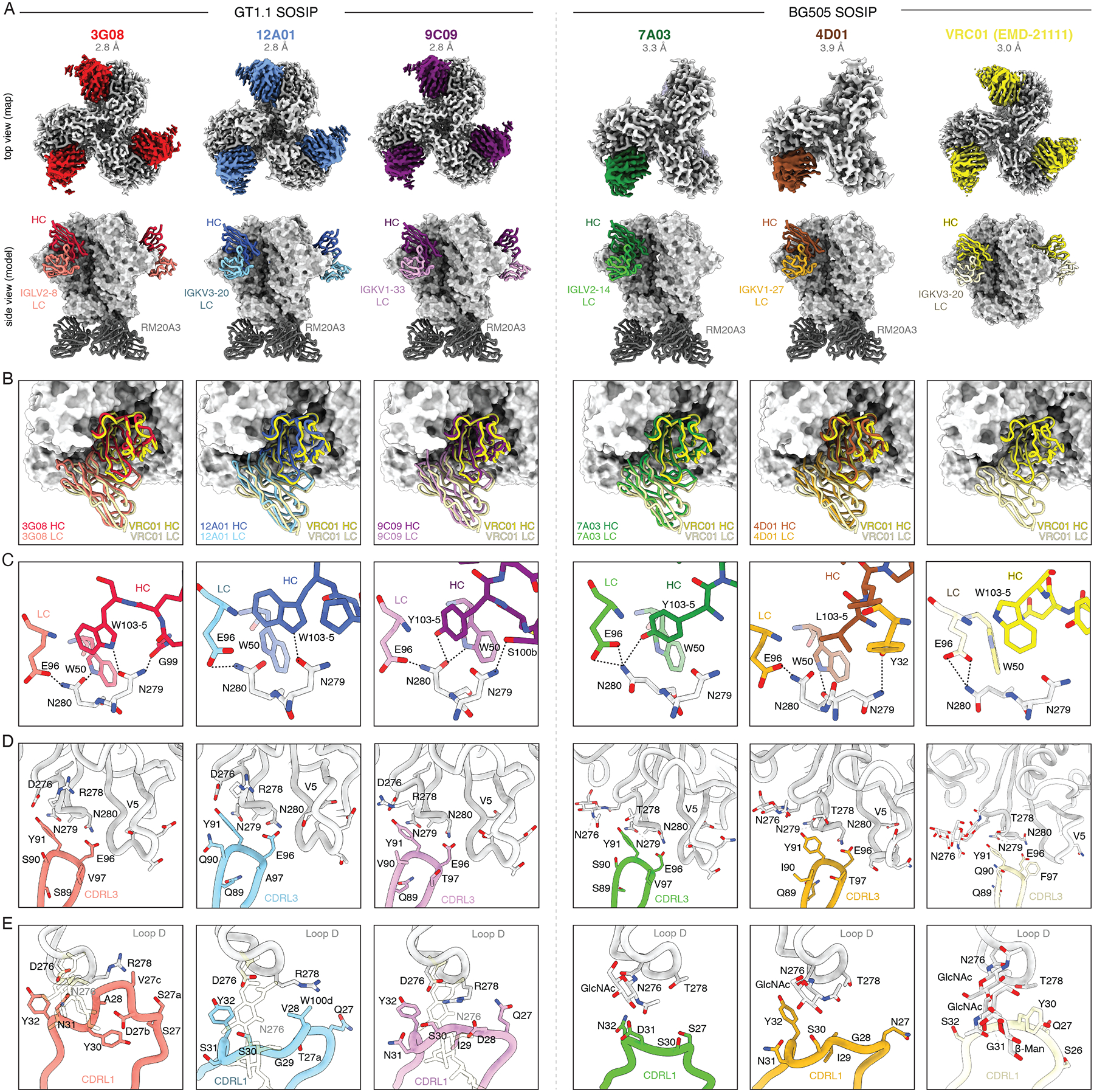
GT1.1-induced VRC01-class mAbs structurally resemble VRC01-class bnAbs and accommodate CD4bs glycans. **(A)** Segmented cryo-EM maps (top) and atomic models (bottom) of five mAbs in complex with either GT1.1 (left) or fully glycosylated BG505 SOSIP (right) compared to a published cryo-EM map and model of VRC01-bound BG505 SOSIP (EMD 21111, PDB 6V8X). The resolution of each structure is shown below the mAb name. For the models, Env is shown as surface representation, and antibody chains are shown as ribbons. **(B)** Comparison of overall approach angle and orientation of GT1.1-elicited VRC01-class mAbs and VRC01 (PDB 6V8X). **(C)** Key molecular interactions between CD4bs residues N279/N280 and each VRC01-class mAb. Putative hydrogen bonds (atomic distance < 3.4 Å) are shown as dashed lines. Antibody numbering follows the Kabat scheme. **(D)** Comparison of CDRL3 loops and their loop D contacts, including the conserved Y_95_E_96_ motif (see also fig. 3C). **(E)** Overview of CDRL1 interactions with Env loop D. For GT1.1 complexes, which lack the N276 glycan, a semitransparent glycan is shown based on alignment of a VRC01-bound structure (PDB 6V8X).

The GT strategy for inducing VRC01-class bnAb precursors is rooted in the hypothesis that these antibodies target the CD4bs with a similar angle of approach to VRC01 and use the same conserved contact residues. To verify that VRC01-class mAbs elicited by GT1.1 do not just exhibit similar sequence features to VRC01-class bnAbs but also target the CD4bs in a similar manner and with the same atomic contacts, we selected five mAbs for further structural characterization. These mAbs represent the diversity of the GT1.1-induced VRC01-class repertoire: they were isolated from four distinct high dose recipients and use five different light chains, three of which were previously associated with VRC01-class bnAbs (IGKV1–33, IGKV3–20 and IGLV2–14) and two that were not (IGLV2–8 and IGKV1–27, fig. 5A). One mAb (3G08) was isolated from B cells at week 10, the other four at week 26. Moreover, these mAbs had different levels of SHM and VRC01-class mutations and had differential binding and neutralization patterns (fig. S31). First, we established the binding kinetics and affinities of the mAbs using surface plasmon resonance (SPR, figs. S32–S33, tables S12–S14). Corroborating the ELISA data, all five selected mAbs bound strongly to GT1.1, with 12A01 and 7A03 having the highest affinity with *K*_D_ in the picomolar range. Binding of 12A01, 3G08, and 9C09 to BG505 SOSIP and/or GT1.1 N276 was detectable but too weak for accurate modeling (figs. S32–S33, tables S12–S14). In contrast, 7A03 and 4D01 had high affinity for BG505 SOSIP, with *K*_D(conf)_ values of 8.3 and 2.4 nM, respectively, as fitted with a conformational-change model (figs. S32–S33, tables S12–S14). Based on these results, we determined the high-resolution structures of three mAbs, 12A01, 3G08, and 9C09, in complex with GT1.1, and 7A03 and 4D01 in complex with fully glycosylated BG505 SOSIP using cryo-electron microscopy (cryo-EM) (Fig. 5, figs. S34–S37, table S15). Moreover, we determined the structures of all unliganded Fabs and four out of five mAbs in complex with GT vaccine candidate eOD-GT8 by X-ray crystallography (figs. S35–S37, tables S16–S17).

mAbs 3G08, 12A01 and 9C09 bound GT1.1 with a 3:1 stoichiometry, while 7A03 and 4D01 bound BG505 SOSIP with a 1:1 stoichiometry (Fig. 5A). Overall, whether unliganded, bound to eOD-GT8, to GT1.1 or to BG505 SOSIP, the mAb structures agreed extremely well with each other and revealed that the mAbs targeted Env residues that are commonly targeted by VRC01-class bnAbs (Fig. 5, figs. S34–S37). One exception was mAb 9C09, for which the unliganded CDRH3 was predicted to clash with loop D, while the Env-bound CDRH3 adopted a conformation that enabled the accommodation of and binding to loop D (fig. S35B). All mAbs aligned extremely well with the orientation and angle of VRC01, with minor differences in light chain loop length and orientation (Fig. 5B, fig. S35C–D). All VRC01-class mAbs targeted conserved loop D residues N279_Env_/N280_Env_: two mAbs, 3G08 and 12A01 use the canonical CDRH3 residue W103–5_CDRH3_ to form hydrogen bonds with N279_Env_ (Fig. 5C, fig. S36C–D). Two other mAbs, 9C09 and 7A03, have an atypical Y103–5_CDRH3_ which is not positioned to form a hydrogen bond with N279_Env_. Instead, they contact neighboring residue N280_Env_, which also forms a hydrogen bond with E96_CDRL3_ (Fig. 5C, fig. S36C–D). mAb 4D01, which has a leucine at 103–5_CDRH3_, maintains hydrophobic interactions with loop D but does not have the ability to contact N279_Env_/N280_Env_ (Fig. 5C, fig. S36C–D). All mAbs bore a typical CDRL3 sequence with a conserved Y95_CDRL3_ and E96_CDRL3_ residues, and were found to be indistinguishable from each other and from VRC01 with respect to their atomic contacts, despite having different flanking residues (Fig. 5D, fig. S36E).

When inspecting the GT1.1-bound structures of the three mAbs that did not have measurable affinity for fully glycosylated BG505 SOSIP, we found that these mAbs would clash with the N276 glycan (Fig. 5E). In contrast, 7A03 and 4D01 are able to accommodate the N276 glycan in the context of a fully glycosylated trimer (Fig. 5E), and the first sugar of the N276 glycan was visible in the structures. 7A03 shares its light chain V gene (IGLV2–14) with bnAb VRC-PG20, which has a six-residue deletion in its CDRL1 to help accommodate the N276 glycan (48). Strikingly, many 7A03 clonal lineage members have multi-residue CDRL1 deletions of varying sizes, despite never having been exposed to the N276 glycan (Fig. 3G). Compared to its inferred germline, 7A03 itself has a five-residue CDRL1 deletion which removes potential clashes with the N276 glycan (Fig. 5E). In contrast, 4D01 does not have CDRL1 deletions but has a flexible germline CDRL1 to accommodate the N276 glycan (Fig. 5E). Both mAbs do appear to displace the N276 glycan, and more so than VRC01 does, as the glycan moves towards the trimer core by over 60° relative to the unliganded state and over 40° relative to the VRC01-bound state, which may at least in part explain why they are not yet fully mature bnAbs (fig. S37). We conclude that GT1.1 vaccination selects for CDRL1 characteristics that help accommodate the N276 glycan, without the need for the N276 glycan to be present in the vaccine.

## Discussion:

Despite important recent advances in HIV therapy and prevention with the development of long-acting antiretrovirals (49), an effective vaccine remains crucial to ending the epidemic. While HIV vaccine research has had considerable impact on accelerating the development of vaccines against other pathogens, such as SARS-CoV-2 (50–53) or respiratory syncytial virus (54–56), the quest for an HIV vaccine itself poses unprecedented challenges. Germline-targeting (GT) vaccine strategies to induce bnAbs represent a rational approach towards overcoming these challenges. The GT approach is premised on rational design of priming immunogens that can recruit rare precursor bnAb B cells, select for bnAb-like features while expanding them and generate immunological memory that can subsequently be boosted and matured by increasingly native-like shaping and polishing immunogens. One advanced GT strategy relies on presentation of a fragment of the gp120 subunit of Env multivalently on a nanoparticle (eOD-GT8–60mer) in order to activate a large number of precursors, a strategy which has demonstrated potential in preclinical and clinical studies (14, 22). In contrast, our strategy uses stabilized Env trimers that more closely mimic the structure of virus-associated Env (24, 57), which should facilitate the selection of bnAb precursors with angles of approach compatible with binding to WT Env, as well as enable the targeting of multiple epitopes. Here, in the first test of a GT Env trimer in humans, we found that BG505 SOSIP GT1.1 gp140 formulated with AS01_B_ had a good safety profile and induced polyclonal antibody responses towards multiple epitopes, including the CD4bs epitope. We demonstrated that in the majority of vaccine recipients a substantial fraction (~30%) of CD4bs-specific IgG memory B cells is of the VRC01-class. Furthermore, these VRC01-class BCRs have favorable bnAb-like sequence features that consistently conferred high affinity to increasingly glycosylated trimers. Finally, GT1.1-induced VRC01-class mAbs are structurally extremely similar to VRC01-class bnAbs. These findings demonstrate the potential of a GT trimer platform to induce bnAbs of the VRC01-class and potentially other classes.

After three GT1.1 vaccinations, we detected VRC01-class memory B cells at high frequency (1 in 2,500 of all IgG memory B cells), despite being unable to detect GT1.1-specific naive VRC01-class B cells at the pre-vaccination time point (fig. S12). Similarly, eOD-GT8–60mer induced substantially stronger VRC01-class precursor expansion after a single vaccination in a phase 1 clinical trial than it did in two distinct preclinical knock-in mouse experiments (22, 58, 59). Our findings nuance established affinity threshold and precursor frequency requirements for the activation of rare bnAb precursor B cells derived from preclinical animal models (58, 60, 61), and are consistent with the observation that low precursor frequencies are sufficient to enable recruitment to germinal centers (62). Although precursor frequency, affinity and avidity are undeniably critical parameters in germinal center reactions, our results demonstrate that immunogens with lower affinities for VRC01-class precursors *in vitro* may induce similar responses to high-affinity immunogens in humans (22), albeit with a lower response rate. A high dose GT1.1 vaccination (300 μg) demonstrated superiority over a low dose in a number of endpoints such as CD4bs-specific serum antibodies and the frequency of CD4bs-specific memory B cells.

While high B cell frequencies, high response rates, and strong mAb binding to the vaccine immunogen are indicators of a successful GT prime, the ability to bind to candidate shaping and polishing immunogens is also critical. After three GT1.1 vaccination, the majority of almost 300 representative VRC01-class mAbs recognized our top candidate shaping immunogens BG505 N276D, GT1.1 N276 and AMC008 GT1. These data also indicate that these mAbs have evolved the ability to deal with i) all CD4bs glycans besides the N276 glycan; and/or ii) the N276 glycan and/or iii) a heterologous CD4bs epitope. Approximately one third of week 26 mAbs had detectable binding to fully glycosylated Env trimers, with a subset of these mAbs having nanomolar affinity for BG505 SOSIP. Hence, some GT1.1-elicited VRC01-class BCRs acquired mutations that confer strong binding to native trimers with intact glycan shields. Although neutralization is not the most relevant endpoint after a GT priming vaccination, a subset of VRC01-class mAbs neutralized heterologous pseudoviruses lacking the N276 glycan or homologous BG505 pseudovirus, demonstrating that GT1.1-elicited VRC01-class mAbs are substantially advanced on the maturation path towards bnAbs. Based on the totality of our observations, high dose recipients from this study were invited to enroll in follow-up studies (NCT05863585, IAVI C107; and NCT05983874, IAVI C110) to receive two booster vaccinations with BG505 SOSIP. The goal of this ongoing study will be to assess whether and to what extent shaping or polishing events take place.

Our analyses concentrated on the induction of VRC01-class B cells because targeting such cells was the focus of the GT1.1 design (24), and a pre-defined endpoint of the trial. However, GT1.1 also induced on-target memory B cell responses against the trimer apex, consistent with design modifications to that region (26), as well as CD4bs responses that were not of the VRC01-class. We are further analyzing all of these antibody responses. Furthermore, EMPEM analyses revealed that sera from a substantial number of recipients contained polyclonal antibodies to a region around the fusion peptide (FP) where epitopes for bnAbs ACS202, VRC34 and PGT151 are located (63–65). While GT1.1 was not specifically designed to target FP-directed bnAb precursors, this apparent intrinsic property could be exploited further. In addition to these desirable responses, EMPEM also revealed unwanted specificities. First, all high dose GT1.1 recipients had a response against the trimer base, a notorious non-neutralizing neo-epitope on Env trimer subunit vaccines (24, 66, 67). The trimer base is naturally occluded by the viral membrane in virus-associated Env and thus is irrelevant for virus neutralization. Similarly, the majority of high dose recipients developed a response towards an epitope that is likely the result of the absence of potential *N*-linked glycosylated sites (PNGS) N241/N289 (24, 68). Reducing the immunogenicity of these epitopes through the addition of PNGS or membrane presentation is a focus of future studies.

This study provides proof-of-concept for Env trimer-based GT approaches to activate bnAb precursors and induce affinity maturation on the path towards mature bnAbs. Evaluating whether and to what extent booster immunogens can shape and polish bnAb lineages is now a priority (69). Our structural analyses of diverse VRC01-class mAbs demonstrate that precise targeting of bnAb precursors with atomic-level accuracy can now be achieved, heralding a new era of vaccine design.

## Materials and Methods:

### Study design

The IAVI C101 study (NCT04224701), was a phase 1, randomized, double-blind, placebo-controlled, dose-escalation trial to evaluate the safety, tolerability and immunogenicity of the GT1.1 gp140 vaccine (GT1.1), adjuvanted with AS01_B_, in adults without HIV infection and in general good health. Participants were randomized in a 5:1 vaccine-to-placebo ratio to receive intramuscular injections at 0, 8, and 24 weeks of either 30 μg (group 1, low dose) or 300 μg (group 2, high dose) of GT1.1 with 50 μg of AS01_B_, or a placebo. Each vaccination was administered in the deltoid muscle of the same arm. The placebo was the buffer (Tris-NaCl, pH 7.5) used in the vaccine. The CONSORT diagram is shown in fig. S1.

The primary objective of the study was to evaluate the safety and tolerability of the adjuvanted GT1.1 vaccine at two different dose levels; the secondary objective was to assess binding antibody responses following vaccinations. The primary endpoints were the proportion of participants with grade 2 or greater solicited adverse events (AEs), investigational product (IP)-related unsolicited AEs, grade 2 or greater unsolicited AEs, IP-related serious adverse events (SAEs) and potential immune-mediated diseases (pIMDs). The secondary endpoints were the frequency and magnitude of binding antibody responses to GT1.1 following each vaccination. Key exploratory immunologic objectives were to determine the ability of GT1.1 to expand rare bnAb B cell precursors, including the frequency of GT1.1-specific, CD4-binding site (CD4bs)-directed, VRC01-class and trimer apex-directed memory B cells. Additional exploratory analyses included assessments of: serum neutralization signatures, the neutralization activity of selected vaccine-elicited, GT1.1-specific CD4bs mAbs, the mapping of GT1.1-reactive antibody responses, and the analysis of mutations in vaccine-elicited memory B cells.

### Study products

BG505 SOSIP.v4.1 GT1.1 gp140 (GT1.1) was produced under current good manufacturing practice (cGMP) using similar methods as used for BG505 SOSIP.664, as described previously (28). Briefly, BG505 SOSIP.GT1.1v4.1 gp140 (GT1.1) was produced in Chinese hamster ovary (CHO) cells by recombinant DNA technology. After growth in a fed-batch 200 L production bioreactor, the clarified harvested supernatant, containing GT1.1, was further concentrated using ultra-filtration and processed further to generate purified bulk drug substance: The bulk drug substance was formulated at 3 mg/mL in 20 mM Tris, pH 7.5, 100 mM NaCl, and stored at ≤ −65 °C. The drug substance was further sterile filtered and filled in 2R Schott vials at 0.55 ± 0.05 mL to produce the drug product at 2 mg/mL in 20 mM Tris, pH 7.5, 100 mM NaCl. The drug product is stored at ≤ −65 °C. AS01_B_ is a TLR4 agonist 3-O-desacyl-4-monophosphoryl lipid A (MPL) and QS-21 (*Quillaja saponaria Molina,* fraction 21) formulated with liposomes in 0.65 mL of phosphate buffered saline at pH 6.1. The drug product was manufactured under cGMP conditions and provided by GSK.

### Participants and randomization

We enrolled adult participants without HIV-1 infection and in general good health, aged 18 to 50 years, who were able to provide written informed consent and, as part of the informed consent process, were willing to undergo HIV-1 testing and use an effective contraception method. Forty-seven participants who met all eligibility criteria were randomly assigned to receive vaccine or placebo within one of the two dosing groups according to a randomization schedule prepared by the statisticians at the Data Coordinating Center (DCC) prior to the start of the study. Participants were assigned a specific allocation number as they enrolled into the data entry system. A randomization key was provided to the unblinded site pharmacist by the DCC. Participants were enrolled consecutively in Groups 1 and 2. The first administration of the first 3 participants in each dose group occurred at least 1 day apart, and not more than 3 participants were administered product in the first week of initiating administrations in each dose group. Following a favorable review of safety data by the protocol safety review team (PSRT) from days 0–7 following first IP administration for all volunteers in group 1, IP administration was initiated in group 2. Group 1 included 20 low dose (30 μg) vaccine and 4 placebo recipients, while group 2 included 19 high dose (300 μg) vaccine and 4 placebo recipients. Participants were enrolled at three clinical sites: Rockefeller University (RU), Amsterdam University Medical Centers, location Amsterdam Medical Center (AMC), and George Washington University (GW). Participant demographics are presented in table S1. All enrolled participants (47/47) received the first IP administration at week 0. One participant in the low dose group was lost to follow up at week 3 (prior to the second IP administration); a second participant in the low dose group withdrew from the study after completing week 18 (between the second and third IP administrations); 1 participant in the high dose group did not receive the third IP administration, but completed remaining follow up visits; and 1 participant in the high dose group was lost to follow up after completing Week 48 (after receiving 3 IP administrations).

### Oversight and blinding

The trial was conducted under an Investigational New Drug (IND) application (IND #19360) submitted to the US Food and Drug Administration and a clinical trial application submitted to the Central Committee on Research Involving Human Subjects (CCMO) in the Netherlands (NL72440.000.20). The trial adhered to IAVI standard operating procedures in accordance with the guidelines formulated by the International Committee on Harmonization for Good Clinical Practice in clinical studies. It also complied with applicable local standards and regulatory requirements including review and approval by the institutional review boards at RU, AMC and GW. The trial was overseen by a PSRT and independent safety monitoring committee (SMC). Study site investigators, staff, and participants were blinded in terms of allocation to vaccine *versus* placebo. An unblinded study pharmacist at each site was responsible for vaccine preparation and accountability. Staff performing immunological assays were blinded to the study product allocation. Staff carrying out bioinformatic and statistical analyses were unblinded, which enabled analyses to be carried out during the trial and led to early planning and preparation for follow-on trials.

### Safety and tolerability monitoring

Study participants returned to the study sites for follow up one day after the first vaccination, weekly at weeks 1 to 3, 9 to 11, and at weeks 25, 26 and 48. Participants recorded injection site and systemic reactogenicity (solicited AEs) using a memory aid from day 0 through day 7 after each vaccination. At each vaccination visit, vital signs were measured by study staff prior to vaccination and again at least 30 min after vaccination.

Safety and tolerability of the IP were monitored during the trial by site investigators, the sponsor’s medical monitor and the PSRT for, at a minimum, the first 7 days after the first vaccination for all participants in the low dose group (group 1) and, later, the higher dose level (group 2). Reactogenicity events were reported from day 0 through day 7 after each administration. Unsolicited AEs were collected up to day 28 after each vaccination. Participants were followed for 12 months after the final IP administration for safety endpoints including SAEs and pIMDs. The latter were a subset of AEs that included autoimmune diseases and other inflammatory and/or neurologic disorders of interest that may not have had an autoimmune etiology. AEs were categorized using the Medical Dictionary for Regulatory Activities Terminology, version 2.1 (MedDRA) System Organ Class (SOC) and Preferred Term (PT). All reported AEs were graded using the National Institutes of Allergy and Infectious Diseases Division of AIDS (DAIDS) table for Grading the Severity of Adult and Pediatric Adverse Events, Version 2.1, July 2017 (https://rsc.niaid.nih.gov/sites/default/files/daidsgradingcorrectedv21.pdf).

### Immunological sample collection and storage

Leukapheresis was performed at three time points, once prior to the first vaccination at weeks −4 and at study weeks 10 and 26 (~14 days after the second and third vaccinations, respectively). Peripheral blood mononuclear cells (PBMCs) were collected at weeks −4, 1, 2, 3, 8, 9, 10, 11, 24, 25, 26 and 48 after the first vaccination by leukapheresis or by venipuncture (whole blood with ACD anticoagulant), and were isolated by density gradient centrifugation and cryopreserved as aliquots of 20 × 10^6^, 50 × 10^6^ or 100 × 10^6^ cells. Separately, PBMC aliquots obtained by a single leukapheresis from an unvaccinated volunteer without HIV infection served as an internal negative control for the flow cytometry panel and probe staining in every experiment. Serum samples were collected at the same time points. Ultrasound-guided fine needle aspirations (FNAs) of axillary lymph node(s) were performed at two time points, approximately 21 days after the first and the second vaccinations (70). The procedure was performed by a radiologist using ultrasound guidance to avoid needle insertion into any adjacent structures. Lymph node (LN) FNA samples were stored on wet ice and frozen within four hours of collection.

### Power analysis and rationale for trial size

The group sizes for the study were selected to investigate the primary hypothesis that the investigational product, GT1.1, would induce CD4bs-specific memory B cells in at least 70% of participants in at least one of the vaccine dose groups, as well as the need to have enough endpoints for further characterization of the induced memory B cell repertoire. The study was powered to have a high probability of observing at least 8 IP-receiving participants with detectable CD4bs-specific memory B cells in the low (group 1) or high (group 2) dose groups, assuming that the true response rate for this class of B cells is at least 70%. We assumed there would be an ~20% rate of missing data, resulting in n=16 evaluable immunogenicity samples from recipients of the IP in groups 1 and 2. Hence, at least 8 samples positive for CD4bs-specific memory B cells would be required for a 95% confidence interval of the observed rate to be consistent with a true rate of 70% (95% CI for an observed rate of 8/16 = 50% is 28% to 72%).

### Schedule of procedures

The full schedule of procedures is given in table S5.

### Protein production for BAMA and B cell analytics

#### Trimer production

Free-floating HEK293F cells (Invitrogen, R79007) were cultured in 293F Expression Medium with 3 μL/mL Antibiotic-Antimycotic (Thermo Fisher Scientific, 15240062). 1 L of 293F cells was prepared in a 2,000 mL baffled vented flask (Corning, 431256) at 0.5 × 10⁶ cells/mL and used for transfection the next day when the density had reached 1.0 × 10⁶ cells/mL. All proteins were produced in HEK293F cells using a protocol that has previously been described (24, 57, 71). Briefly, 250 μg of expression plasmid and 62.5 μg of Furin expression plasmid where filtered and mixed with PEI-Max (Polysciences, 24765) in a 1 in 3 ratio in Opti-MEM reduced serum medium (Gibco, 31985062), the solution was then mixed and left to incubate for 30 min at room temperature, after which it was added to the 293F cells and left to cultivate for 6–7 days. The transfection supernatant was harvested and spun down by centrifugation and vacuum filtered through 0.2 μm filters. A column made of CBNr-activated Sepharose 4B beads (Cytiva, 17043001) coupled to bnAb PGT151 was then added to the filtered supernatant and rolled overnight at 4 °C. The supernatant was poured on to a chromatography column and washed with 2 column volumes of Wash buffer (0.5 M NaCl, 20 mM Tris, pH 8.0), after which elution buffer (3 M MgCl_2_) was used to elute the protein. The eluate was then neutralized with TN75 buffer (75 mM NaCl, 10 mM Tris, pH 8.0) in a 1:1 ratio. The solution was then concentrated and buffer exchanged to TN75 using a 100 kDa cut-off spin filter (Sartorius). Concentrated proteins were filtered by a 0.22 μm filter (Costar, 98231-UT-1) and measured with UV280 absorbance using calculated extinction coefficients. Proteins were aliquoted and stored at −80 °C.

#### Antibody production

Recombinant antibodies were produced in HEK293F cells as described above and in (72). 156 μg of heavy chain plasmid DNA and 156 μg of light chain plasmid DNA were filtered and mixed with PEI-Max (Polysciences, 24765) in a 1:3 ratio in Opti-MEM reduced serum medium (Gibco, 31985062). The solution was then mixed and left to incubate for 30 minutes at room temperature, after which it was added to the 293F cells and left to cultivate for 6–7 days. The transfection supernatant was harvested, spun down by centrifugation, and vacuum filtered through 0.2 μm filters. Immobilized Protein G Agarose (Pierce, 20397) was added to the supernatant and left to roll overnight at 4 °C. The supernatant was poured onto a centrifuge column (Pierce, 89898) and washed twice with PBS, pH 7.2. The beads were then eluted with elution buffer (0.1 M glycine, pH 2.5), after which the pH was neutralized with 1 M Tris, pH 8.6, in a 9:1 ratio. The solution was then concentrated and buffer-exchanged to PBS using a 100 kDa cut-off spin filter (Sartorius). Concentrated proteins were filtered through a 0.22 μm filter (Costar, 98231-UT-1) and measured with UV280 absorbance using the standard IgG extinction coefficient.

#### Gel electrophoresis

Proteins and antibodies were analyzed using sodium-dodecyl sulfate-polyacrylamide gel electrophoresis (SDS-PAGE) and stained with PageBlue Protein staining solution (Thermo Fisher Scientific, 24620). For non-reduced samples, a mix was made with purified protein, 4 X loading buffer (6 μL 0.5M Tris-HCl pH 6.8, 4.8 μL glycerol, 9.6 μL 10% SDS, 1.2 μL bromophenol blue 1% w/v, 2.4 μL ultrapure water); for reduced samples 1.2 μL of 1M dithiothreitol (DTT) was added. All samples were heated at 98 °C for 10 min. The samples were run on a 10%−20% gradient gel for 90 min at 125 V (0.03 A) using 25 mM Tris, 192 mM glycine, 0.05% SDS as the running buffer. To verify trimeric structure, proteins were analyzed with blue native (BN)-PAGE and stained with colloidal blue staining (Life Technologies, LC6025). The samples were prepared with a 4x MOPS buffer (200 mM MOPS, 200 mM Tris, pH 7.7) and run on a NuPAGE 4%−12% Bis-Tris gel (Novex, NP0321BOX) for up to 60 min at 200 V (0.03 A) using cathode (Invitrogen, NB2001) and Anode (Invitrogen, NB2002) buffers as the running buffers.

### Immunological assays

#### Serum binding analysis by BAMA

Serum HIV IgG responses against seven antigens (BG505 SOSIPv4.1-GT1.1 AviB (GT1.1), BG505 SOSIPv8.1-GT1.1 Super-KO AviB (GT1.1 super KO), BG505 SOSIPv8.1-GT1.1 Apex-KO AviB (GT1.1 apex KO), BG505 SOSIPv8.1-GT1.1 CD4bs-KO AviB (GT1.1 CD4bs KO), BG505 SOSIP v5 base KO + 613T aviB (BG505 SOSIP base KO), BG505 SOSIP.664 AviB (BG505 SOSIP), and off-target gp41) were measured on a Bio-Plex instrument (Bio-Rad) using a standardized custom HIV Luminex assay (73). The readout is background-subtracted median fluorescence intensity (MFI), where background refers to the antigen-specific plate-level control (*i.e*., a blank well containing antigen-conjugated beads run on each plate). The positive controls were human immunodeficiency virus immunoglobulin (HIVIG), broadly neutralizing antibodies (bnAbs) PGT145 IgG mAb (quaternary V2 Apex-specific IgG), PGT151 IgG mAb (gp120/gp140 interface trimer-specific IgG), VRC01 IgG mAb (CD4bs-specific IgG bnAb), 2G12 IgG mAb (glycan-specific IgG mAb). Germline forms of the following CD4bs-and V2-Apex-specific bnAbs were used as controls: CD4bs-specific mAbs germline VRC01, germline 12A12, and germline NIH45–46 and V2-Apex-specific mAbs germline PG9, germline PG16, and germline CH01. In addition, the following IgG mAbs were utilized as controls to verify the expected binding pattern to the various SOSIP trimers: PGT125 (V3-specific bnAb), F105 (CD4bs-specific non-bnAb), 17BCL2 (CD4 induced-specific non-bnAb), and 19B CL2 (V3-specific non-bnAb). 7B2 IgG mAb (gp41-specific) and RM19R IgG mAb (base-specific) were used as controls for binding to off-target specificities. Blank beads and blank wells (antigen-coupled beads + detection antibody) served as negative controls for non-specific binding. If the blank bead negative control exceeded 5,000 MFI, the sample was repeated. If the repeated value exceeded 5,000 MFI, the sample was excluded from analysis due to high background. MFI values were measured from serum samples at dilutions of 1:50, 1:250, 1:1,250, 1:6,250, 1:31,250, and 1:156,250.

#### Serum neutralization analysis by pseudovirus neutralization assays

Virus neutralization was assessed in TZM-bl cells by using Env-pseudotyped viruses as described previously (74). Neutralization titers (inhibitory dilution of serum samples) or neutralizing concentrations (inhibitory concentrations of mAbs in μg/mL) are given for a 50% reduction in Tat-induced luciferase reporter gene expression (reported as ID_50_ and IC_50_ for serum and mAbs, respectively). Assays were performed with the vaccine strain (BG505/T332N GT1.1), the vaccine strain with the N276 glycan restored (BG505/T332N GT1.1 N276) the parental BG505/T332N virus, a heterologous clade C virus designed to detect early precursors of VRC01 lineage CD4bs antibodies (426c.N276D.N460D.N463D, also refered to as 426c.TM) (45) and a heterologous clade C virus (CH505TF.gly4) designed to detect early precursors of CH103 lineage CD4bs antibodies (75). CD4bs specificity of neutralizing activity was assessed with VRC01 knock out mutant viruses BG505/T332N_GT1.1 N279K and 426c.TM D279K. All viruses were produced in 293T cells except for 426c.TM and 426c.TM D279K, which were produced in 293T/GnT1-cells to enrich for Man5 glycoforms of N-linked glycans that otherwise are processed into large complex-type glycans (45).

#### Statistical analysis of serum binding and neutralization

Serum samples were declared to have positive binding antibody responses to a single antigen at post-enrollment visits if they met the following three criteria: i) background- and blank-subtracted (net) MFI values at the 1:50 dilution were greater than the maximum of 100 or the 95th percentile of baseline samples (by antigen); ii) net MFI values were greater than three times the baseline net MFI; iii) background-adjusted MFI values were greater than three times the baseline background-adjusted MFI values. To assess differential binding to the GT1.1 CD4bs, the net MFI for GT1.1 had to meet the 3 criteria: be ≥ 250; and the ratio of the net MFI and the background-subtracted MFI for GT1.1 to the GT1.1 CD4bs KO had to be ≥ 2.5 at a dilution where both values were < 22,000. The magnitude of the response, or area under the titration curve (AUC), was calculated over the dilution series for each sample using the trapezoidal integration method, with net MFI values truncated at a minimum of zero and a maximum of at 22,000 prior to AUC calculation. The antigen-specific response call was applied to the net MFI values, and the differential binding response calls were made at the first dilution where experimental and reference antigen net MFI were less than 22,000. Group comparisons of binding antibody titers between the low and high dose groups were carried out based on data from the entire cohort (*i.e*., regardless of response calls) using the two-sided Wilcoxon rank-sum test.

Serum samples were declared to have positive binding antibody responses to a single antigen at post-enrollment visits if they met the following three criteria: i) background- and blank-subtracted (net) MFI values at the 1:50 dilution were greater than the maximum of 100 or the 95th percentile of baseline samples (by antigen); ii) net MFI values were greater than three times the baseline net MFI; iii) background-adjusted MFI values were greater than three times the baseline background-adjusted MFI values. To assess differential binding to the GT1.1 CD4bs, the net MFI for GT1.1 had to meet the 3 criteria, be ≥ 250, and the ratio of the net MFI and the background-subtracted MFI for GT1.1 to the GT1.1 CD4bs KO had to be ≥ 2.5 at a dilution where both values were < 22,000 and set to 100 if < 100. The magnitude of the response, or area under the titration curve (AUC), was calculated over the dilution series for each sample using the trapezoidal integration method, with net MFI values truncated at a minimum of zero and a maximum of at 22,000 prior to AUC calculation. The ratio of net MFI for GT1.1 to the GT1.1 CD4bs KO at the dilution where both values were less than < 22,000 was referred to as the differential binding ratio. The antigen-specific response call was applied to the AUC values, and the differential binding response call was applied to the ratio. Group comparisons of binding antibody titers between the low and high dose groups were carried out based on data from the entire cohort (*i.e*., regardless of response calls) using the two-sided Wilcoxon rank-sum test.

#### Electron microscopy-based polyclonal epitope mapping (EMPEM)

To prepare polyclonal samples, previously published methods were generally followed (76). Briefly, IgG was isolated from heat-inactivated serum samples using a Cytiva ALIAS autosampler and Cytiva AKTA Pure system by capturing over pre-packed HiTrap MAbSelect PrismA protein A columns (Cytiva). Purified polyclonal IgG was digested using papain (to generate polyclonal Fab, and Fc and undigested IgG was removed by incubating with CaptureSelect IgG-Fc multispecies affinity resin (Thermo Fisher Scientific). 1 mg of each polyclonal Fab sample was complexed with 15 μg of GT1.1 SOSIP-v4.1 and incubated overnight at 20 °C. Complexes were purified over a Superdex 200 Increase size exclusion column (Cytiva) using a Cytiva AKTA Pure system and Tris-buffered saline (50 mM Tris pH 7.4, 150 mM NaCl) as running buffer.

To prepare EM grids, polyclonal complexes were diluted to ~0.03 mg/mL using Tris-buffered saline. A 3 μL drop of sample was applied to a glow-discharged carbon-coated copper mesh grid for 10 s, blotted with Whatman #1 filter paper, and a 3 μL drop of 2% (w/v) uranyl formate solution was applied for 45–60 s, followed by blotting. Grids were clipped, loaded into a Thermo Fisher Scientific (TFS) Autoloader cassette, and imaged using a TFS Glacios microscope operating at 200 keV, equipped with a TFS Falcon IV detector, and 73,000x magnification (resulting in a 1.89 Å pixel size). Data was automated using EPU Multigrid (TFS) and processed using Relion 4.0 (77). Following three rounds of 2D classification, particles from classes corresponding to immune complexes were subjected to 3D refinement with C3 symmetry and a 40 Å low-pass filtered map of HIV Env ectodomain as the initial model. The initial model is based on PDB coordinates 6V0R, converted in a map using the molmap feature in UCSF ChimeraX (78). Following 3D refinement, C3 symmetry expansion was applied to the particles and 7 separate focused 3D classification “skip align” jobs were run (K=10), each with a 40 Å diameter spherical mask over key HIV Env epitopes. The names of the epitopes and reference structures used for orienting the masks are: 1) gp41-base (PDB 6X9R), 2) gp41-GH (PDB 7L8U), 3) gp41-FP (PDB 7L8T), 4) gp120-GH (PDB 7L8B), 5) C3V5 (PDB 7L86), 6) CD4bs and gp120 interface (PDB 7L8X), and 7) V1V2V3 (PDB 7L8E). For each epitope 3D classification, classes with visible Fab density were selected and subjected to 3D refinement, 2D classification, and a second round of 3D classification. If the 3D refinement resulted in partial Fab density relative to the Env trimer, classes were selected from the subsequent round of 3D classification and this was repeated until the reconstruction improved or no change was noted. Final reconstructions were visually inspected and assigned the correct epitope label. Visualization and image generation was performed using UCSF ChimeraX. Representative EM maps have been deposited to the Electron Microscopy Data Bank (EMDB).

#### B cell analytics overview

The primary aim of B cell analyses was to assess whether prespecified B cell subsets (*e.g*., IgG memory B cells) responded to the GT1.1 gp140 vaccine and to further subset those B cells based on whether they targeted the CD4bs-epitope region, the apex-epitope region, or another off-target site on GT1.1. We accomplished this using a combination of four proteins: GT1.1, GT1.1 CD4bs KO (D368R/N279A), GT1.1 apex KO (N156Q/N160Q/D167G/K168E/K169V) and GT1.1 super KO (both sets of KO mutations). We labelled these proteins to fluorochromes and fitted them into a flow cytometry B cell phenotyping panel. These four fluorescently-labelled proteins are together referred to as the GT1.1 probe-set.

Using flow cytometry, we detected B cells responding to GT1.1 (antigen+ B cells) and further distinguished their epitope specificities based on differential staining to the respective knockout proteins. Those B cells that were able to bind to GT1.1 and GT1.1 apex KO, but not to GT1.1 super KO were identified as CD4bs-specific B cells. Those B cells that were able to bind to GT1.1 and GT1.1 CD4bs KO, but not to GT1.1 apex KO and GT1.1 super KO were identified as apex-specific B cells. B cells that were not classified as CD4bs-specific or apex-specific B cells but were GT1.1-specific were categorized as ‘epitope-unclassified’ B cells. We identified B cells with these different specificities within IgD+ naive B cells and IgG+ memory B cells in PBMCs; we generally refer to these specific subpopulations when discussing “naive” or “memory” B cells. We also sorted and sequenced CD4bs-specific and apex-specific B cells, from among the IgG+ memory and/or IgD+ naïve B cell compartment, depending on the treatment group and timepoint. The total numbers of PBMCs processed from the different groups and timepoints are indicated in table S7. Methods were established and optimized and all samples were processed at the Vaccine Research Center, NIH.

#### Probe preparation

Probes were prepared at 1:4 molar ratio of streptavidin-fluorochrome to trimeric GT1.1, or GT1.1 apex KO, or GT1.1 CD4bs KO, or GT1.1 super KO. The total volume of protein, streptavidin-fluorochrome and 1X PBS were added to a microfuge tube, mixed and incubated in the dark at 4 °C for 60 min. Probes were made and stored at 4 °C and used within 24 h.

#### Probe quality check

Bead assays were performed on the day samples were processed to confirm functionality of probes by flow cytometry. Two tests were set up for each probe (one experimental and one control) as follows. Anti-mouse Ig-kappa beads were mixed with an equal volume of R10 media in polystyrene FACS tubes such that there was one tube per test. 1 μg of mouse anti-human IgG was added to each tube and incubated at 4 °C for 15 min. Beads were washed with R10 and resuspended in 100 μL of R10. To the experimental tubes, 1 μg of linker antibody was added. Linker antibodies were VRC01-class (CD4bs-specific), PGT145 (apex-specific), PGT151 (gp120–41 interface-specific) or 3H2 or 5H3 (base-specific). Linker antibodies were not added to control tubes. After incubation at 4 °C for 15 min, beads were washed and resuspended in 100 μL of R10. Probes were added and the tubes incubated at 4 °C for 15 min. Beads were washed with R10 and resuspended in 200 μL of R10 for collection.

#### Sample batching schemes

PBMC samples were batched such that longitudinal samples from one donor were run together for acquisition and sorting.

#### Overview of sample processing workflow

PBMC samples were enriched for B cells and stained with a B cell phenotyping panel fitted with probes. B cell enrichment was incorporated into the PBMC sample-processing workflow to eliminate non-specific binding and T cell binding to probes.

#### B cell enrichment

Samples were enriched for B cells based on negative-selection using the StemCell pan-B cell enrichment kit using the manufacturer’s instructions. Briefly, cryopreserved samples were thawed using ThawSTAR cryovial adaptors into warm R10 containing benzonase. Supernatants were decanted and cells were washed with EasySep buffer. After the wash, cells were resuspended in 1–2 mL of EasySep buffer for B cell enrichment according to the manufacturer’s instructions. Volumes of enrichment cocktail and magnetic beads added per sample were adjusted based on cell numbers being processed.

#### Flow cytometry staining with phenotyping markers and probes

Cells were incubated for 2 min with viability blue dye at RT to stain dead cells. Following incubation, cells were stained with a panel of fluorescently-labelled markers along with GT1.1 super KO probe for 30 min in the dark at 4 °C, washed once with R10, and stained with remaining probes for another 30 min in the dark at 4 °C. After incubation, cells were washed twice with R10, and resuspended in R10 for collection.

#### Flow cytometry gating

Standardized gating templates were designed using the BD Diva software. A template was created for analysis of PBMC samples. These templates were replicated for each experiment to ensure consistent gating of samples. A schematic of the gating strategy is shown in fig. S8.

#### FACS

CD4bs-specific B cells and apex-specific B cells identified using the GT1.1 probe-set were single-cell sorted into 96-well plates containing 1% basal medium eagle (BME) in TCL buffer (Qiagen, 72251) and stored at −80 °C until ready for B cell immunoglobulin sequencing using the SMARTseq protocol. Raw flow cytometry files were imported into FlowJo software (v10.2) and were gated as shown in fig. S8.

### B cell receptor sequencing

#### Sample selection

We selected a subset of samples for B cell receptor sequencing. All week 26 (post-third vaccination) samples from vaccine recipients were sequenced; week 10 (post-second vaccination) samples from high dose recipients and two low dose recipients were sequenced based on their low GT1.1-specific memory B cell frequency at week 26; week −4 (pre-vaccination) samples were sequenced from low dose recipients only.

#### Cell lysis and reverse transcription

A modified SMARTseq-v4 protocol was used to synthesize variable heavy and light chains from each cell by template switching mechanism at 5’ end of RNA as described previously (79) and detailed below. Frozen plates were removed from −80 °C and centrifuged at 800 g briefly and then thawed on wet ice for 5 min. A stock solution containing 10X Lysis buffer (Takara Bio, 635013) with Recombinant RNase Inhibitor (Takara Bio, 2313B) at a 19:1 ratio was prepared and 0.6 μL of the stock solution was dispensed to every well via automatic liquid handler Firefly^®^ (SPT Labtech). After gentle vortexing and brief centrifuging, the plates were incubated at RT for 5 min for cell lysis. The lysed cells’ RNA was purified by Agencourt^®^ RNAClean^®^ XP (Beckman Coulter, A63987) at a ratio of 2.2 times the cell suspension volume. Each single cell’s RNA was eluted with 1.25 μL of TSoligo2_polydT (10 μM) and 2 U of Recombinant RNase Inhibitor in a total volume of 10 μL nuclease-free water (Invitrogen, AM9939). After the beads with RNA were thoroughly resuspended in the elution solution by vortexing, the plates were incubated in a preheated, hot-lid thermal cycler at 72 °C for 3 min. Once completed, the plates were immediately placed on wet ice for 2 min.

#### cDNA synthesis

The first strand cDNA synthesis reaction was then initiated with the RNA template using the Smartseq^®^ v4 Ultra Low Input RNA kit (Takara Bio). Every reaction consisted of 1X First-Strand Buffer, A-tag TSO (50 μM), 20U of Recombinant RNase Inhibitor, 1mM dNTP mix, 2.5mM of DTT and 100U SMARTScribe Transcriptase (Takara Bio, 639538) in total volume of 20 μL. The cDNA synthesis’s reverse thermal cycling parameters were 42 °C for 90 min, 70 °C for 10 min, 4 °C hold.

#### cDNA amplification

A cDNA amplification reaction mix was then made containing 1 U SeqAmp DNA Polymerase (Takara Bio; 628509), 1x SeqAmp PCR Buffer (Takara Bio), 10 μM TSO_FWD, 10 μM TSOligo2 and ultra-pure water to a total volume of 24 μL and added directly to the 20 μL single cell first strand cDNA. After 22 cycles of PCR reaction, the amplified cDNA plate was purified using 26.4 μL AmpureXP beads (Beckman coulter Cat: A63881) added to 44 μL of amplified cDNA. The purified product was eluted into 20 μL of RNase-free water. Purified and amplified cDNA was then used for heavy and light immunoglobulin transcript amplification.

#### Immunoglobulin gene amplification

Heavy and light immunoglobulin (Ig) chains were amplified in one PCR reaction. The reaction mix consisted of 20 μM immunoglobulin gamma (IgG), 5 μM immunoglobulin kappa (IgK), 5 μM immunoglobulin lambda (IgL), and 20 μM universal TSO_FWD primer with 0.625 U SeqAmp DNA Polymerase and 1 U SeqAmp PCR Buffer. All sequences of the primers are listed in table S6. After dispensing the 15 μL PCR reaction mix to new 96-well plates, 5 μL of purified cDNA template was added to corresponding wells. Thermal cycling parameters were 95 °C for 1 min and 15 cycles of 98 °C for 10 s, 65 °C for 30 s, and 68 °C for 90 s, followed by a final extension of 72 °C for 3 min. Once complete, the Ig PCR products were purified using AmpureXP beads (Beckman Coulter, A63881) in a ratio of 0.8X beads to PCR products, and the purified product was eluted with 20 μL of RNase-free water. The purified Ig PCR product was quantified in each well using the QuantIT dsDNA high sensitivity kit (Thermo Fisher Scientific, Q33120) and normalized to 0.2 ng/μL for optimal Next Generation Sequencing (NGS) library construction using the Illumina Nextera XT kit (Illumina, FC1-31-1096).

#### Library construction and Illumina sequencing

In new 96-well plates, 1.25 μL of 0.2 ng/μL purified PCR products were mixed with 2.5 μL of Tagmentation buffer and 1.25 μL of the Amplicon Tagmentation enzyme provided in the Nextera kit. The plates were incubated at 55 °C for 10 min with a preheated lid thermocycler and then cooled to 4 °C immediately. Following the incubation, 1.25 μL of neutralization buffer was added to the wells and incubated at room temperature for 5 min. Each plate was indexed with 1.25 μL of a 10 mM unique 10-base pair Mi7 index, and each well of the plate was indexed with 1.25 μL of a unique 10 base pair Mi5 index at a concentration of 10 mM, along with 3.75 μL of the NPM reaction mix from the Nextera XT kit. The library construction plates were placed in the thermocycler for indexing and amplification. The thermocycler parameters used were 72 °C for 3 min, 95 °C for 30 s, and 12 cycles of 95 °C for 10 s, 55 °C for 30 s, and 72 °C for 30 s, followed by a final extension of 72 °C for 5 min. 4 μL of each indexed single B cell receptor libraries were pooled together for AmpureXP bead purification at 0.8 U concentration and eluted into 50 μL of RNAase-free water for each plate. The final concentration of the pooled and purified library was determined using Qubit^™^ dsDNA Quantification Assay Kit, High Sensitivity (HS) (Invitrogen, Cat: Q32854) and Bioanalyzer High Sensitivity DNA (Agilent, 5067–4626) and diluted to 2 nM with RNase-free water for sequencing. Indexed pooled libraries from up to 12 plates were diluted to 720 pM by Nextseq 1000/2000 RSB with Tween 20 (Illumina, 20050639) and then paired-end sequenced together with 0.04 μM PhiX Control V3 (Illumina, FC-110–3001) via Illumina Nextseq1000 on P1 300 cycles kits (Illumina 20046811) with the sequencing read lengths set to 151bp for read 1, 10bp for index 1, 10bp for index 2 and 151bp for read 2. The demultiplexed fastq.gz files were retrieved from the on-board Dragon BCL Convert for Illumina Nextseq 1000 and transferred to the Vaccine Immunology Statistical Center (VISC) for data repository and analysis.

#### BCR sequence assembly, alignment, and annotation

The BALDRlite pipeline, created and maintained at VISC, was used to assemble paired heavy and light chain variable-region BCR sequences for each well. This pipeline makes use of components of the BALDR package (80), which in turn makes use of the Trinity assembler (81). BALDR’s Perl wrapper was rewritten to be compatible with up-to-date versions of its components, and the Nextflow workflow manager (82) was incorporated to accelerate data processing and improve reproducibility. Candidate sequences were filtered to include only productive assemblies, where sequence alignment includes both the first V gene codon that encodes the mature polypeptide and the last complete codon of the J gene. At most one (top-ranked) candidate transcript was down-selected per chain type (heavy or light) and per well/cell. V(D)J germline allele sequences were retrieved from the curated Open Germline Receptor Database (version released on January 11, 2024) (83). These germline allele sequences formed the basis of alignment indices for Bowtie2 and IgBLAST (84, 85), which were used to filter reads for relevance and to rank assembled chain transcript candidates for each cell. The IGHV1–2 allele sequences were restricted for each study participant to those identified by IgDiscover as belonging to the participant’s genotype (see section below). IgBLAST germline gene and CDR3 sequence annotations were used to annotate B cells as VRC01-, IOMA-, 8ANC131-, and V2-apex-class. The R package alakazam was used to calculate the net charge of the CDR3 (86, 87).

#### Clonotyping

Clonotypes were computed to identify sequenced B cells that were either plausibly clonally related or expressed BCRs with similar nucleotide sequences. Clonotyping was performed using the single-linkage hierarchical clustering algorithm, as implemented in the R package Scoper from Immcantation (88). Paired heavy and light BCR sequences were assigned to the same clonotype if they had identical heavy and light chain V and J genes, identical CDR3 lengths, and their joint CDR3-sequence Hamming distance (normalized by the total length of the heavy and light chain CDR3 sequences) was ≤0.15.

#### Missing data and quality control for B cell assays

The B cell assays were conducted at baseline (pre-vaccination), week 10 (two weeks after second vaccination), and week 26 (two weeks after third vaccination). The B cell phenotyping (flow cytometry) assays were conducted for all participants with samples available at each of the three time points. The BCR sequencing assays were conducted at baseline and week 26 for the low-dose group, and at week 10 and week 26 for the high dose group. Two participants from the low dose group had low sample quality/viability for the week 26 sample; hence, BCR sequencing was also run on a week 10 sample for these participants. In addition to three study participants who missed vaccinations, one high-dose vaccine recipient did not undergo leukapheresis at week 10, and one high-dose vaccine recipient had insufficient sample collected at week 26. As a result, flow cytometry and BCR sequencing data is absent for these participants at these time points.

In addition, no BCR sequences were successfully recovered from any of the placebo recipients at week 26, due to the small numbers of antigen-specific and epitope-specific B cells that could be sorted for sequencing from these samples. Similarly, one placebo recipient and three low dose vaccine recipients in group 1 had no BCR sequences successfully recovered from their baseline samples, and all four placebo recipients in group 2 had no BCR sequences successfully recovered from their week 10 samples. Wells were also scored for possibilities of positive control, well-to-well, and carryover contamination, and questionable sequences were excluded from the dataset. A total of 212 sequences were excluded from the high dose group data, and a total of 122 sequences were excluded from the low dose group data. A conservative approach was taken to filtering the data when indications of possible contamination or questionable quality were present, in order to increase our confidence in the quality of the analysis. The B cells (wells) filtered out of the dataset may or may not represent instances of actual contamination.

#### Statistical analysis of B cell flow cytometry and BCR sequencing data

B-cell frequencies were calculated as the ratios of cell counts from flow cytometry or BCR sequencing data. Frequencies such as “VRC01-class among IgG memory B cells” were approximated by the product of two ratios, with the first one coming from flow cytometry and the second one coming from BCR sequencing: “(number of CD4bs-specific IgG memory B cells) / (number of IgG memory B cells)” times “(number of VRC01-class BCR sequences) / (number of sequenced CD4bs-specific IgG memory B cells)”. This approach was necessary because not every CD4bs-specific IgG memory B cell was successfully sequenced.

For flow cytometry endpoints, positive response calls were assessed as follows. A certain amount of variation in the values of these endpoints was expected, both across participants and across time points within the same participant, but the amount of such variation was not known *a priori*. Response calls for post-baseline visits were therefore carried out using a kernel density estimator (KDE) fit to the endpoint measurements from all participants at baseline (*i.e*., pre-vaccination) and placebo recipients at post-baseline time points. The R package ks was used for computation of the kernel density estimate, with a Gaussian kernel and the bandwidth of the estimator selected using the univariate plug-n selector of Wand and Jones (89). The quantile Q_α_ of order 1-α of the null distribution was deduced from the density estimate, with α representing the probability that an observation from a true non-responder would exceed Q_α_. We set α=0.001, and a positive response call was made if the endpoint was observed to be strictly greater than the estimate of Q_α_.

For endpoints based at least partially on BCR sequencing (*e.g*., VRC01-class among IgG memory B cells), responses were defined as “detectable” if any B cells (*i.e*., >0) in the relevant category (*e.g*., VRC01-class) were observed. We note that limitations in sampling depth (*i.e*., the number of BCR sequences obtained) could result in a non-detectable response, even if the given participant was a true responder.

Formal statistical comparisons were conducted to assess differences between study visits (within each treatment group) and between treatment groups (at each study visit). To assess if the rates of positive/detectable responses differed between two treatment groups at a given study visit, a two-sided Barnard’s exact test was used. To assess if the rates of positive/detectable responses differed between two study visits within a given treatment group, a two-sided McNemar’s test was used to account for paired data. To assess if the values of continuous endpoints (*e.g*., B-cell frequencies, percent mutation) differed between two treatment groups at a given study visit, a two-sided Wilcoxon rank-sum test was used. To assess if the values of continuous endpoints (*e.g*., B-cell frequencies, percent mutation) differed between two study visits within a given treatment group, a two-sided Wilcoxon rank-sum signed-rank test was used to account for paired data. Observations (paired if relevant to a comparison between two study visits) were required from at least 3 participants per treatment group in order to run any statistical test. The reported p-values were not adjusted for multiple comparisons.

For the BCR sequencing analysis, a formalized data set from VISC was used (dated December 11^th^, 2024). All analyses, except the analysis on IgD naive B cells (fig. S12), was performed with the above data set but filtered on IgG memory B cells.

#### Comparisons to Observed Antibody Space (OAS) data as a control

In control data sets that use the Observed Antibody Space data set, we restricted to human, non-vaccinated, no disease state data.

#### Individualized IGH genotyping

All individuals in the trial were individually genotyped for their IGH V, D and J allele content, as described previously (90). In brief, total RNA was extracted from isolated PBMC cells, with 200 ng used as template to create IgM libraries that contained unique molecular identifiers (UMIs). The libraries were indexed and sequenced on the Illumina MiSeq instrument using the 2 × 300 cycle V3 kit (Illumina). The resultant IgM libraries were analyzed with the germline inference tool IgDiscover (31) and the genotyping tool *corecount* (91) to produce individual V, D and J genotypes for each case.

### mAb analysis

#### Selection of expressed mAbs

The selection of BCRs that were expressed as mAbs is detailed in fig. S25. Briefly, to enable rapid assessment of binding capacities of VRC01-class mAbs, a preliminary data set was shared with 729 BCR sequences from high dose recipients (weeks 10 and 26). From this preliminary data set, we selected BCRs with intact frameworks and unique sequences as many were identical due to the large degree of clonal expansion observed in the study (n = 481). We then prioritized B cell lineages present at both weeks 10 and 26 to enable longitudinal assessment of affinity (n = 301). From these lineages, we manually excluded 53 BCR sequences that were similar to other sequences within their B cell lineage. Thus, we arrived at 248 BCR sequences that were not specifically selected for any particular sequence characteristic. Next, we added small numbers of sequences based on i) a unique CDRL1 deletion that was not yet included; ii) highly mutated BCRs that had at least 12 mutations (aa) in their IGHV gene of which 9 were VRC01-class; iii) several sequences that were suspected to be generated from atypical IGHV1–2 alleles, prior to generating personalized immunoglobulin gene databases with IgDiscover (fig. S25). Due to our conservative approach in removing data when there were indications of possible contamination or questionable quality, 276/279 (99%) of selected BCR sequences from the preliminary data set were identical to the final data and were thus selected for subsequent analyses.

#### Enzyme-linked immunosorbent assay (ELISA)

His-tag-presenting proteins were produced in HEK293F cells, purified with PGT151 as previously described (24), and subsequently used in His-tag Capture ELISAs. Purified SOSIP trimers were diluted to 2 μg/mL in casein/TBS blocking buffer (Thermo Scientific, Cat. No.: 37583) and immobilized for 2 h at RT on Ni-NTA 96-well plates (Qiagen). Unbound protein was washed away with Tris-buffered saline (TBS), and serially diluted mAbs at 1 μg/mL, 10 μg/mL, 50 μg/mL, or 100 μg/mL were added and allowed to bind for 2 h at RT, after which they were washed away with TBS. Anti-Human IgG (H+L) Antibody, peroxidase-labeled (Seracare, Cat. No.: 5220–0330), was added for 60 min at a 1:3,000 dilution (final concentration 0.33 μg/mL) in Casein/TBS, followed by five washes with TBS/0.05% Tween-20. The plate was then developed with 0.1 M NaAc, 0.1 M citric acid, 1% TMB (Thermo Fisher, Cat. No.: N301), and 0.01% hydrogen peroxide (H_2_O_2_). Development was stopped after 5 min with 0.8 M sulfuric acid (H_2_SO_4_) and the absorbance was measured at 450 nm. mAbs were deemed positive for binding to a particular trimer when the OD_450_ value at the highest concentration was ≥3-fold above background. A subset of mAbs showed positive binding per this metric but did not have sufficient affinity for an EC_50_ to be calculated accurately.

#### Pseudovirus neutralization assay (Amsterdam UMC)

The neutralization capacity of mAbs was assessed using the TZM-bl neutralization assay, as previously described (24, 74). Briefly, TZM-bl cells, which express CD4, CCR5 and CXCR4, were cultured in Dulbecco’s Modified Eagle Medium (DMEM) supplemented with 10% FCS, penicillin (100 U/mL) and streptomycin (100 μg/mL). On day 0, cells were seeded into 96-well plates and incubated overnight. The next day the cells were 70–80% confluent. On day 1, monoclonal antibody dilutions were prepared in triplicate starting at four times the intended start concentration and threefold serial dilutions were performed. The antibodies were mixed 1:1 with pseudovirus and incubated at RT for 1 h. After incubation, the antibody-virus mix was added to the cells, which were treated with DEAE-dextran (40 μg/mL) and saquinavir (400 nM) at a 1:1 ratio. After a 72 h incubation period, the cells were lysed and the luciferase activity measured with the Bright-Glo Luciferase system (Promega). The inhibitory concentration (IC_50_) was determined as the antibody concentration that reduced infectivity by 50%, calculated by normalizing to the untreated virus-positive control.

#### Surface plasmon resonance (SPR)

Binding of mAbs to BG505 SOSIP, GT1.1, GT1.1 N276 trimers was analyzed by surface plasmon resonance (SPR) on a BIAcore T200 instrument. HBS-EP^+^ (0.01 M HEPES pH 7.4, 0.15 M NaCl, 3 mM EDTA, 0.05% v/v Surfactant P20) was used as running buffer and the experimental temperature was 25 °C throughout. Anti-histidine antibody was covalently coupled to a Series S CM3 sensor surface following standard amine-coupling protocol with the reagents of the His-Capture kit (Cytiva). The sensor surface was activated with EDC:NHS (1:1) for 10 min. Anti-histidine antibody (50 μg/mL in 10mM Na-acetate buffer, pH 4.5) was allowed to couple for 10–15 min to give an immobilization level of ~4.0 · 10^3^ RU. Remaining carboxyl groups were deactivated by a 5 min injection of ethanolamine. The immobilization was performed at a flow rate of 10 uL/min. mAb binding was analyzed by single cycle kinetics (SCK). The three trimers were immobilized at a flow rate of 5 uL/min in parallel flow cells, the remaining one serving as the no-trimer background control. Trimer capture levels, *R*_*L*_, were adjusted to give *R*_*max*_ ~ 15 RU for mAb binding. mAb was injected at five ascending concentrations adjusted to give incremental binding through the whole cycle. Each mAb was serially diluted (2-fold), the highest concentrations being 250 nM (12A01, 3G08, 4D01) or 500 nM (9C09 and 7A03). Association with escalating concentrations was monitored for 5 min and dissociation for 1 h. In case of extremely strong binders (12A01 and 7A03 *versus* GT1.1), the highest mAb concentration was reduced to 10 nM, and the dissociation time was extended to 2 h to get a significant measurement of *k*_*off*_. The maximum flow rate of 50 uL/min was used throughout to counteract mass-transport limitation (MTL). At the end of each cycle the sensor surface was regenerated by two 90 s pulses of glycine (10 mM, pH 1.5).

The binding data were modeled with Biacore T200 Evaluation Software v3.2.1 after zero-trimer and zero-mAb background subtractions. The CM3 chip, which has a short dextran matrix, and the low trimer-immobilization level were chosen to avoid bivalent binding, enabling us to determine the kinetics of the intrinsic, monovalent interaction of IgG. Dissociation curves were cut where they came within 5% from the baseline. The goodness of fit of the Langmuir and conformational-change models to the MAb-binding data was compared. In all cases of substantially inferior fits of the Langmuir models, in terms of *χ*^***2***^ or by inspection of curves and search for spurious reflective-index shifts, the fit of the conformational-change model was selected for further validation. All fits were evaluated for significance of the kinetic parameters (*T*_*kon*_ and *T*_*koff*_ > 100), spread of residuals (1–2 RU), and absence of MTL (*t*_*c*_ > 10^8^ and *T*_*tc*_< 10 or *t*_*c*_ */k*_*on*_ > 100 (excellent) or >10 (acceptable) (tables S12–S14). The conformational-change model was further validated by injection-time-variation tests (fig. S32). In the injection-time-variation test, the analyte was injected for 30 s or 600 s in the association phase. Near-saturating concentrations of MAbs (250 nM or 500 nM) were injected under conditions identical to those described above. Empirical sensorgram curves were normalized and overlayed to calculate the relative residual binding for 30s-vs. 600s-contact time after 600s of dissociation. Subsequent relative residual binding was expressed in %: the lower the percentage, the stronger the evidence for real conformational changes.

#### Structure determination using cryo-electron microscopy

3G08, 12A01, 9C09, 7A03 and 4D01 IgG were digested into Fabs using papain (Sigma Aldrich). 0.2 mg of GT1.1 SOSIP v4.1 (3G08, 12A01, 9C09) or BG505 SOSIP.664 (7A03, 4D01) was incubated overnight at room temperature with either 0.3 mg of one of the Fabs. Additionally, 0.3 mg of RM20A3 Fab (an NHP antibody specific to the base of BG505-derived trimers) was added to each complex to help with orientation sampling. The trimer-Fab complexes were then purified using a HiLoad 16/600 Superdex 200 pg (Cytiva) gel filtration column. The complexes were then concentrated to between 2–5 mg/mL for application onto cryoEM grids. Cryo grids were prepared using a Vitrobot Mark IV (Thermo Fisher). The temperature was set to 4 °C and humidity was maintained at 100% during the freezing process. The blotting force was set to 1 and wait time was set to 10 s. Blotting time was varied from 3 to 6 s. Detergents lauryl maltose neopentyl glycol (LMNG; Anatrace) or n-Dodecyl-β-D-Maltoside (DDM; Anatrace) at final concentrations of 0.005 or 0.06 mM, respectively, were used for freezing. UltrAufoil R 1.2/1.3 (gold, 300-mesh; Quantifoil Micro Tools GmbH) grids were used and glow discharged before sample application. 0.5 μL of detergent was mixed with 3.5 μL of samples and 3 μL of the mixture was immediately loaded onto the grid. Following blotting, the grids were plunge-frozen into liquid nitrogen-cooled liquid ethane.

Cryo grids were loaded into a Thermo Fisher Scientific Glacios electron microscope operating at 200 kV. Exposure magnification was set to 190,000x with a pixel size at the specimen plane of 0.718 Å. EPU software (Thermo Fisher) was used for automated data collection. Micrograph movie frames were motion and CTF corrected using cryoSPARC Live (92). The remaining data processing was performed in cryoSPARC. Particle picking was performed using blob picker initially followed by template picker. During extraction particles were downscaled to 1.034 Å/pixel to reduce box size and increase speed of downstream jobs. Multiple rounds of 2D classification and 3D ab-initio reconstruction were performed prior to 3D non-uniform refinement with global CTF refinement. Final data collection and processing stats are summarized in figure S33 and table S15. Model building was performed by docking homology models of trimer and Fab Fv in UCSF ChimeraX (78), manually building and refining in Coot 0.9.8 (93) and real space refinement using Phenix (94). Final models were validated using MolProbity and EMRinger in the Phenix suite, and statistics are summarized in table S15. All maps and models have been deposited to the Electron Microscopy Data Bank (EMDB) and Protein Data Bank (PDB), respectively with accession codes summarized in table S15.

#### Structure determination using crystallography

Expi293F cells (Thermo Fisher Scientific) were transiently transfected with plasmid DNA encoding Fab heavy and light chain pairs in a 1:1 ratio. Six days post-transfection, the culture supernatant was harvested and sterile-filtered through a 0.22 μm filter. Fabs were purified using CaptureSelect CH1-XL affinity matrix (Thermo Fisher Scientific), followed by size-exclusion chromatography (SEC) on a Superdex 75 16/600 column. eOD-GT8 was similarly expressed in Expi293S cells (Thermo Fisher Scientific), purified from the culture supernatant using a Ni-NTA affinity column with Ni Sepharose excel resin (Cytiva), and SEC on a Superdex 75 16/600 column. To crystallize unliganded Fabs, the protein sample was concentrated to 12 mg/mL in 20 mM Tris, 150 mM NaCl, pH 7.5. For complex formation, eOD-GT8 was mixed with an excess of C101 VRC01-class Fab and incubated at room temperature for 1 h. The complex was partially deglycosylated by digestion with endoH glycosidase at 37 °C for 30 min. The resulting mixture was purified by SEC on a Superdex 200 16/600 column and concentrated to 10 mg/mL. Crystallization screening was performed using a Rigaku CrystalMation robotic system with JCSG 1–4, and Top96 Cryo crystal screens at both 4 °C and 20 °C.

Crystals were cryoprotected by adding 15% ethylene glycol to the reservoir crystallization solution, then quickly plunged into liquid nitrogen for storage prior to data collection. Diffraction data were collected at the National Synchrotron Light Source II 17-ID-1 AMX. Data indexing, integration, and scaling were performed using HKL2000 (95). Structures were determined by molecular replacement in Phenix using Phaser (96). Model building and refinement were carried out using Coot and Phenix.refine, respectively (94, 97). The quality of the final structures was assessed with MolProbity (98), and additional validation was performed through the PDB validation server. Crystallization conditions, as well as data collection and refinement parameters for Fabs bound to eOD-GT8 and unliganded Fabs, are summarized in tables S16 and table S17, respectively.

## Supplementary Material

Supplementary Material (28-05-2025)

Supplementary Text

Figs. S1 to S37

Tables S1 to S17

## Data Availability

Pseudonymized data are not available for download, due to privacy restrictions under the General Data Protection Regulation (GDPR) by the European Union. Specific requests for access to pseudonymized trial data may be sent to IAVI (data@iavi.org) and access may be provided to a named individual in agreement with the rules and regulations of the European Data Protection Agency (EDPA) with a two-week response timeframe to requests. Participant-level immunogenicity data are available in an anonymized format through publicly accessible database IAVI Figshare, DOI:10.25382/iavi.28873295). All cryo-EM maps are available in the public repository Electron Microscopy Data Bank (EMDB) under accession codes EMD-48283, EMD-48286, EMD-48287, EMD-48290, and EMD-48291; cryo-EM atomic models are available in the public repository Protein Data Bank (PDB) under accession codes 9MIA, 9MI0, 9MIB, 9MII and 9MIH. X-ray crystallography data are available in the PDB under accession numbers 9MIC, 9MID, 9MIF, 9MJ3, 9MJC, 9MJD, 9MJI, 9MJ6, 9MK4. All other data are available in the main text or supplementary materials. Plasmids or proteins related to the immunogens, sort reagents, or antibodies employed in this study are available from RWS upon reasonable request (r.w.sanders@amsterdamumc.nl). In some cases, a material transfer agreement with Amsterdam UMC may be necessary.
