# Supplementary material for: Precise targeting of human HIV broadly neutralizing antibody precursors
Source: Science. Author manuscript; Available in PMC 2025 Aug 1. (PMC12313413; doi:10.1126/science.adv5572)
Supplement: Supplementary Material (28-05-2025) [file NIHMS2085699-supplement-Supplementary_Material__28-05-2025_.docx]

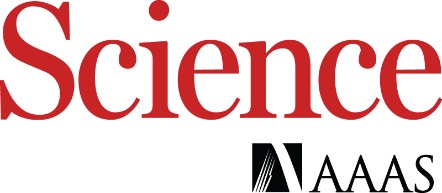


Supplementary Materials for

**Targeting human HIV broadly neutralizing antibody precursors**

Tom G. Caniels, Madhu Prabhakaran *et al.*

Corresponding authors: Marina Caskey, [mcaskey@rockefeller.edu](mailto:mcaskey@rockefeller.edu); Rogier W. Sanders, [r.w.sanders@amsterdamumc.nl](mailto:r.w.sanders@amsterdamumc.nl)

**The PDF file includes:**

Supplementary Text

Figs. S1 to S37

Tables S1 to S17

**Supplementary Text**

Detailed findings regarding safety and tolerability

As discussed in the Results section, the IPs were generally safe and well tolerated by study participants. No SAEs or pIMDs related to vaccinations were reported (table S2). One participant reported a grade 3 chest wall hematoma after undergoing elective bilateral mastectomies that required hospitalization; this SAE was considered not related to the study. There were no intercurrent HIV-1 infections but 4 participants developed vaccine-induced seropositivity (1 in low dose group, 3 in high dose group) that remained detectable in serum obtained 6 months after the third vaccination. No discontinuations for AEs related to the investigational product were reported.

Adverse events

37 of 39 vaccinees (95%) and all 8 placebo recipients (100%) reported an AE during study follow up. Most participants receiving GT1.1 experienced injection site pain (90%) or tenderness (95%), compared to 38% of placebo recipients (fig. S2 and table S3). Reactogenicity AEs typically lasted 1 to 3 days in most vaccine recipients and resolved within the reactogenicity window of 7 days post vaccination. One exception was a participant who reported grade 2 injection site edema that lasted for 8 days. Four vaccine recipients reported grade 3 local reactogenicity events that improved in severity or were fully resolved in 3 days. Systemic reactogenicity symptoms of grade 2 severity were reported by 51% of vaccine recipients and 38% of placebo recipients. Six vaccine recipients reported grade 3 systemic reactogenicity symptoms that resolved or decreased in severity in 1 to 2 days (fig. S2 and table S3). There was a similar distribution of type and severity of reactogenicity AEs between the GT1.1 dose groups (fig. S2). Local and systemic reactogenicity were also similar following each vaccination in respect of the number and grade of the events.

Twelve vaccinees (31%) and three placebo recipients (38%) reported unsolicited AEs that were considered at least possibly related to vaccination. All fifteen participants reported unsolicited related AEs of grade 1 severity, with the exception of one grade 2 fatigue reported by a high dose vaccine recipient (table S4). An overview of reactogenicity and possibly related non-reactogenicity AEs can be found in tables S2-S4. Unsolicited AEs were reported through 28 days after the final vaccination. Overall, the vaccine showed an acceptable safety and tolerability profile.


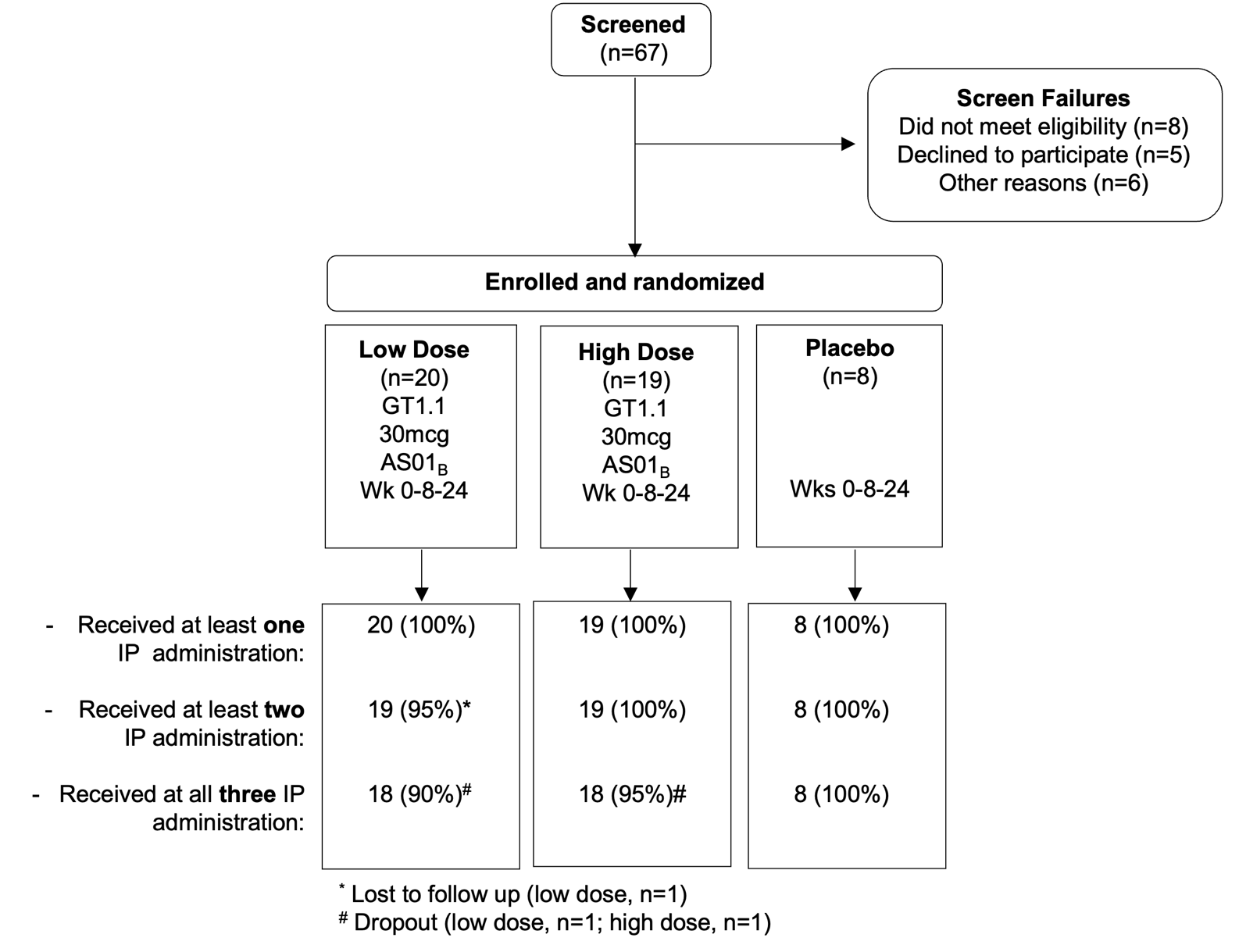


**Figure S1. Consort diagram.**


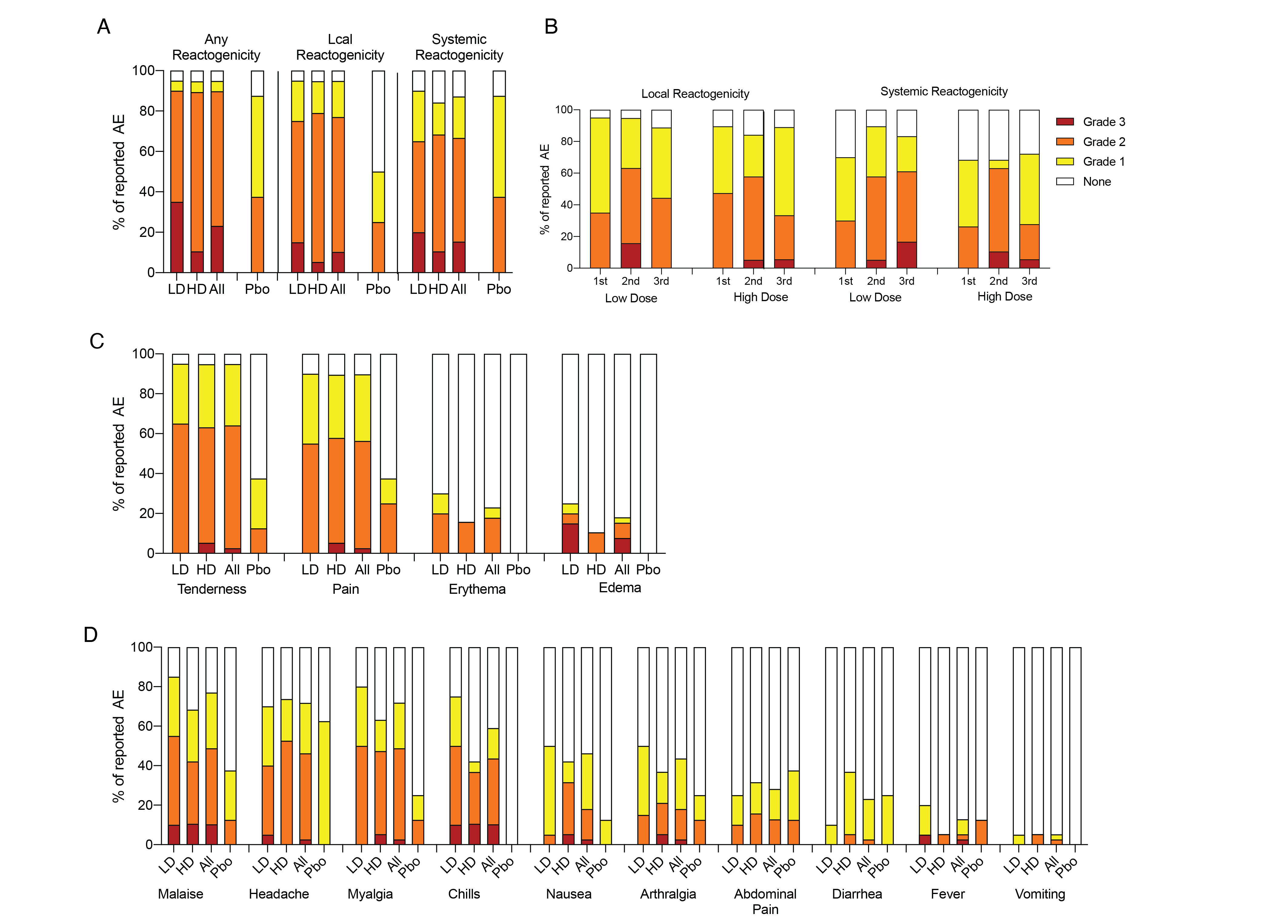


**Figure S2. Local and systemic reactogenicity events.** LD, low dose; HD, high dose; Pbo, placebo. **(A)** Adverse events (AEs) reported by group. **(B)** AEs by vaccine dose received. **(C)** Specific local AEs reported per group. **(D)** Specific systemic AEs reported per group.


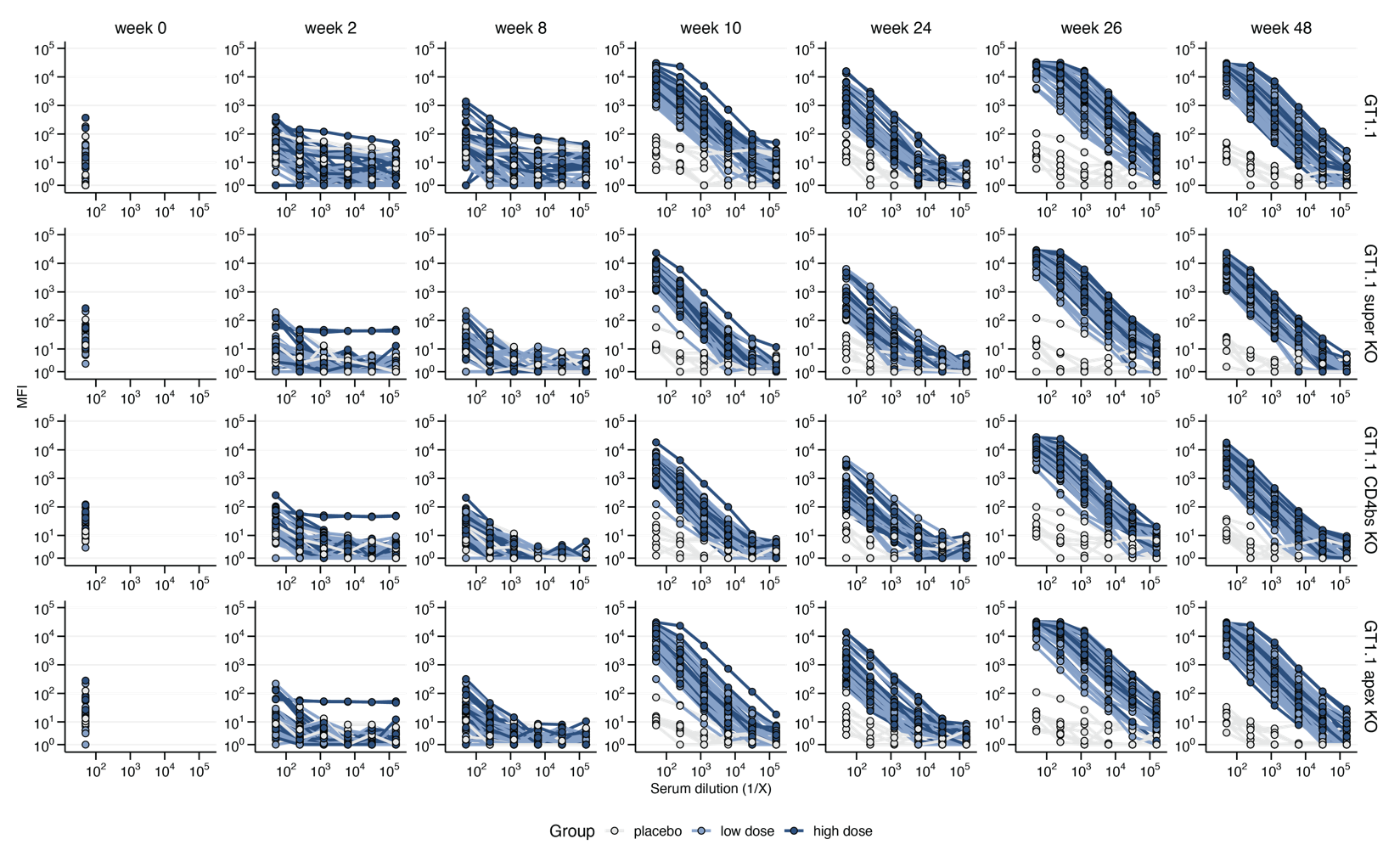


**Figure S3. Serum IgG analysis by binding antibody multiplex assay (BAMA) for GT1.1 and GT1.1 epitope knockouts.** Binding antibody (IgG) responses for placebo, low dose, and high dose groups expressed as mean fluorescence intensity (MFI) to GT1.1, GT1.1 super KO, GT1.1 CD4bs KO, and GT1.1 apex KO. Each line represents a single participant. Each symbol represents a different serum dilution. Each plot corresponds to an individual time point.


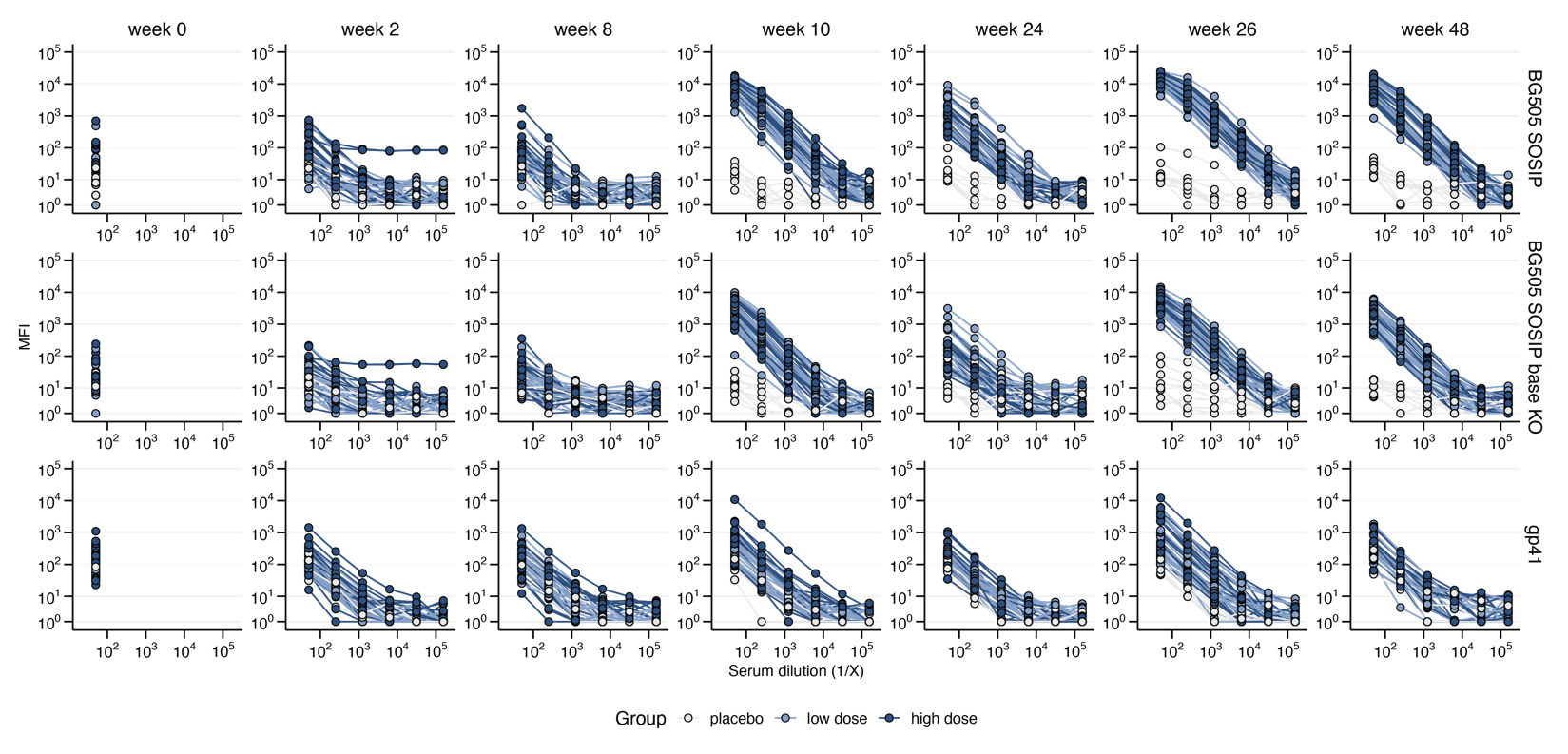


**Figure S4. Serum IgG analysis by binding antibody multiplex assay (BAMA) for BG505 SOSIP, BG505 SOSIP base knockout and gp41.** Binding antibody (IgG) responses for placebo, low dose, and high dose groups expressed as mean fluorescence intensity (MFI) to BG505 SOSIP, BG505 SOSIP base KO, and gp41. Each line represents a single participant. Each symbol represents a different serum dilution. Each plot corresponds to an individual time point.


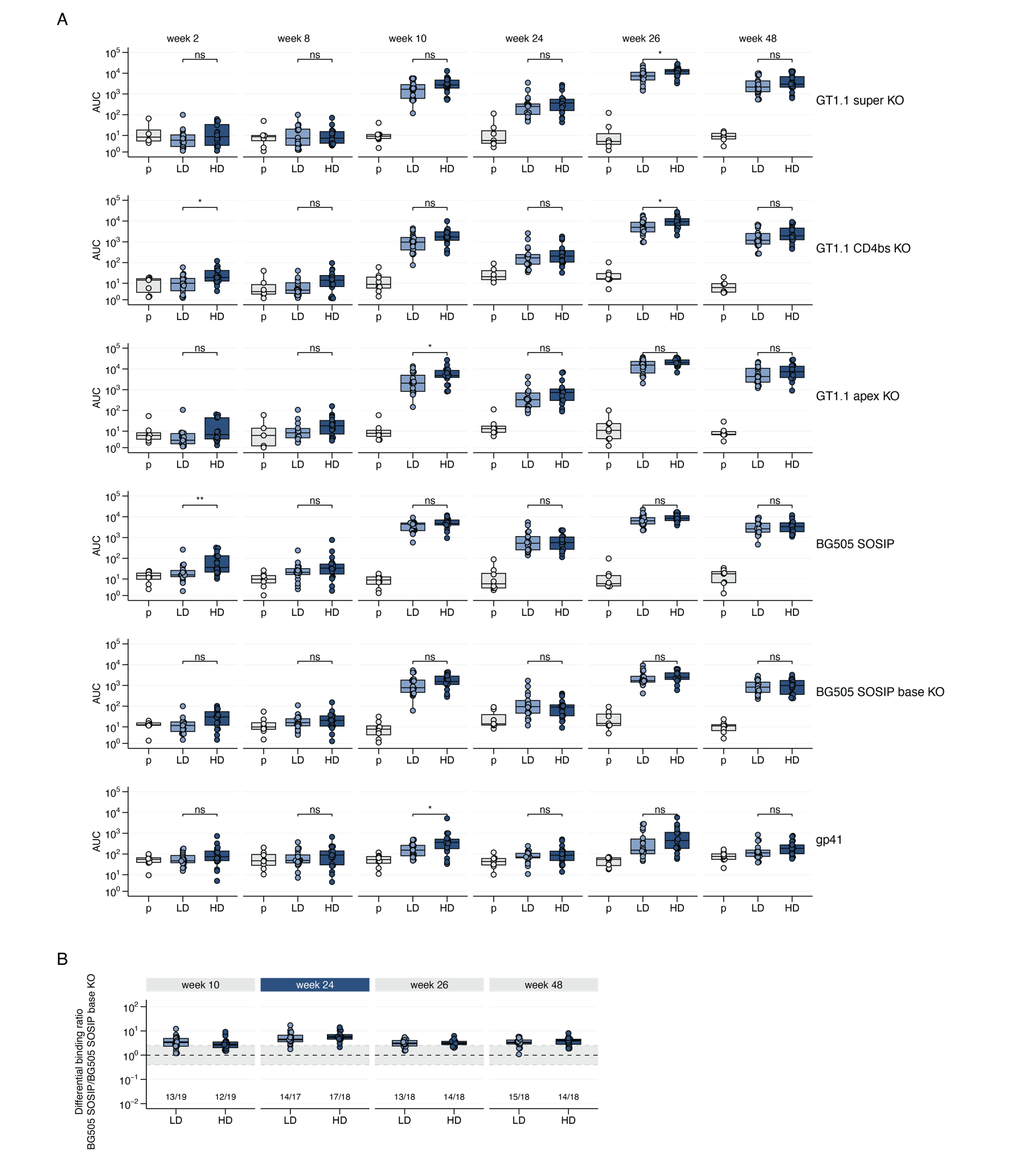


**Figure S5. Binding for all BAMA antigens and differential binding to BG505 SOSIP base KO. (A)** Binding antibody (IgG) responses expressed as the area under the curve (AUC) to GT1.1 super KO, GT1.1 CD4bs KO, GT1,1 apex KO, BG505 SOSIP, BG505 SOSIP base KO, and gp41. Each symbol represents a single participant at one time point. Wilcoxon test: ns, not significant; *, p < 0.05; **, p < 0.01. **(B)** Differential binding ratios (BG505 SOSIP/BG505 SOSIP base KO) based on net MFI values as in Fig. 1 (see Methods). A ratio of >2.5 (shaded area) was considered significantly positive. Each symbol represents a single participant at one time point. The median ratios of each group are displayed. In **(A)** and **(B)**, thick lines are medians and box plots show 25% and 75% quantiles.

**
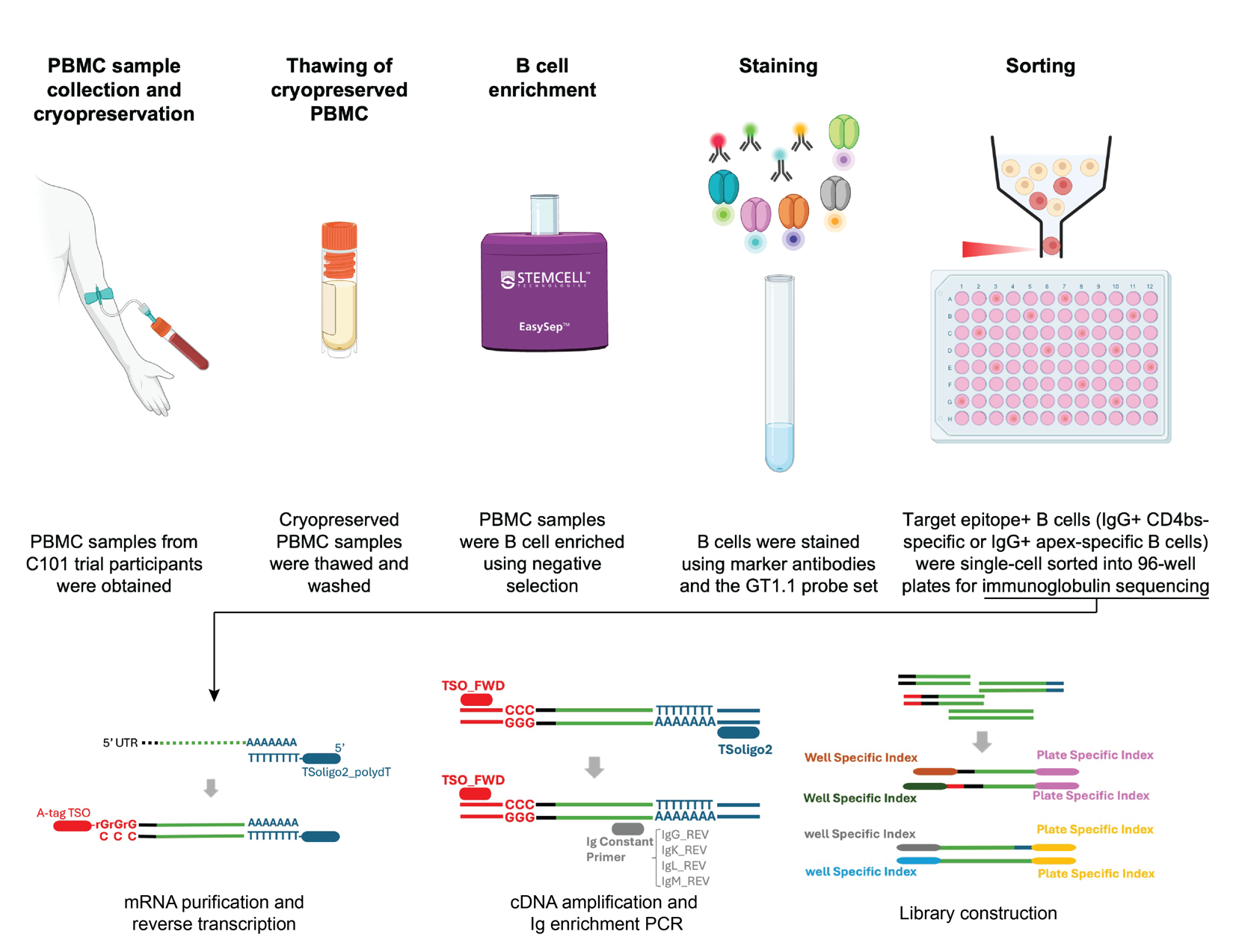
**

**Figure S6. Schematic overview of the B cell flow cytometry and sequencing pipeline.** Immunoglobulin sequencing: the single sorted cells were lysed, and mRNA purification, RT reaction, cDNA amplification, and Ig PCR amplification were carried out using robotic liquid handling systems. The amplified specific Ig PCR products were used to construct unique double-indexed fragments in a 96-well format. Finally, the constructed libraries were pooled and sequenced using the Illumina NextSeq 1000 (see Methods).

**
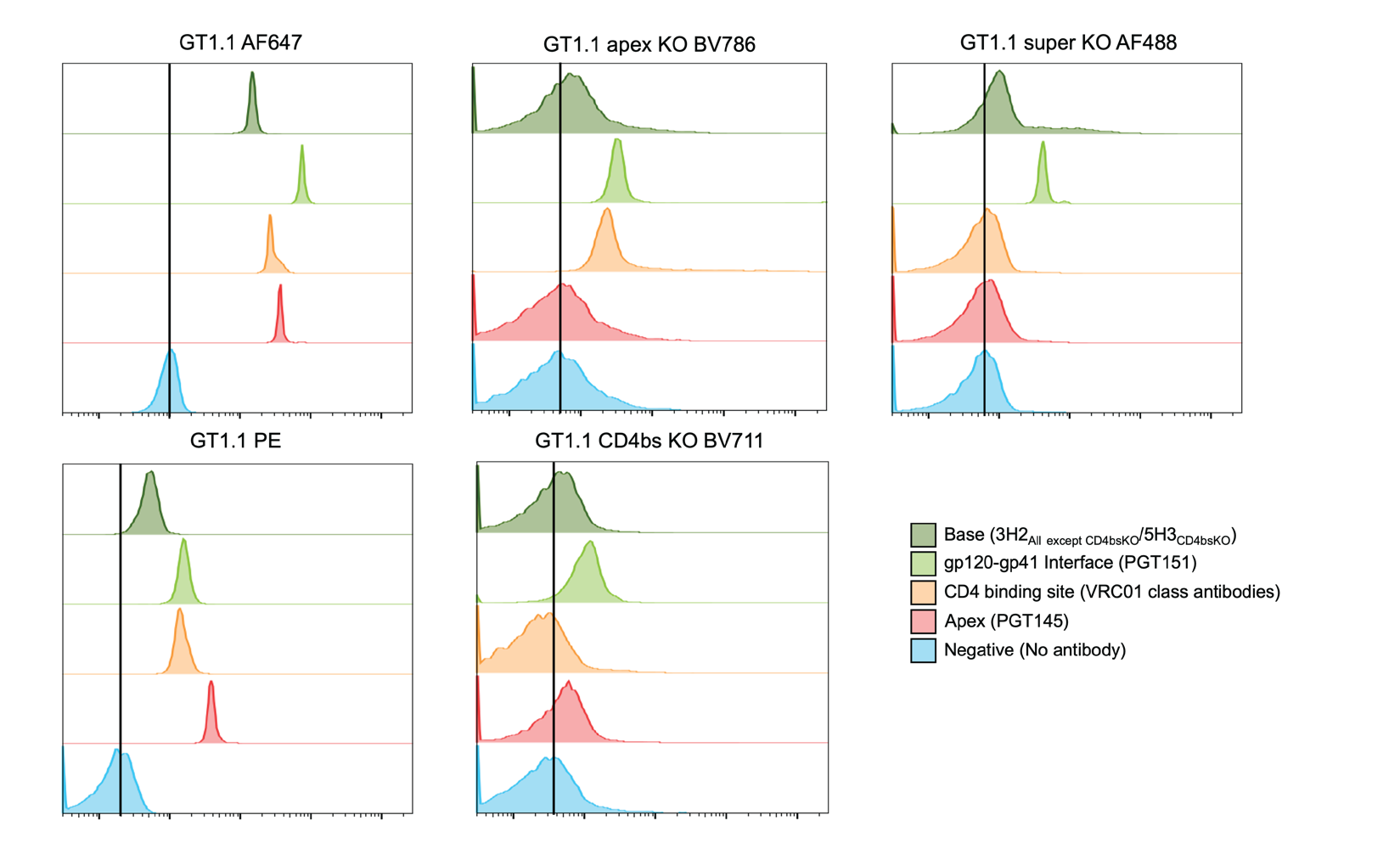
**

**Figure S7. Representative validation experiment of fluorochrome-conjugated B cell probes using bead assay.** Histograms of GT1.1-based probes used in the analysis of antigen and epitope-specific memory B cells. Each of the five panels contain the data for a particular probe (indicated on top of panel), and each histogram represents individual (bn)Abs used to validate the integrity and antigenicity of the probes.


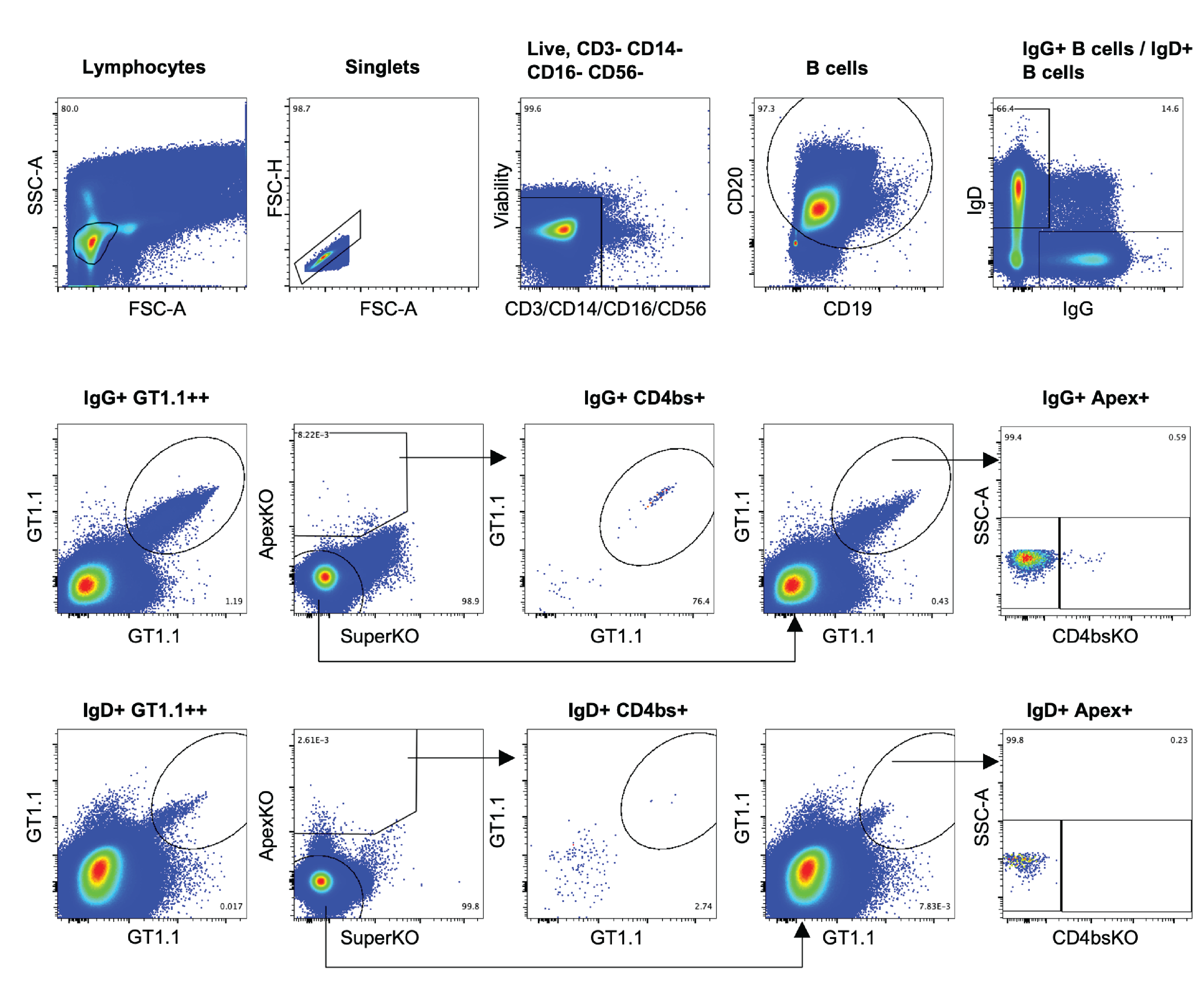


**Figure S8. Representative gating strategy for identification of antigen-specific and epitope-specific B cells.** Flow cytometry plots are provided for one representative individual to demonstrate the gating strategy to distinguish different B cell populations (IgG and IgD) and identify antigen-specific, CD4bs-specific, and Apex-specific B cells within these populations. Samples were processed with the GT1.1 probe set (GT1.1, GT1.1 super KO, GT1.1 apex KO, and GT1.1 CD4bs KO).


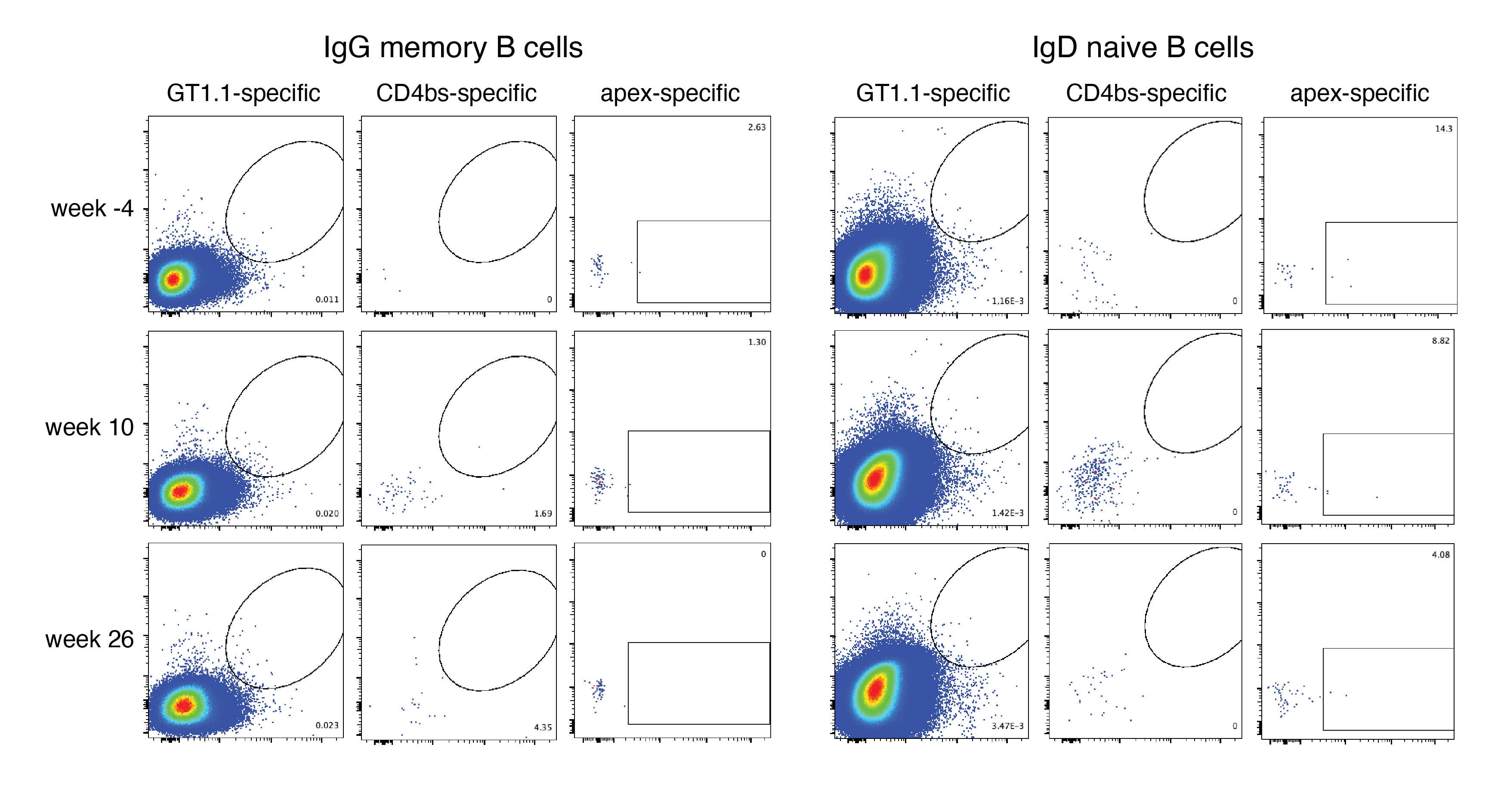

**Figure S9. Representative sequential gating for the placebo group.** The gating was performed as in figure S8 and the numbers on the plots represent frequencies (%) of parent populations.


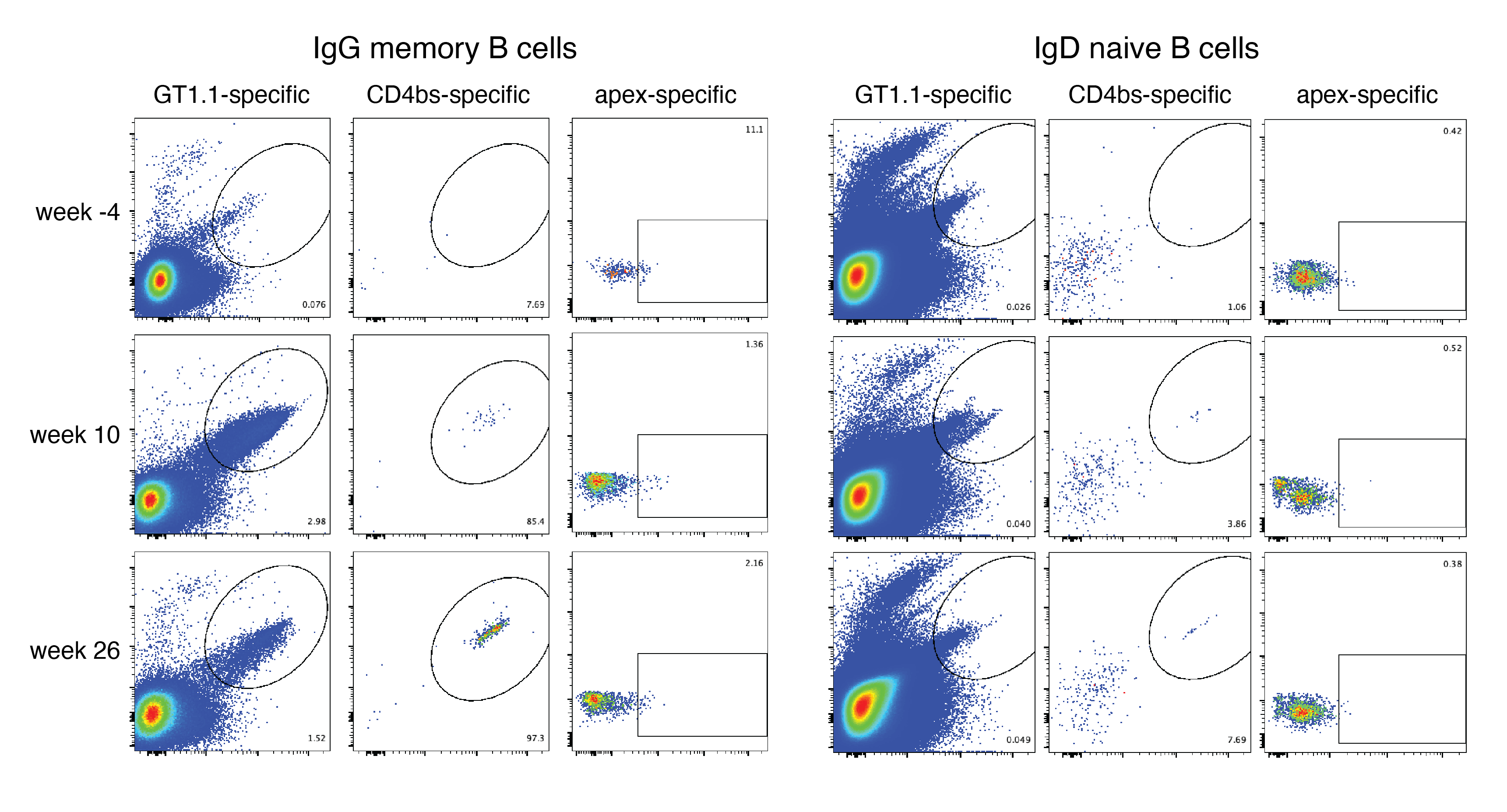


**Figure S10. Representative sequential gating for the low dose vaccine group.** The gating was performed as in figure S8 and the numbers on the plots represent frequencies (%) of parent populations.

**
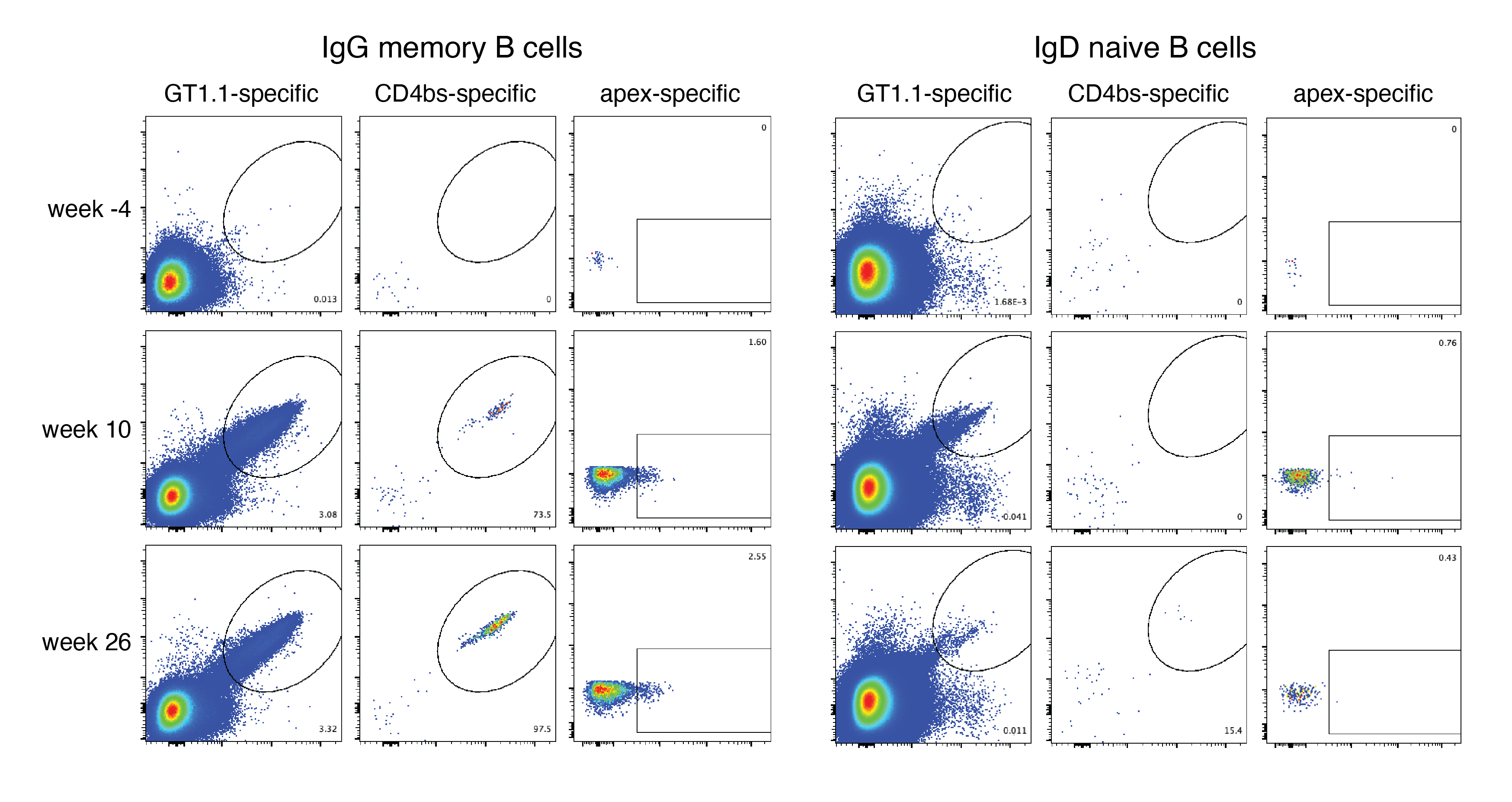
**

**Figure S11. Representative sequential gating for the high dose vaccine group.** The gating was performed as in figure S8 and the numbers on the plots represent frequencies (%) of parent populations.


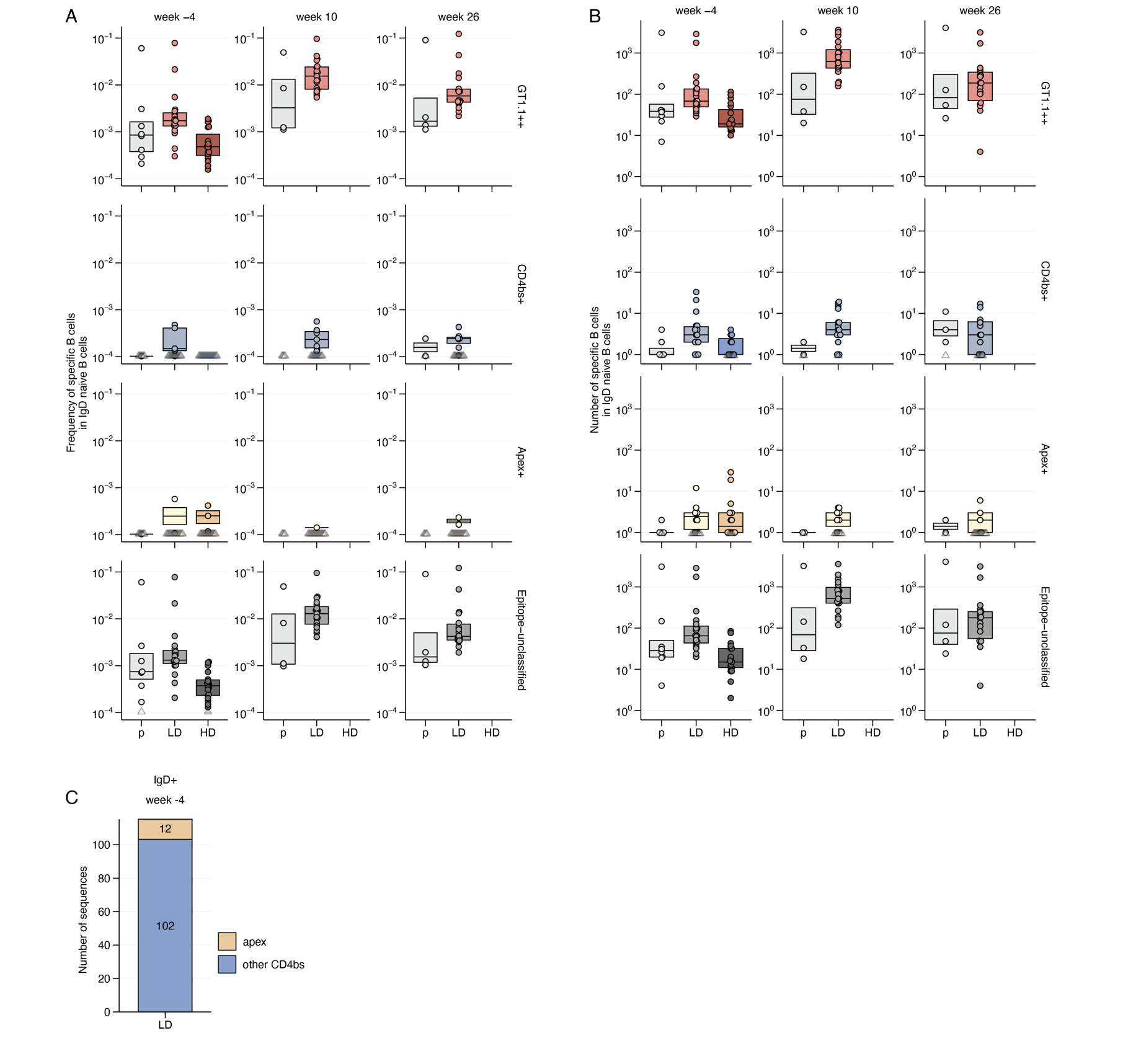


**Figure S12. IgD naive B cell flow cytometry and sequencing data. (A)** Frequency (%) of GT1.1-specific (red), CD4bs-specific (blue), apex-specific (orange) or other specificity (grey) IgD naive B cells in total IgD naive B cells. **(B)** As in (A), but for total numbers of specific B cells detected. **(C)** Total number of sequenced epitope-specific naive IgD B cells. None of the 98 sequenced CD4bs-specific IgD B cells were VRC01-class (IGHV1-2 with a five residue CDRL3). The triangles represent participants with zero measured epitope-specific B cells. The horizontal line represents the median (excluding negative participants) and the edges of the boxplots represent the 25^th^ and 75^th^ percentile.

**
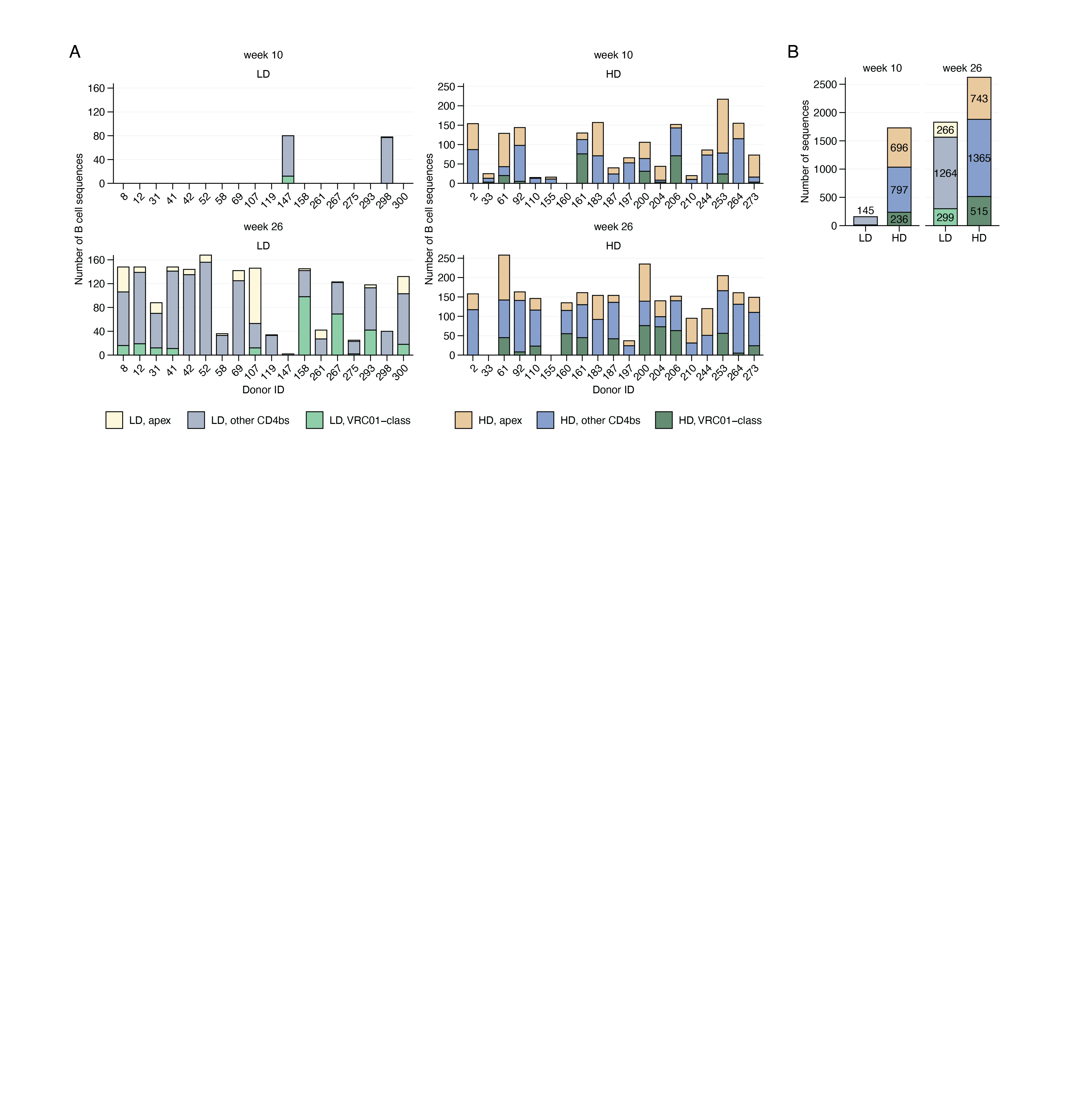
**

**Figure S13. Numbers of B cells included in the final sequencing data set. (A)** Number of B cell sequences that were obtained each vaccine recipient by treatment group (low dose and high dose) and timepoint (week 10 and week 26). Each bar represents the sequence counts from one vaccine recipient, with the color indicating apex-specific, VRC01-class and other CD4bs-specific sequences. **(B)** Total sequences obtained from LD and HD groups (sum across vaccine recipients) at each time point, with color indicating type as in (A).


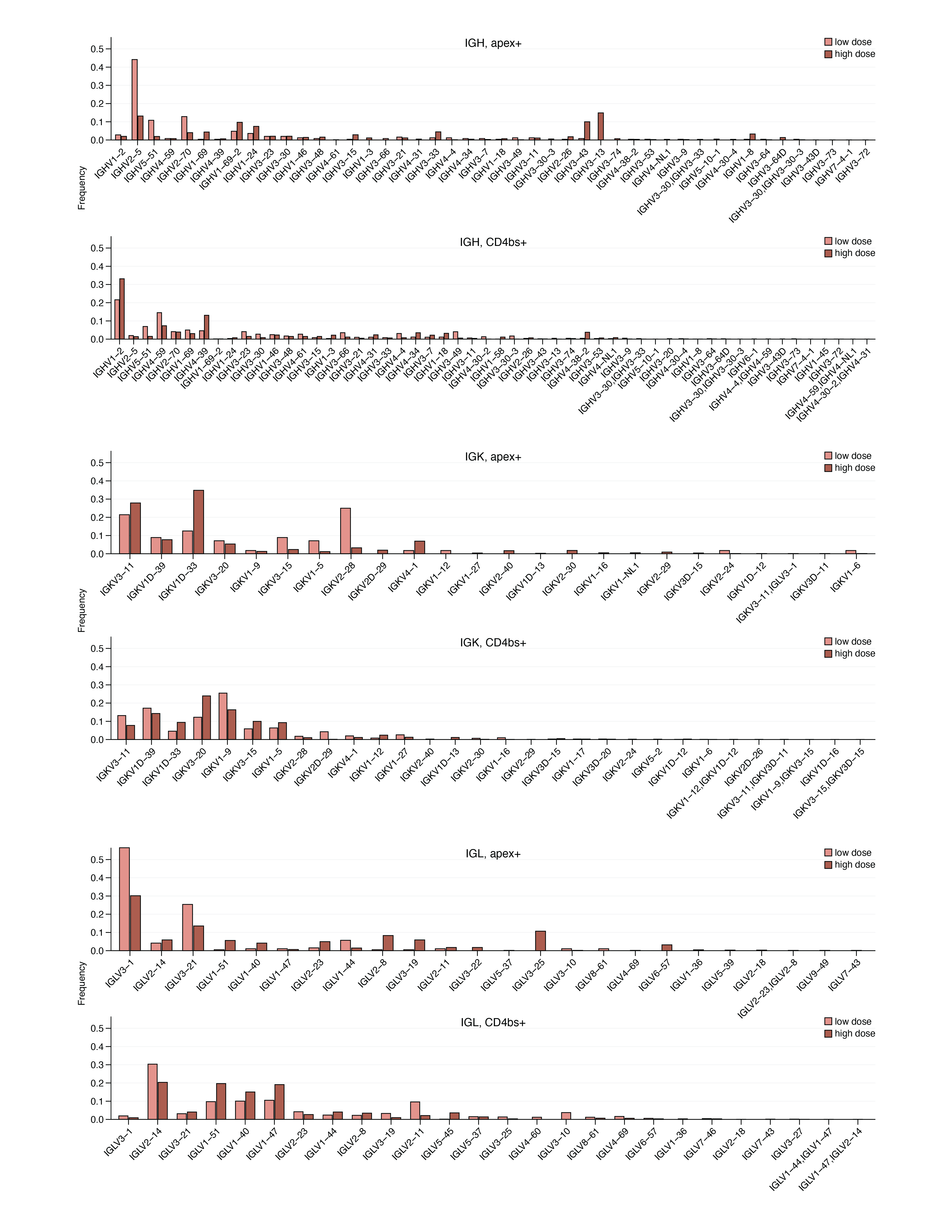


**Figure S14. Frequency of IGHV, IGKV and IGLV gene expression per dose and epitope.** Frequency of heavy and light (kappa and lambda) chain gene usage by dose and epitope specificity (CD4bs-specific/CD4bs+ or Apex-specific/Apex+) is shown.

**
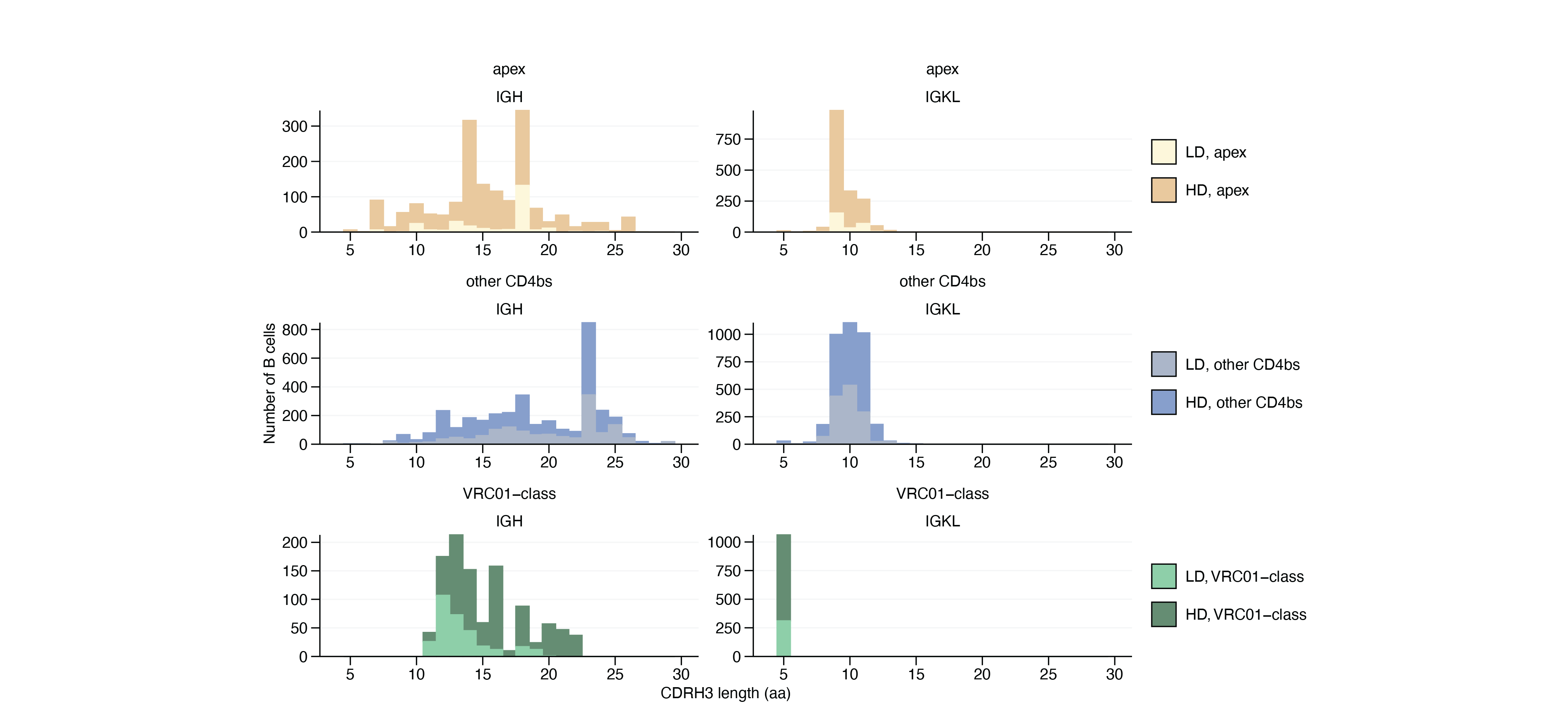
**

**Figure S15. CDR3 lengths of epitope-specific B cell receptors (BCRs).** Histograms showing the number of B cells with particular CDR3 lengths (in amino acids) for the heavy chain (left) and light chains (right).

**
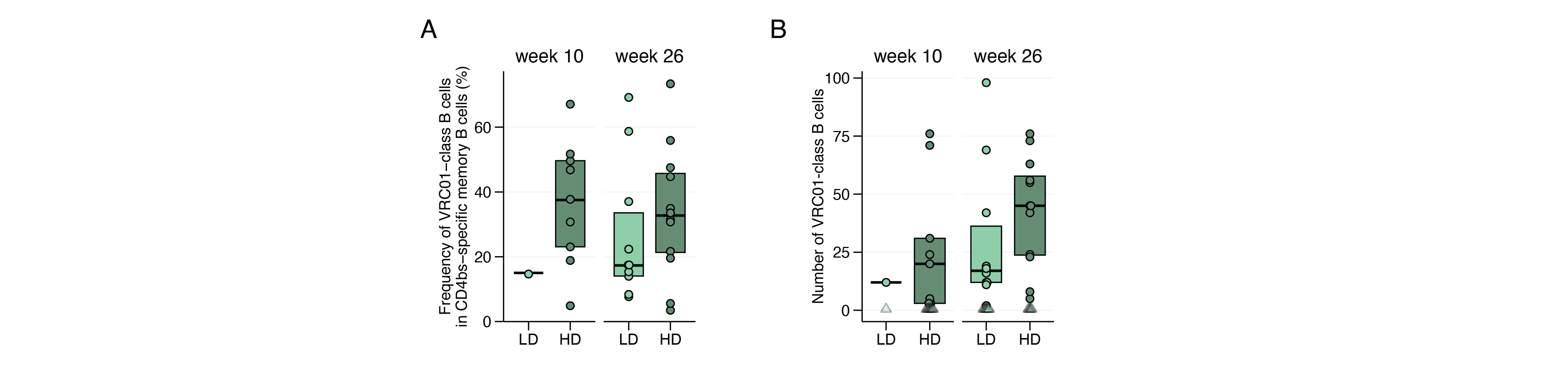
**

**Figure S16. Frequency and number of VRC01-class B cells among CD4bs-specific B cells. (A)** Percentage of VRC01-class B cells among CD4bs-specific IgG memory B cells in responders. Symbols represent a single participant at one timepoint. Boxplots were constructed using positive VRC01-class responders. **(B)** Number of VRC01-class B cells among all IgG memory B cells. Symbols represent a single participant at one timepoint. Triangles represent donors with no VRC01-class B cell response. Boxplots were constructed using positive VRC01-class responders. In **(A)** and **(B)**, thick lines are medians and box plots show 25% and 75% quantiles for each dose group at each timepoint.


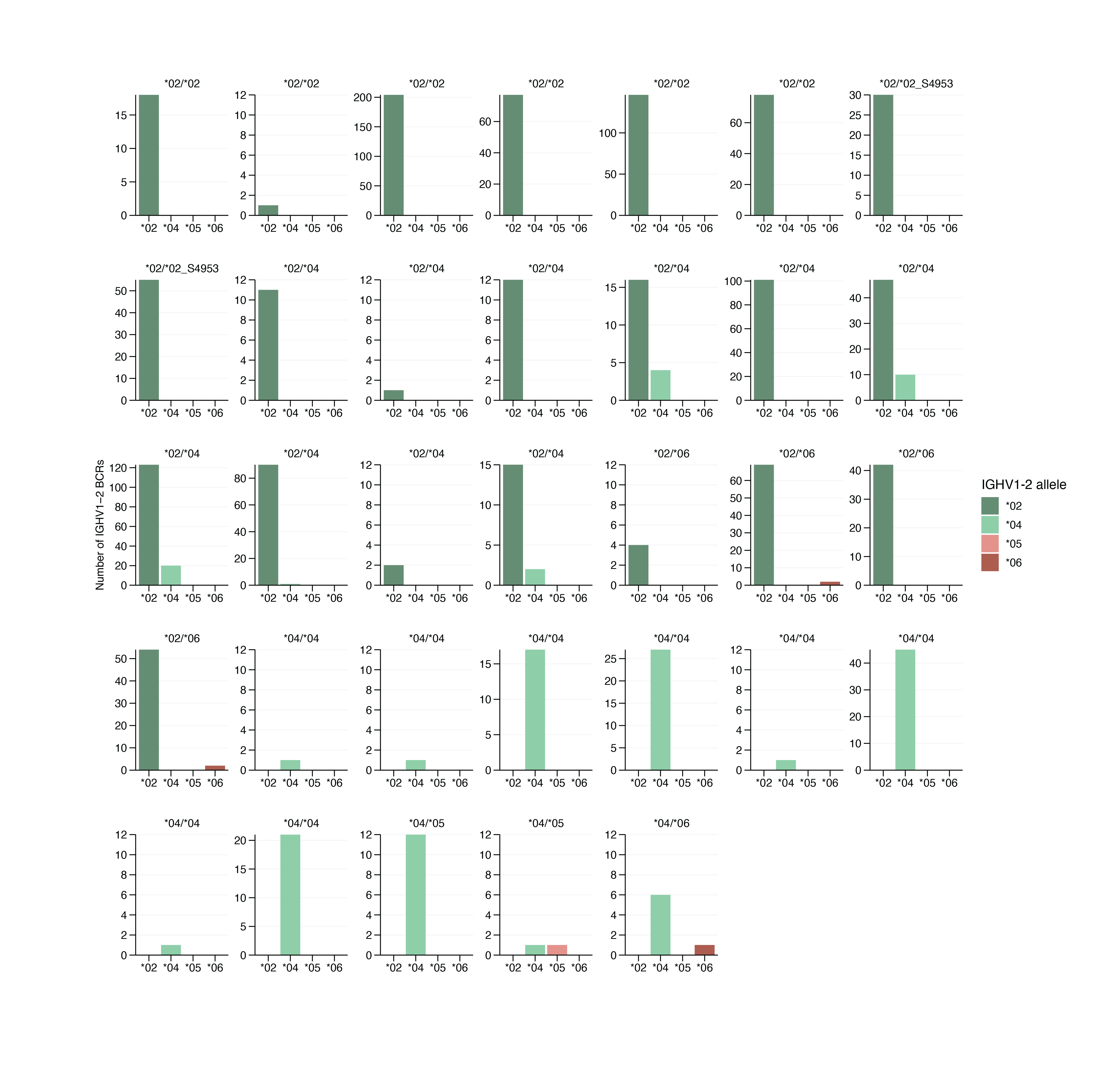


**Figure S17. Frequency of IGHV1-2 B for vaccine recipients with IGHV1-2 B cells.** Each graph shows the IGHV1-2 genotype (*e.g.*, *02/*02) and the number of IGHV1-2 B cells with specific alleles isolated from a particular trial participant. Each graph shows a different participant. The colors indicate specific alleles; *02 and *04 are permissive (green), while *05 and *06 are non-permissive (red) for VRC01-class recognition of the CD4bs.


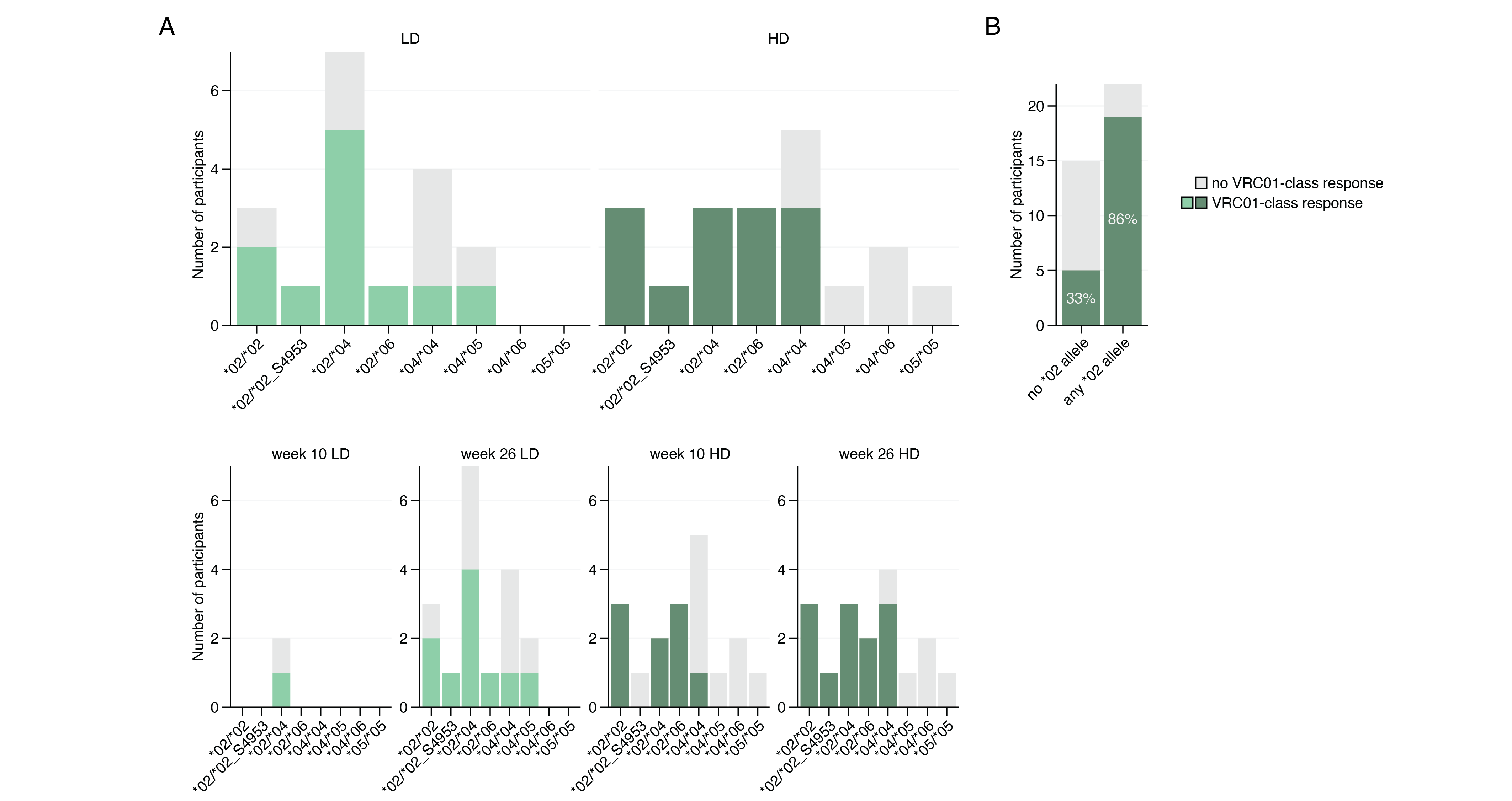


**Figure S18. VRC01-class response rate per IGHV1-2 allele. (A)** Number of participants per dose group within each genotype that had at least one VRC01-class B cell detected (green) or no VRC01-class B cells detected (grey). **(B)** Number of participants with and without the presence of a *02 allele that had at least one VRC01-class B cells detected.


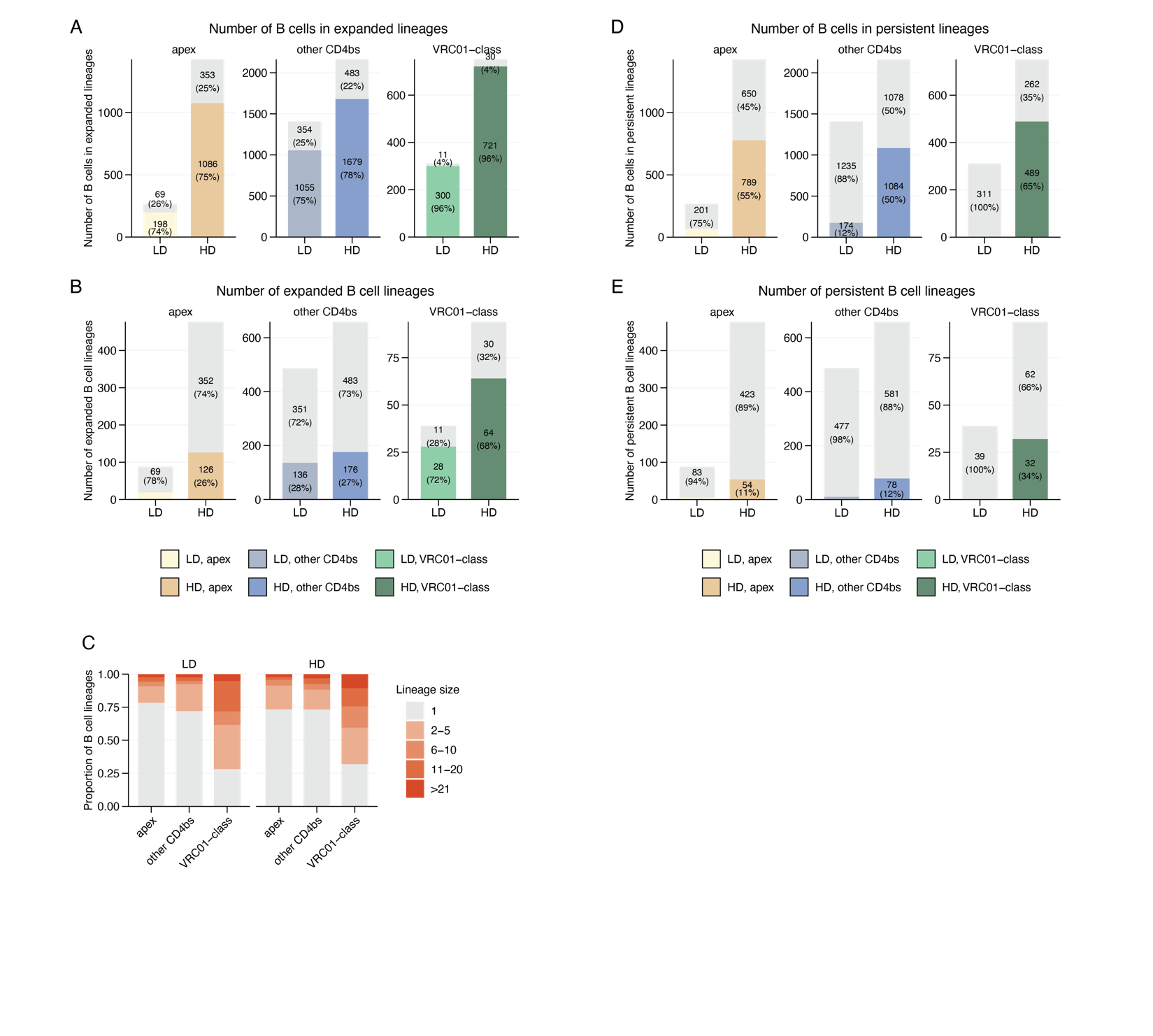


**Figure S19. Clonal expansion and persistence of all epitope specificities. (A)** Number of B cells in expanded lineages by antigen specificity and dosage group. Expanded lineages are defined as consisting of ≥2 members. **(B)** Number of expanded lineages by antigen specificity and dosage group. For **(A)** and **(B)**, grey areas of the bars denote non-expanded lineages. **(C)** Number of B cells in persistent lineages by antigen specificity and dosage group. Persistent lineages are defined as ones from which members are detected across multiple timepoints (weeks 10 and 26). **(D)** Number of persistent B cell lineages by antigen specificity and dosage group. For **(C)** and **(D)**, grey areas of the bars denote non-persistent lineages. For all graphs, the numbers in the graphs represent the number of B cells (A and C) or the number of B cell lineages (B and D), and the frequency is shown as percentages.


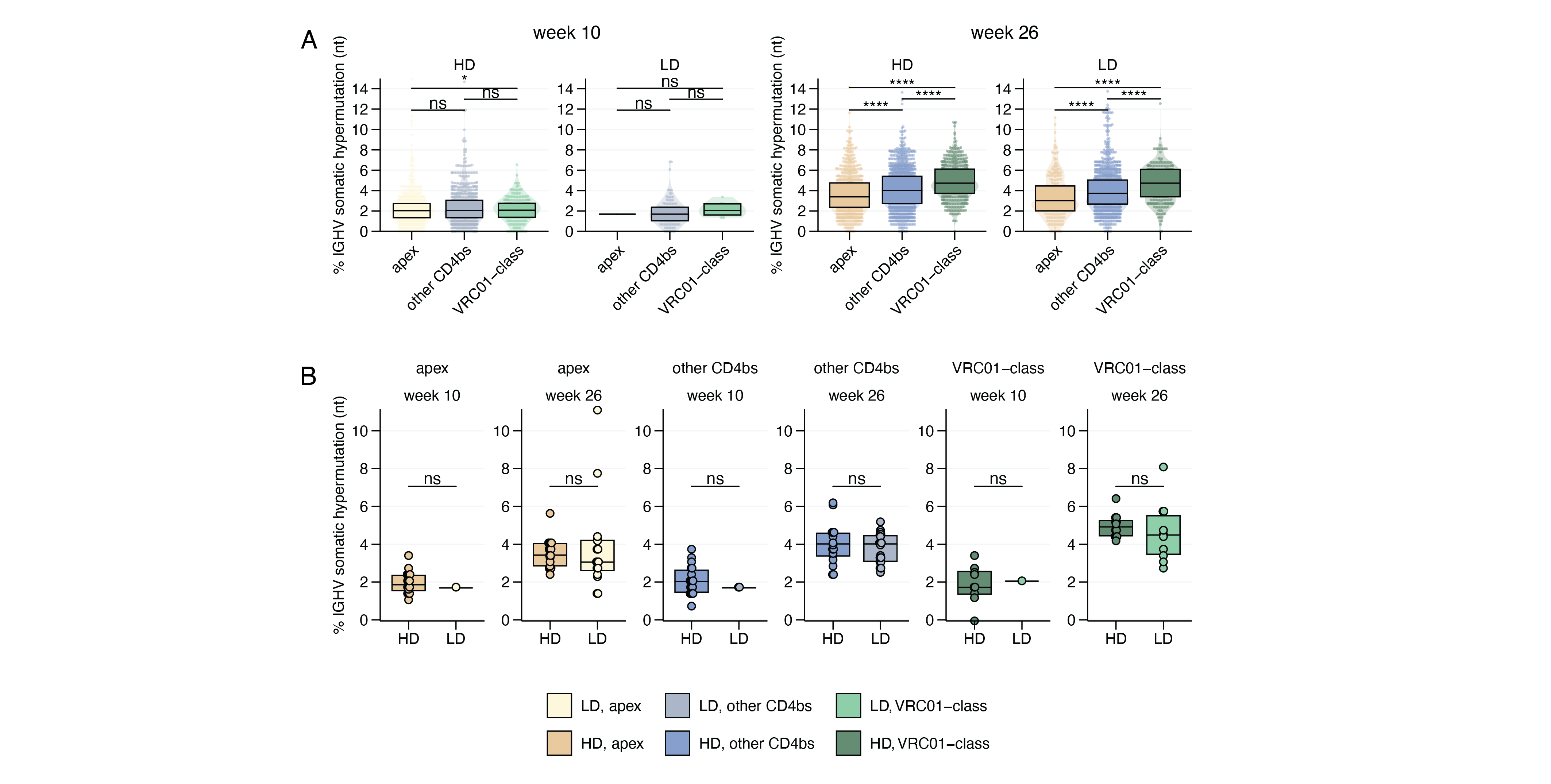


**Figure S20. Additional somatic hypermutation analysis.** Each dot represents one trial participant. All statistical tests: Wilcoxon test: ns, not significant, *, p < 0.05, ***, p < 0.001, ****, p < 0.0001. Thick lines are medians and box plots show 25 and 75% quantiles for each dose group at each time point. Percent somatic hypermutation (SHM) expressed as a frequency of nucleotides mutated compared to germline sequences. **(A)** BCR IGHV percentage nucleotide hypermutation for week 10 (left) and week 26 (right) for apex-specific, CD4bs-specific and VRC01-class B cells, respectively. **(B)** BCR IGHV percentage nucleotide hypermutation in low dose and high dose groups at weeks 10 and week 26 for apex-specific, CD4bs-specific and VRC01-class B cells, respectively.


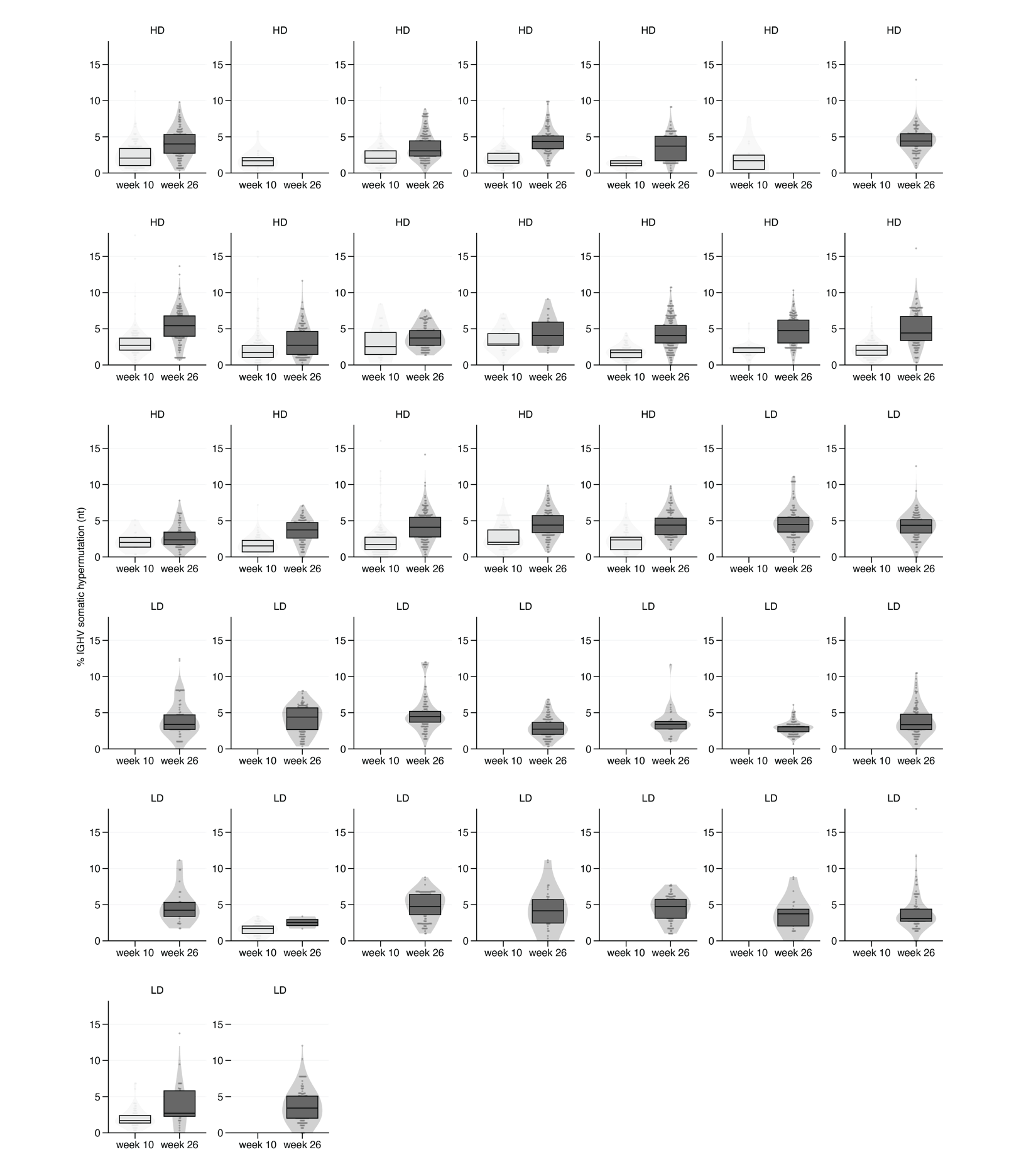


**Figure S21. Somatic hypermutation per week and per trial participant.** Percentage BCR IGHV nucleotide hypermutation from selected low dose and high dose participants at weeks 10 and 26. Each plot represents a single participant. SHM expressed as a frequency of nucleotides mutated compared to germline sequences. Thick lines are medians and box plots show 25 and 75% quantiles for each dose group at each time point.


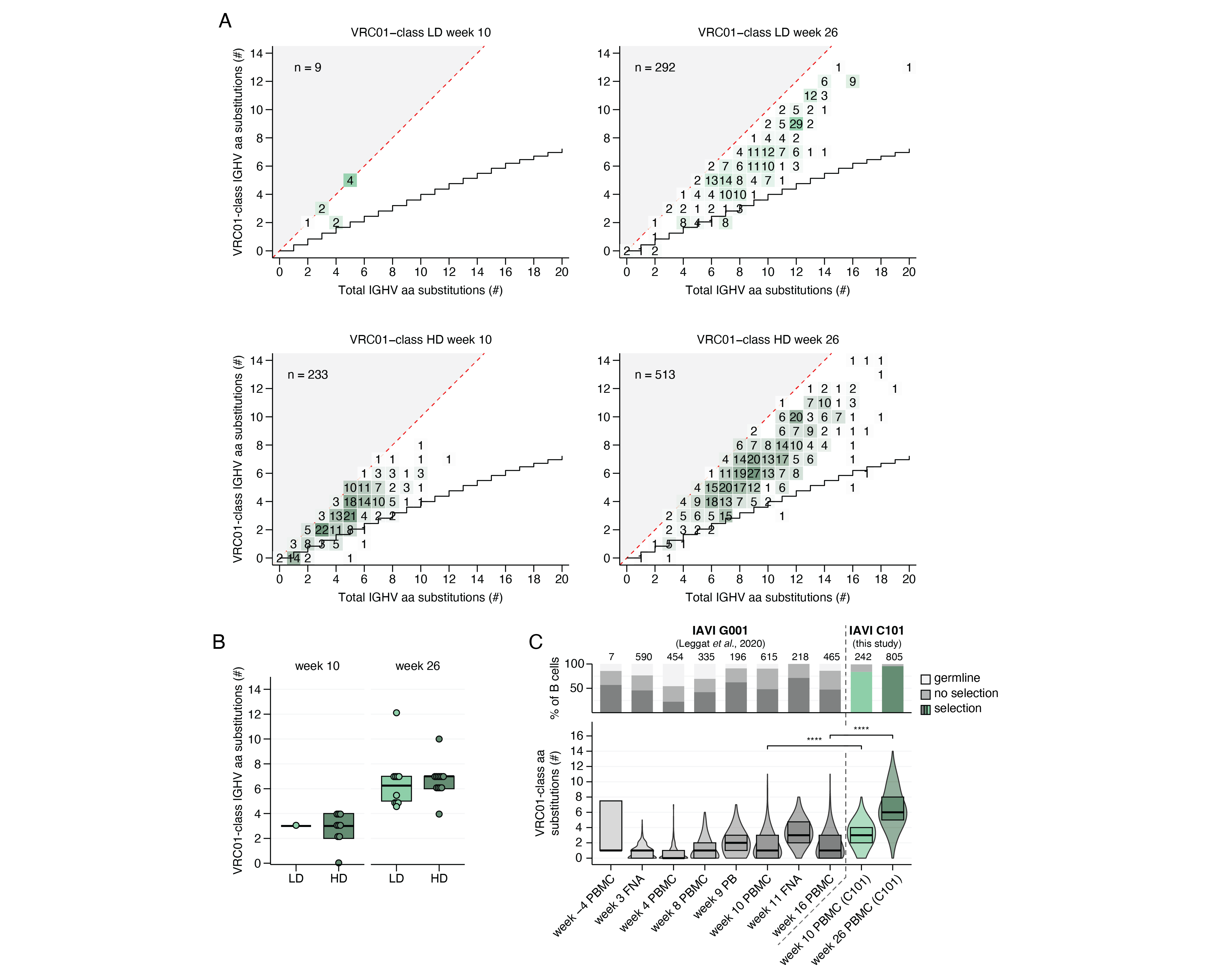
**Figure S22. Additional VRC01-class mutation analyses. (A)** VRC01-class IGHV amino acid substitutions and total IGHV amino acid substitutions for low dose group (top) and high dose group (bottom) at weeks 10 and 26. **(B)** VRC01-class IGHV amino acid substitutions for the low dose and high dose groups at weeks 10 (left) and 26 (right). Thick lines indicate median values, box plots show 25% and 75% quantiles. Each dot represents a vaccine recipient. **(C)** Top panel: frequency of BCRs (%) that are germline (white), that do not demonstrate selection for VRC01-class mutations (light grey) or that do demonstrate selection for VRC01-class mutations (dark grey and green). The numbers indicate the total number of sequences for each time point and material. Bottom panel: number of VRC01-class amino acid substitutions in samples from IAVI G001 (grey) and IAVI C101 (green). Wilcoxon test: ****, p < 0.0001.


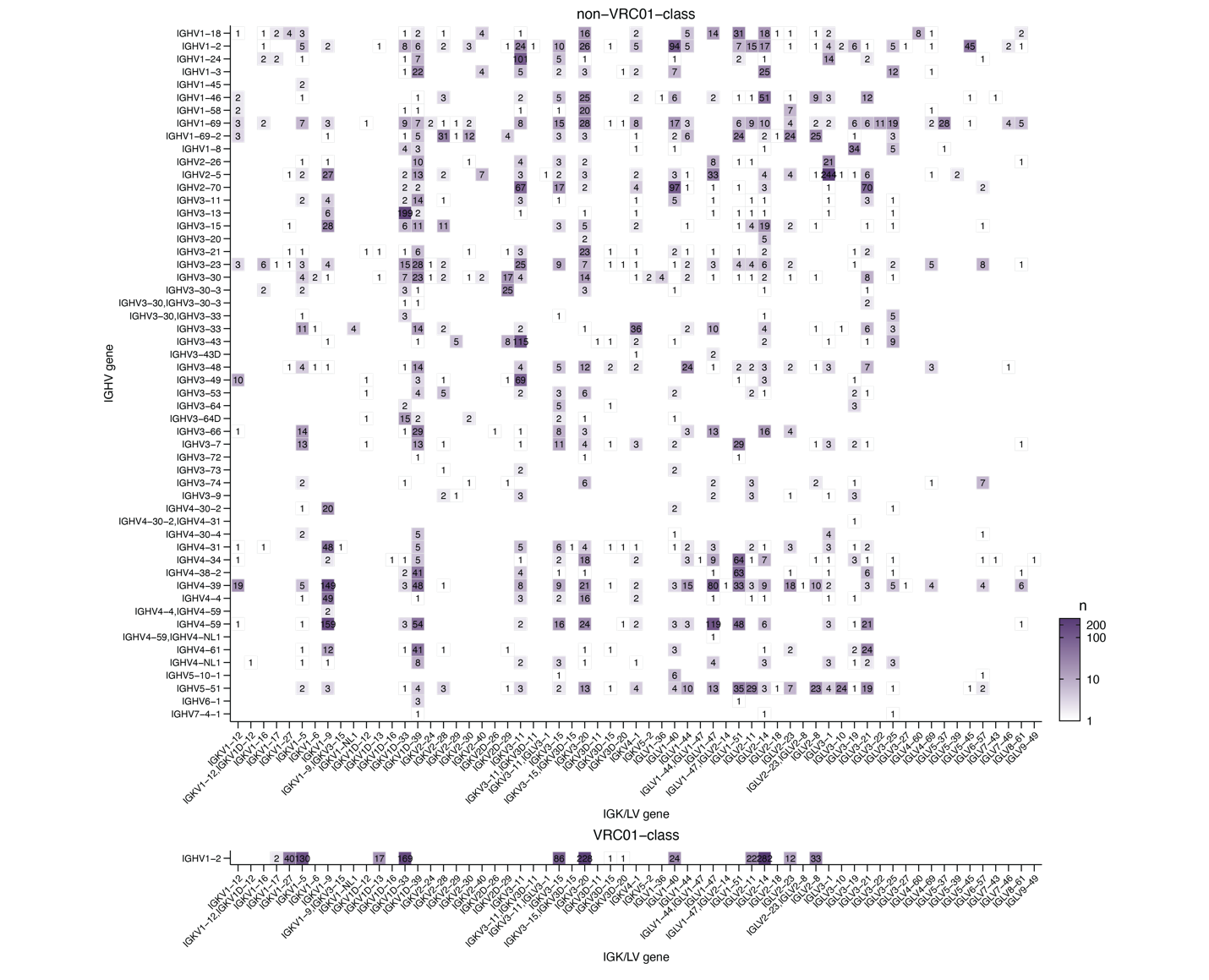


**Figure S23. Frequency of heavy chain and light chain variable gene pairings.** Top panel: non-
VRC01 variable gene pairings. Each row corresponds to a IGHV gene and each column to a IGLV or IGKV gene. Bottom panel: VRC01-class variable gene pairings. VRC01-class BCRs show a predominance of known VRC01-class bnAb light chain genes but are also paired with non-prototypic VRC01-class light chains such as IGKV1-17 and IGKV1-27.


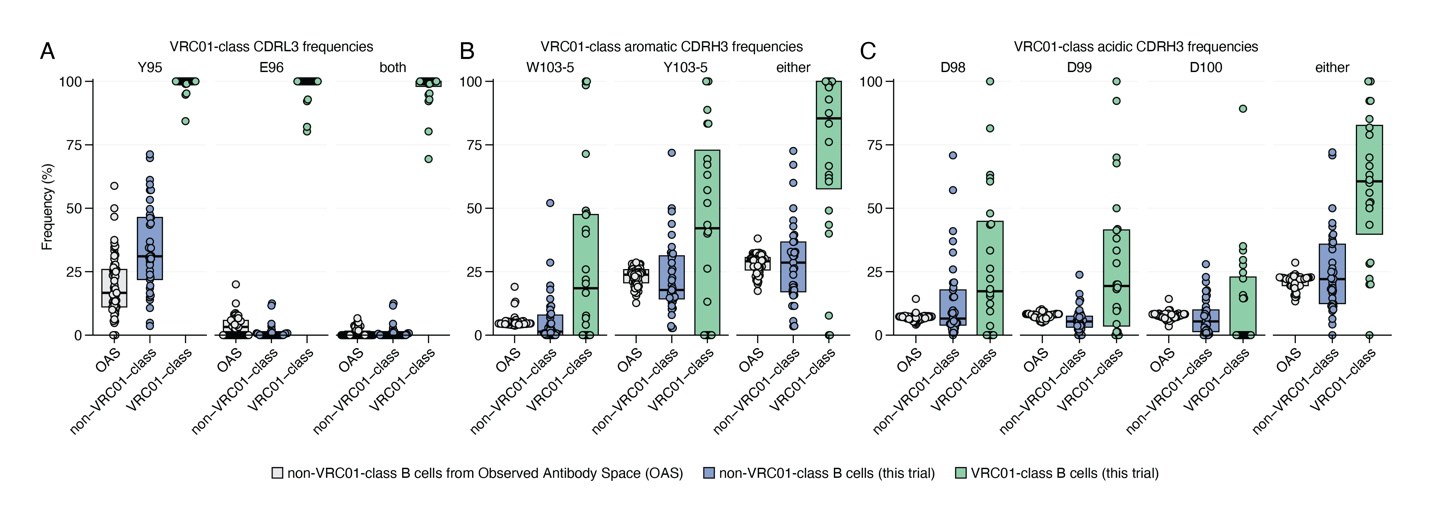


**Figure S24. Frequencies of signature VRC01-class bnAb residues. (A)** Frequency of VRC01-class amino acid residues Y95_CDRL3_ and E96_CDRL3_ in the CDRL3 region of the Observed Antibody Space, non-VRC01-class and VRC01-class B cells. **(B)** Frequency of VRC01-class aromatic amino acid residues W103-5_CDRH3_ and Y103-5_CDRH3_ in the CDRH3 of the Observed Antibody Space, non-VRC01-class and VRC01-class B cells. **(C)** Frequency of VRC01-class acidic amino acidic residues D98_CDRH3_, D99_CDRH3_, D100_CDRH3_ in the CDRH3 gene of the Observed Antibody Space, non-VRC01-class and VRC01-class B cells. See Methods for details on the Observed Antibody Space data set.


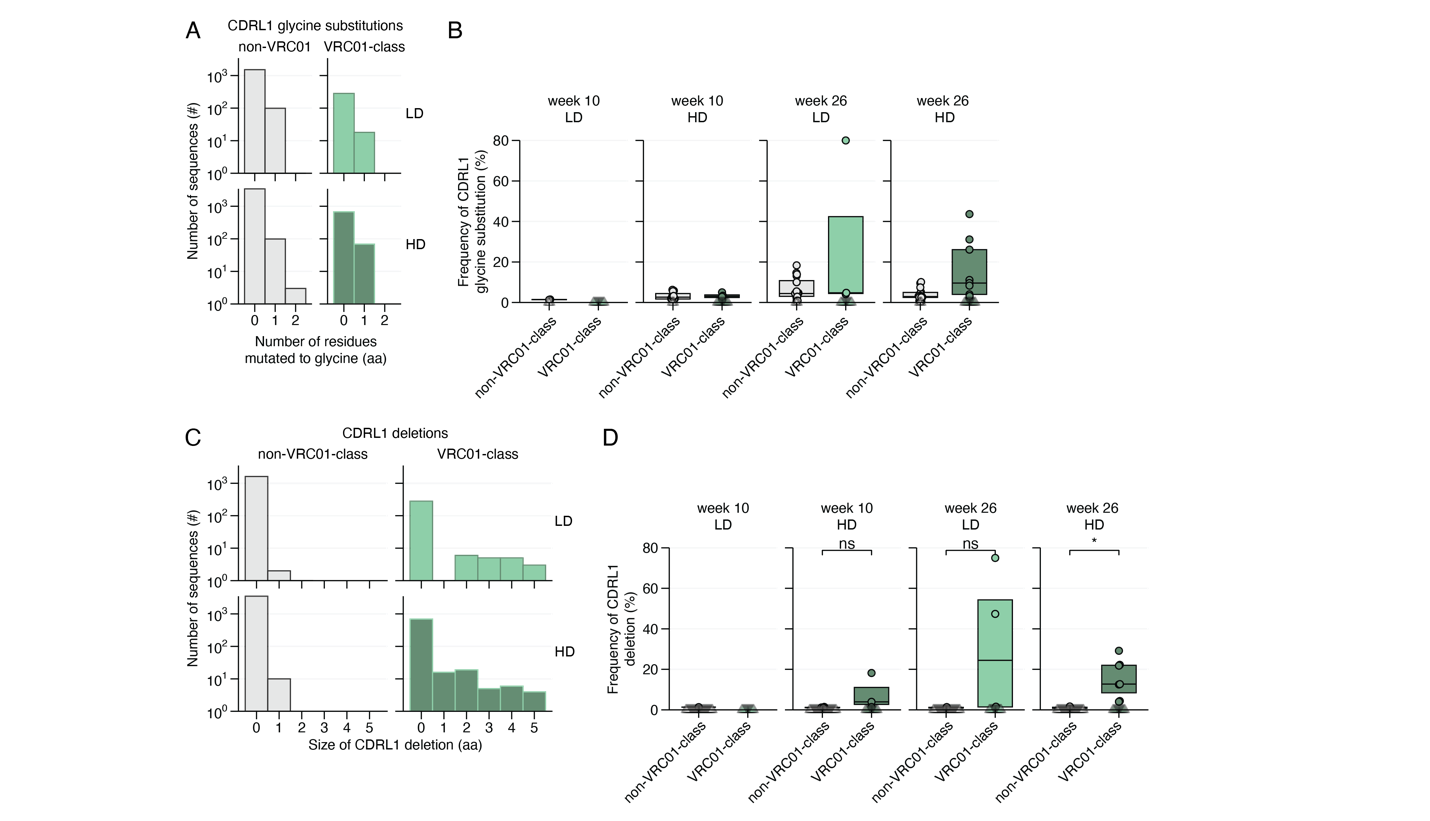


**Figure S25. Additional analyses on CDRL1 glycine substitutions and deletions. (A)** Number of VRC01-class (green) and non-VRC01-lass (grey) sequences containing CDRL1 glycine substitutions in the low dose (top) and high dose groups (bottom). **(B)** Frequency of CDRL1 glycine substitutions at weeks 10 and 26 in non-VRC01-class (grey) and VRC01-class (green) sequences **(C)** Number of non-VRC01-class (grey) and VRC01-class (green) B cells with CDRL1 deletions in the low dose (top) and high dose (bottom) groups. **(D)** Frequency of CDRL1 deletions in non-VRC01-class (grey) and VRC01-class (green) sequences in low dose and high dose groups at weeks 10 and 26. Wilcoxon rank-sum test: ns, not significant, *, p < 0.05.


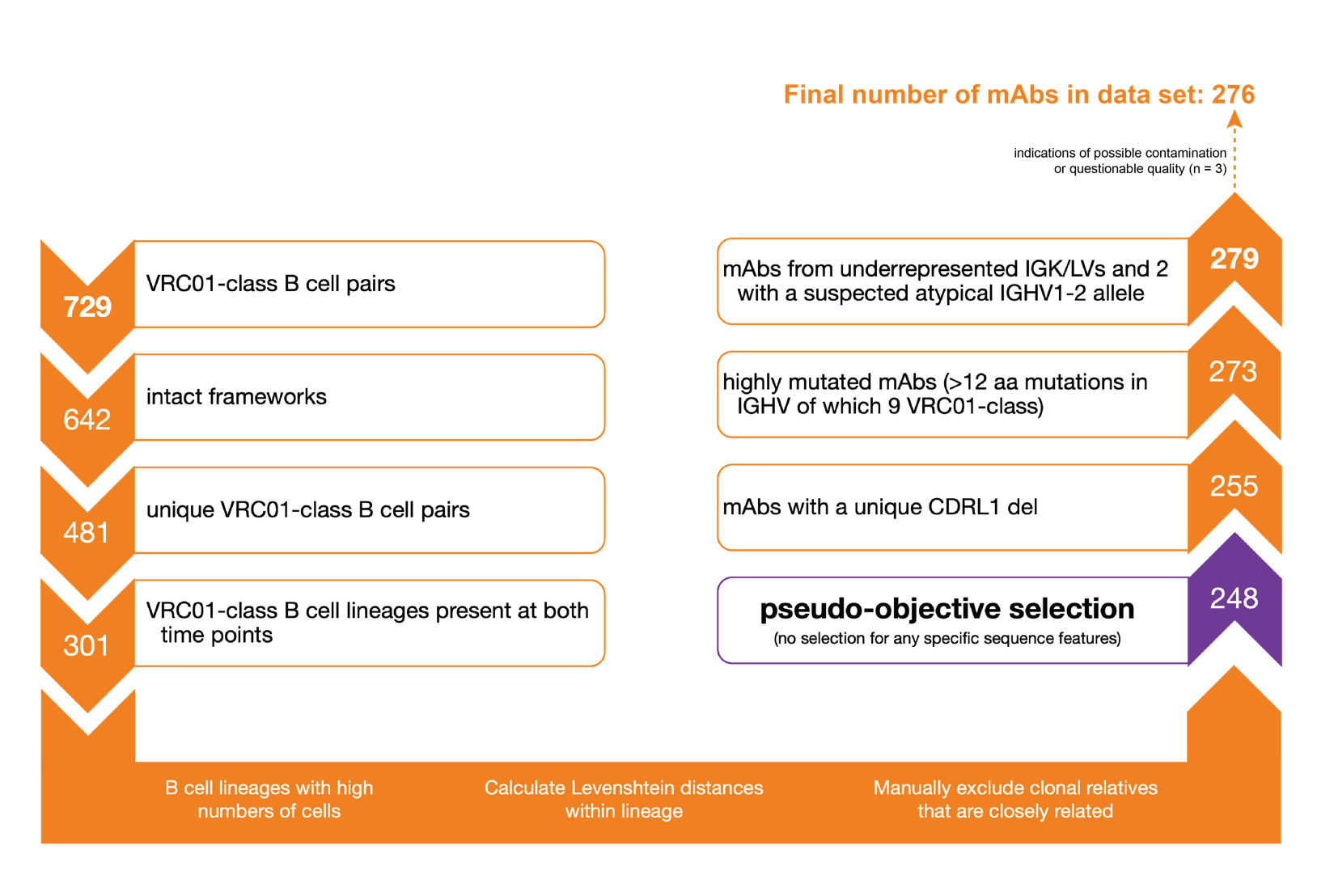


**Figure S26. Schematic depiction of the selection of VRC01-class mAbs for expression.** See Methods for details.


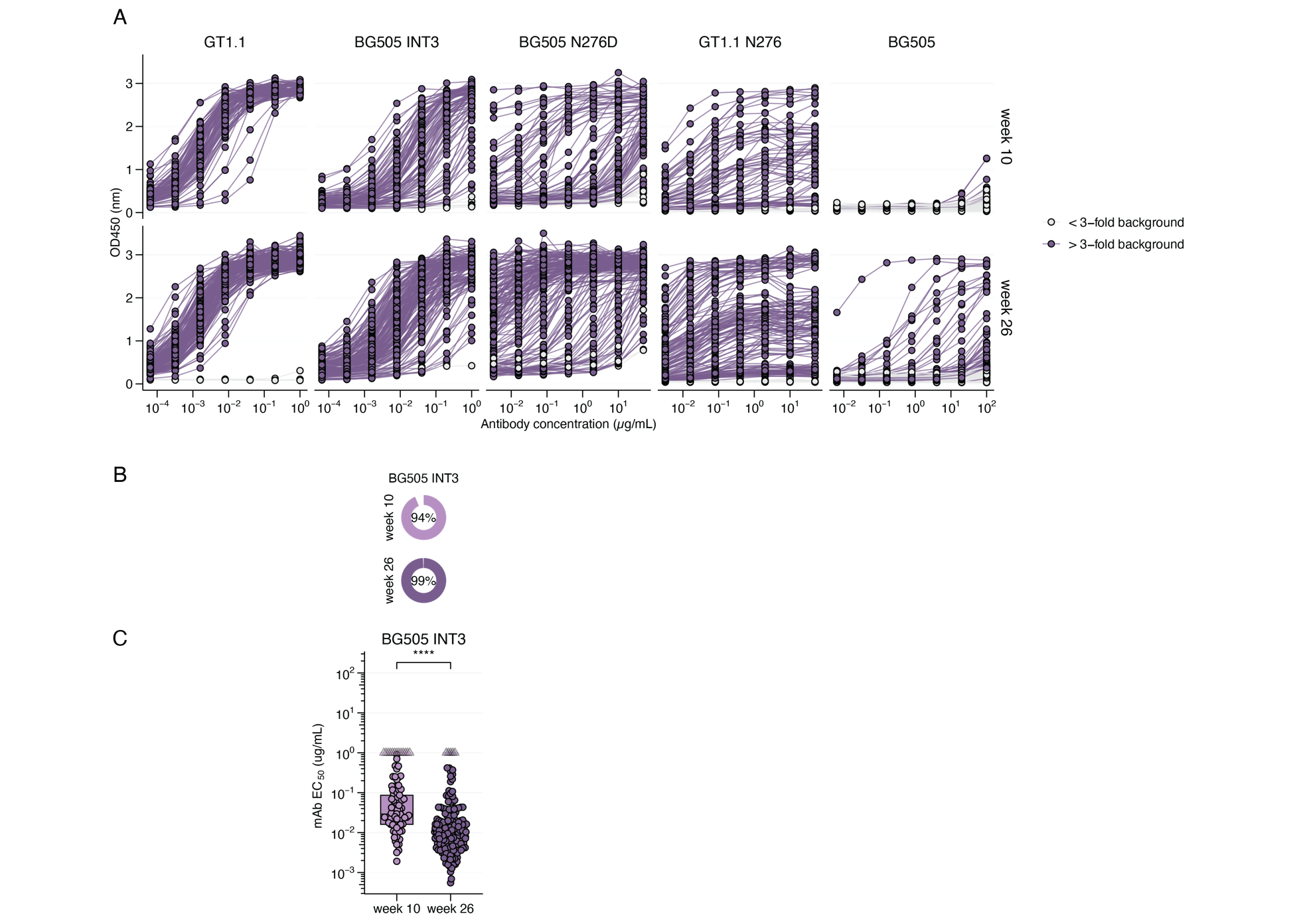


**Figure S27. VRC01-class mAb analysis by enzyme-linked immunosorbent assay (ELISA) for homologous BG505 SOSIP Env trimers.** All statistical tests: Wilcoxon test: ****, p < 0.0001 **(A)** ELISA binding data for selected VRC01-class mAbs from week 10 (top row) or week 26 (bottom row) at increasing concentrations to the indicated trimers. Absorbance was measured at 450 nm (see Methods for details). Each dot represents a single dilution of a tested mAb**;** each mAb is connected by a line. **(B)** Pie charts representing the proportion of mAbs that showed binding to the indicated Env trimer at least ≥3-fold above background at the highest concentration. **(C)** Half-maximal binding titers (EC_50_) of selected VRC01-class mAbs against the trimer (BG505 INT3) as indicated on top of the graph. Triangles indicate mAbs that did not bind at the indicated concentration and were thus considered to have a value larger than the highest tested value.

**
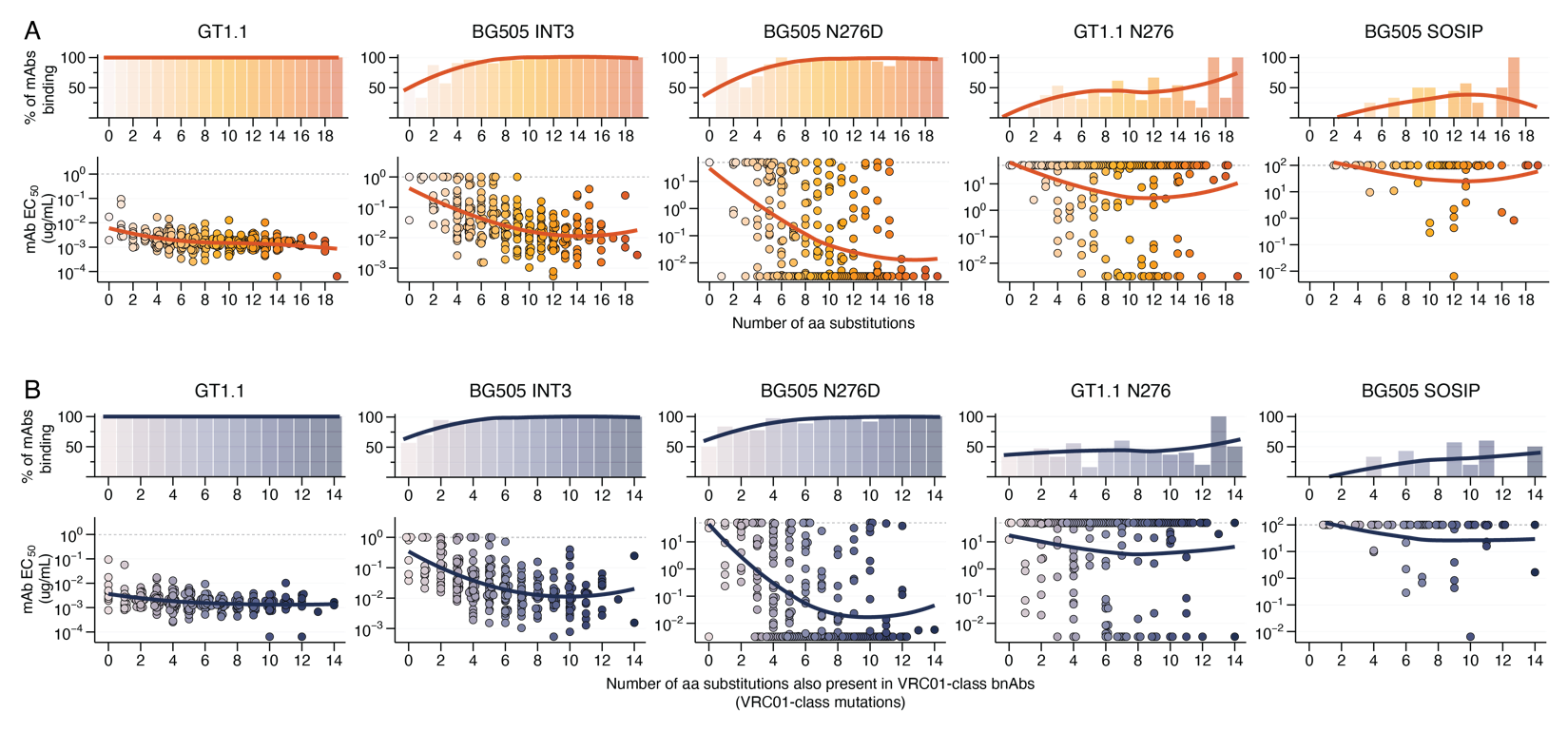
**

**Figure S28. Apparent binding affinity correlates with (VRC01-class) somatic hypermutation.** Each dot represents a single tested mAb. **(A)** Top row: percentages of mAbs with certain numbers of amino acid substitutions (x-axis) binding to the indicated trimer. Bottom row: correlation between the percentage of mAbs that bind the indicated trimer and the half-maximal binding titers (EC_50_) of selected mAbs to the indicated trimer in relation to the number of amino acid substitutions. **(B)** As in (A), but for VRC01-class amino acid substitutions as in Figure 3.


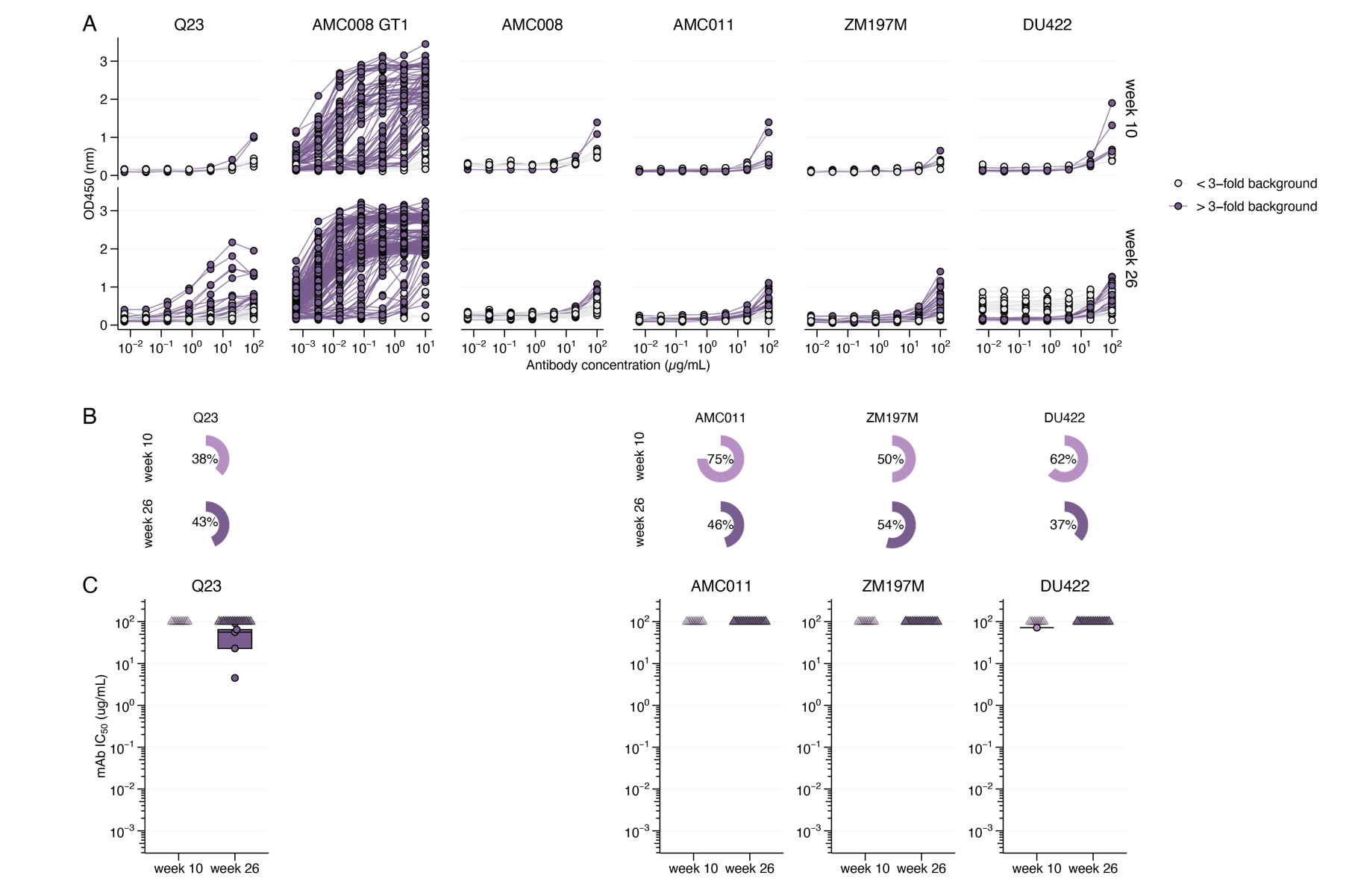


**Figure S29.** **VRC01-class mAb analysis by enzyme-linked immunosorbent assay (ELISA) for heterologous SOSIP trimers.** Each dot represents a single tested mAb. **(A)** ELISA binding data for selected VRC01-class mAbs from week 10 (top row) or week 26 (bottom row) at increasing concentrations to the indicated trimers. Absorbance was measured at 450 nm (see Methods for details). **(B)** Pie charts representing the proportion of mAbs that showed binding to the indicated Env trimer at least ≥3-fold above background at the highest concentration. **(C)** Half-maximal binding titers (EC_50_) of selected VRC01-class mAbs against the trimers as indicated on top of the graph. Triangles indicate mAbs that did not bind at the indicated concentration and were thus considered to have a value larger than the highest tested value.


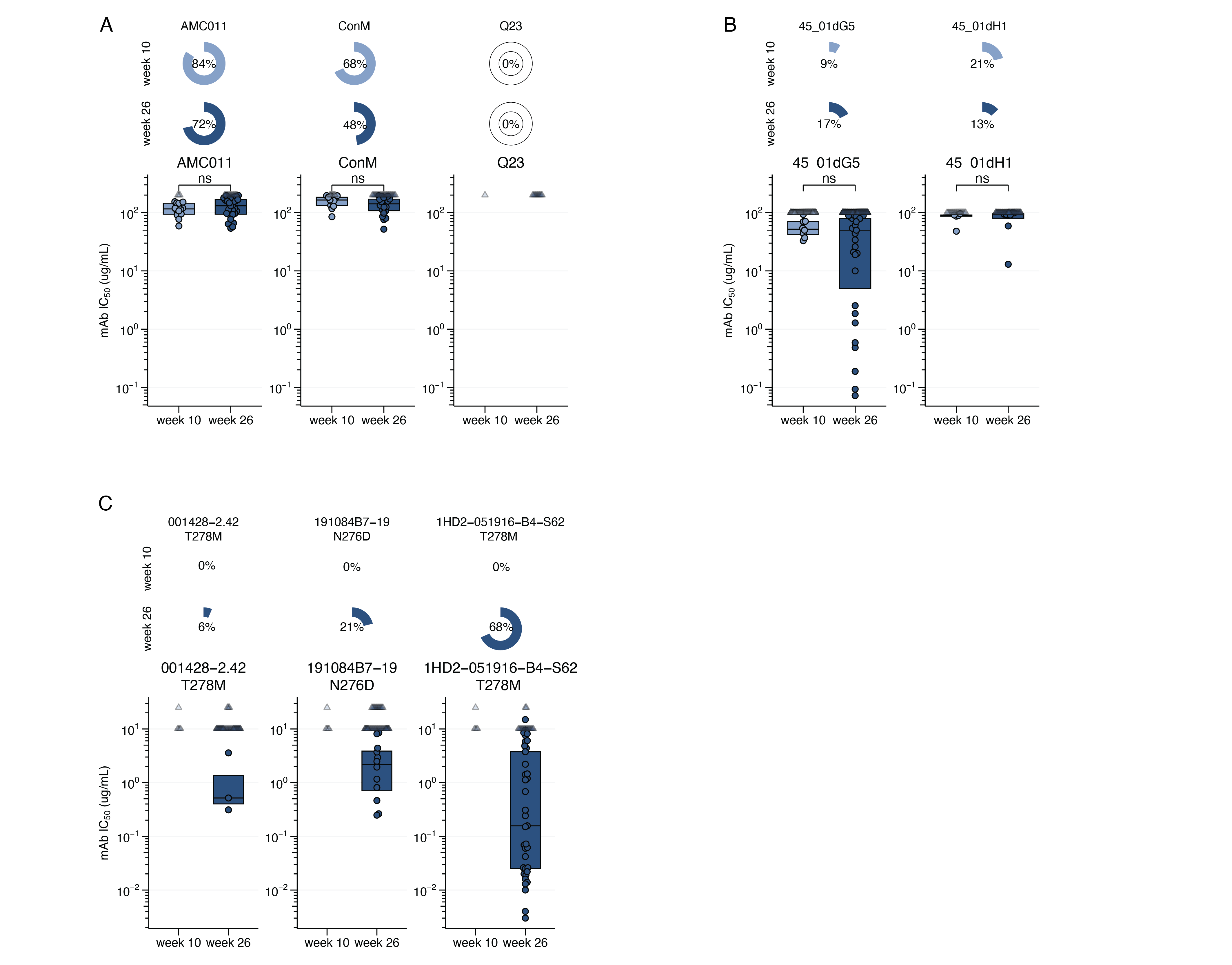


**Figure S30.** **VRC01-class mAb analysis by heterologous pseudovirus neutralization assay.** Each dot represents a single tested mAb. All statistical tests: Wilcoxon test: ns, not significant. **(A-C)** Top rows: pie charts representing the proportion of mAbs that showed neutralization to the indicated pseodoviruses (PVs). Neutralization was considered to occur when an IC_50_ could be derived. Bottom rows: half-maximal inhibitory titers (IC_50_) of selected VRC01-class mAbs in a pseudovirus neutralization assay against the indicated PVs. Triangles indicate mAbs that did not neutralize at the indicated concentration and were thus considered to have a value larger than the highest tested value.


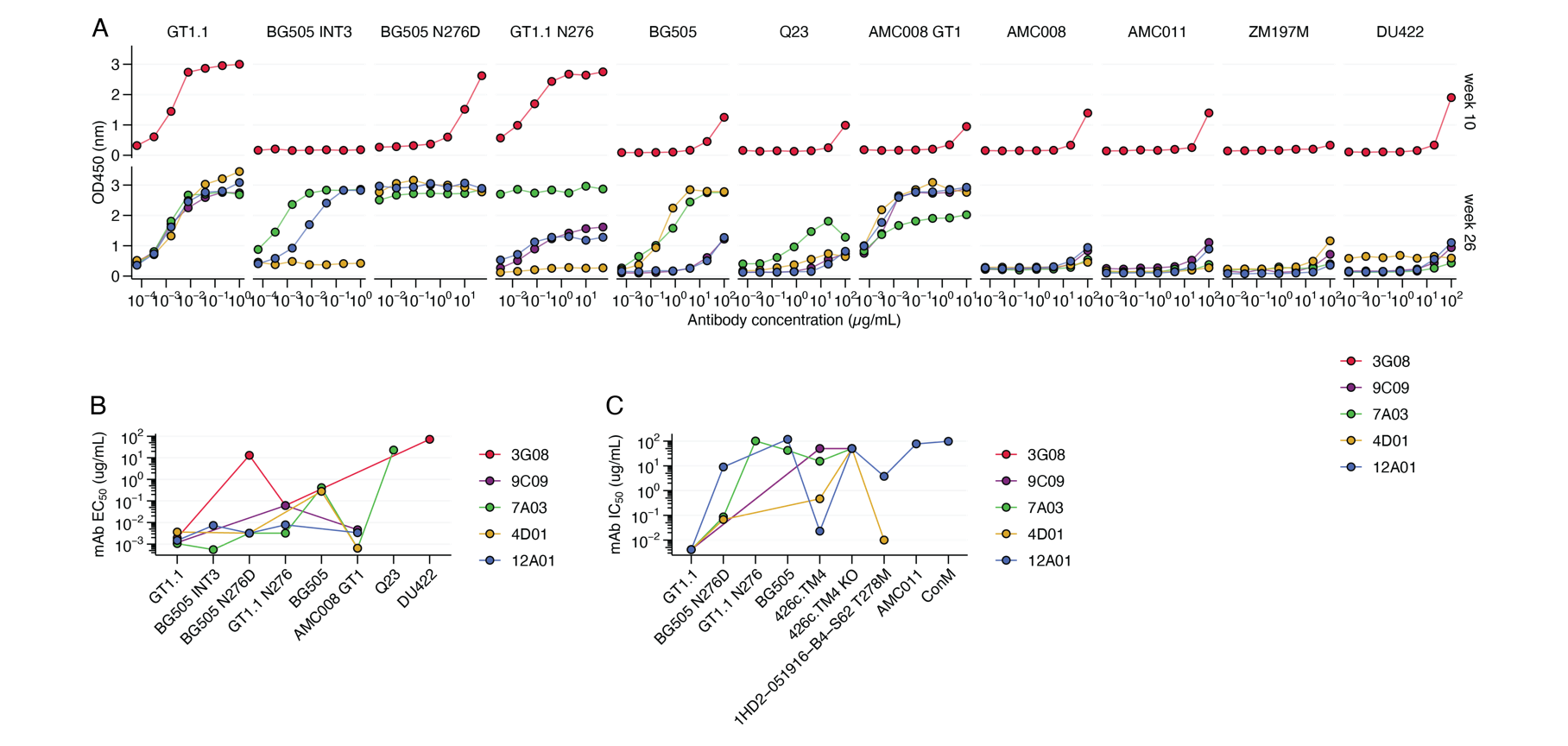


**Figure S31. Additional binding and neutralization data for VRC01-class mAbs selected for structural studies. (A)** ELISA binding data for selected VRC01-class mAbs at increasing concentrations. Absorbance was measured at 450 nm. **(B)** Half-maximal binding titers (EC_50_s) of VRC01-class mAbs selected for structural characterization. **(C)** Half-maximal inhibitory titers (IC_50_s) of selected VRC01-class mAbs in a pseudovirus (PV) neutralization assay against the indicated PVs.


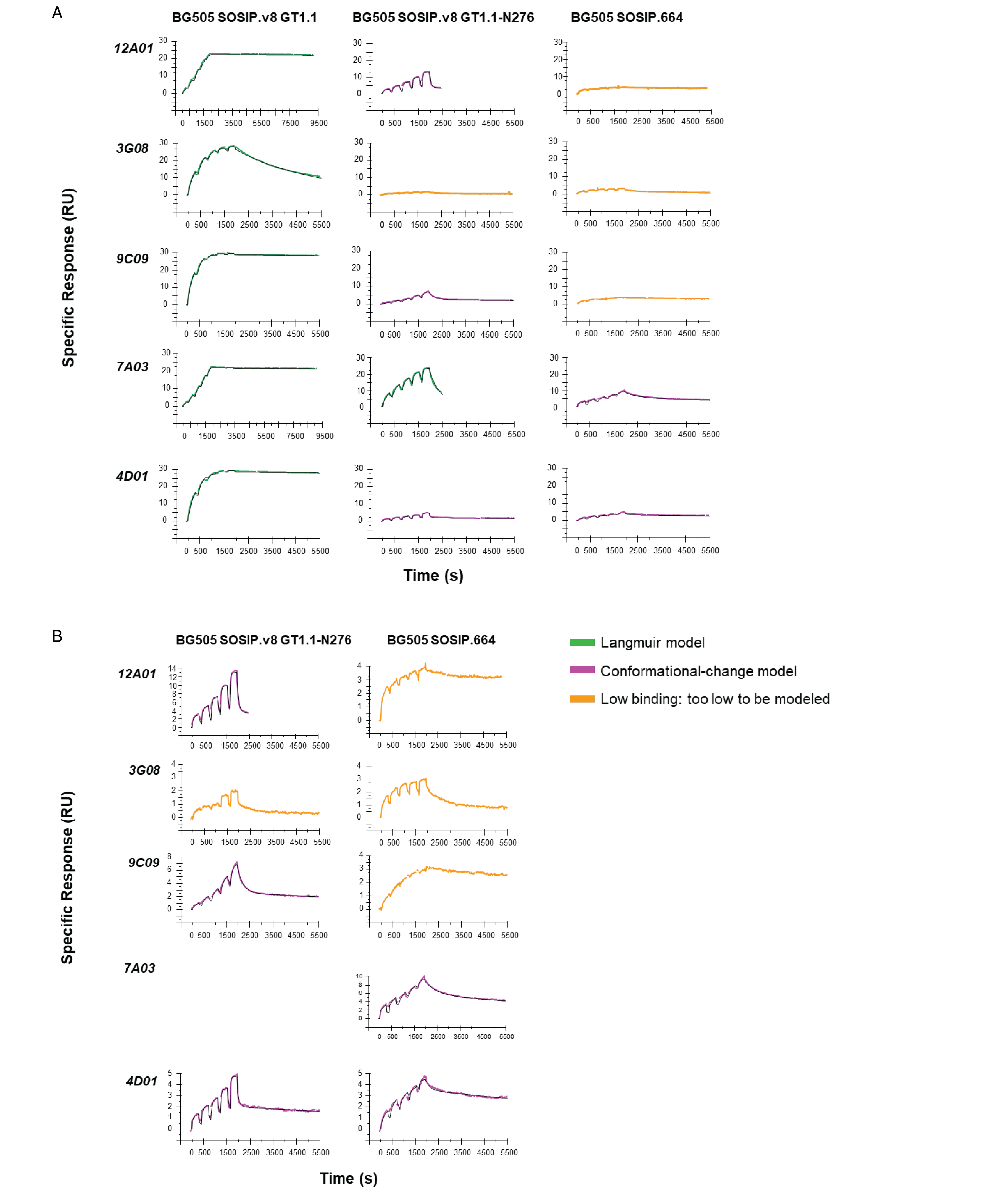


**Figure S32. Single-cycle kinetics (SCK) of mAbs binding to germline-adapted, N276-mutant, and non-adapted variants of HIV-1 BG505 Env trimers. (A)** Sensorgrams depict the binding response (RU) of mAbs on the y-axes, as function of time (s) along x-axes. Five concentrations of each mAb were injected in ascending order over 5 min of association, followed by dissociation for 1 h. In case of 12A01 and 7A03 *versus* GT1.1, dissociation was monitored for 2 h because of slower dissociation. For comparison, all sensorgrams have the same scale on the y-axes with maximum of 30 RU. The curves are color-coded for best fitting model (Langmuir or conformational-change model) or too low binding to model. The conformational-change model was validated by injection-time-variation test (tables S13-S14). **(B)** Detectable but too low binding to model for a subset of combinations from **(A)** is illustrated with y-axis adjusted to maximum binding.


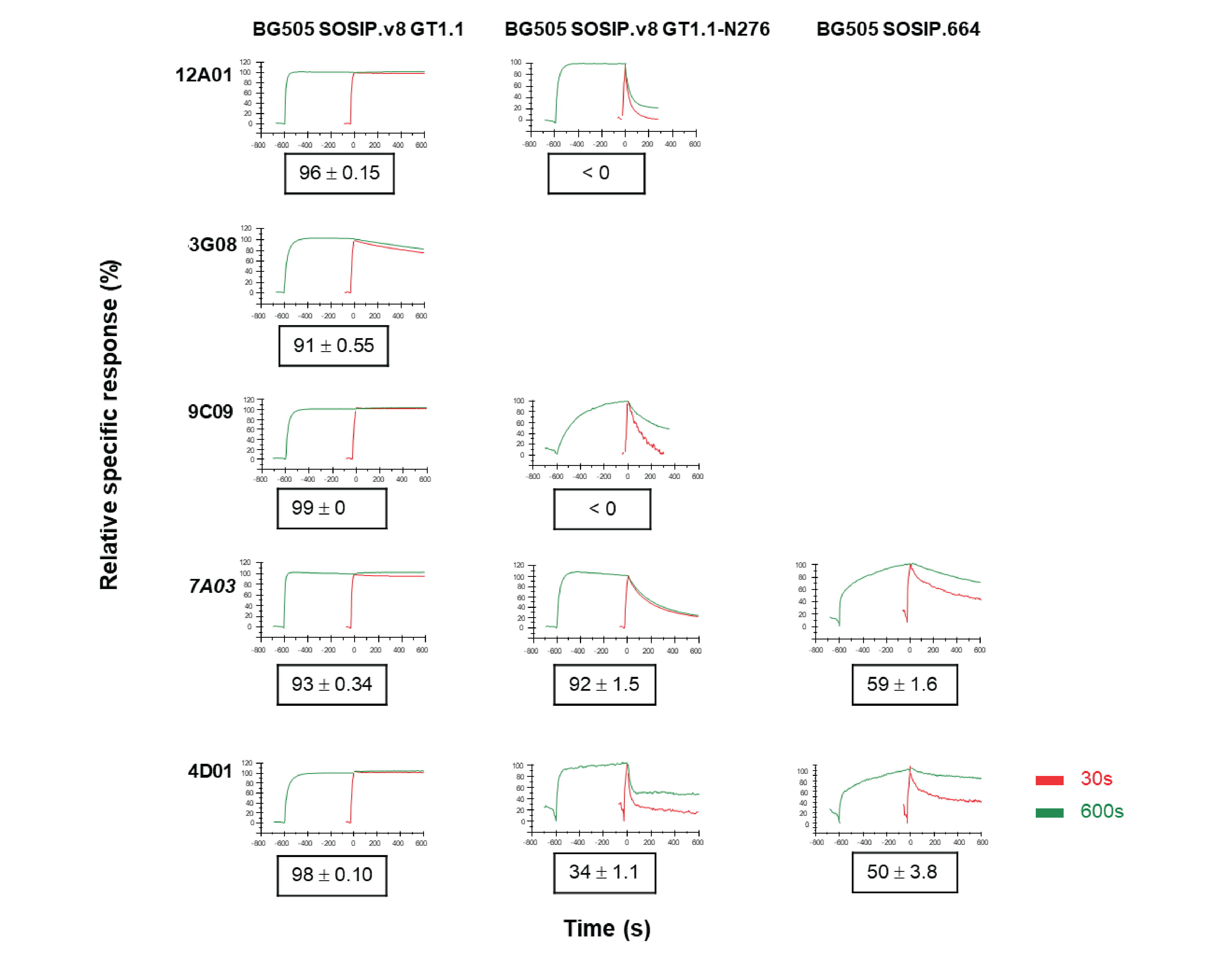


**Figure S33. Validation of conformation-change model by injection time variation test.** mAbs were injected as analyte with varying contact times (30 s and 600 s) to monitor the change in dissociation curve as a function of conformational-change. Sensorgrams were normalized and overlayed to calculate the relative residual binding for 30 s- *versus* 600 s-contact after 600 s subsequent dissociation expressed in %: the lower the percentage, the stronger is the evidence for real conformational-change. Residual binding near 100% indicated absence of conformational-change (observed for all mAbs against GT1.1 and 7A03 also with SOSIP.v8 GT1.1-N276). Means ± SEM of relative residual binding (%) of two replicates are given below each sensorgram. Combinations failing the injection-time-test, *i.e.*, *bona fide* Langmuir-fitting binding, are shown to contrast with the corroborated conformational changes.


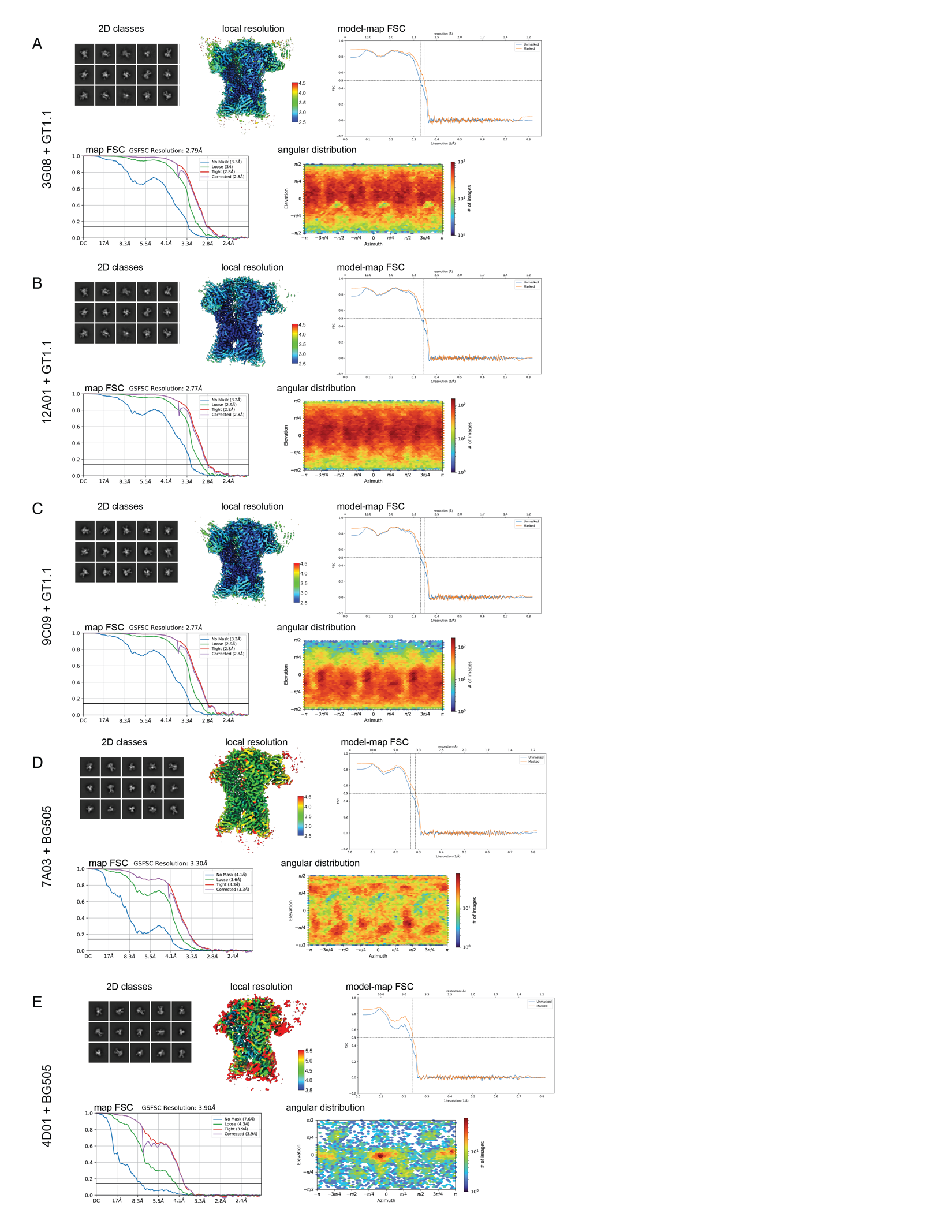


**Figure S34. Cryo-EM reconstruction statistics.** Representative 2D class averages, local resolution estimation (in units Å), atomic model to map Fourier shell correlation, Fourier shell correlation resolution estimate, and angular distribution of observations of **(A)** 3G08 Fab + GT1.1 SOSIP, **(B)** 12A01 + GT1.1 SOSIP, **(C)** 9C09 + GT1.1 SOSIP, **(D)** 7A03 + BG505 SOSIP and **(E)** 4D01 + BG505 SOSIP. See also table S15.

**
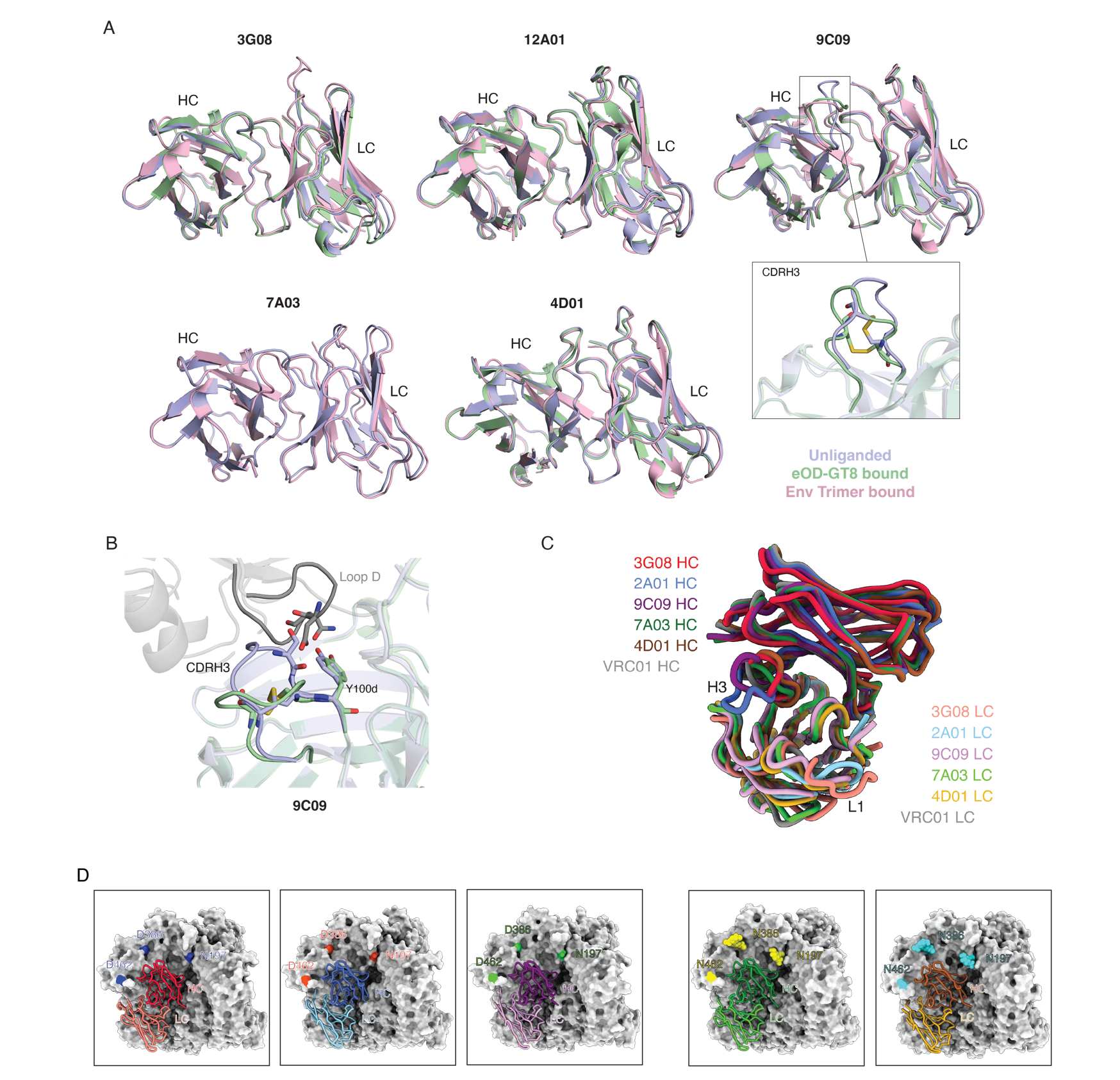
**

**Figure S35. Structural alignments of C101 VRC01-class antibodies.** **A)** Structural alignment of the Fv regions for the unliganded (blue), eOD-GT8-bound (green), and Env-trimer-bound (pink) forms of the listed antibodies, shown as a cartoon representation. The disulfide bond in the CDRH3 of 9C09 is shown in stick representation. **(B)** Structural comparison of the CDRH3 region of 9C09 in the unliganded and eOD-GT8-bound forms. The unliganded form shows a predicted clash with Loop D, while structural rearrangement of CDRH3 is required to accommodate binding. **(C)** Alignment of the Fv regions of five C101 mAbs to the VRC01 antibody, with respective colors as labeled. The positions of the CDRH3 and CDRL1 loops are indicated by “H3” and “L1,” respectively. **(D)** Relative positions of N197, N386 and N462 (N-linked glycans in BG505 but not GT1.1) are shown with respect to each mAb as in Fig. 5.


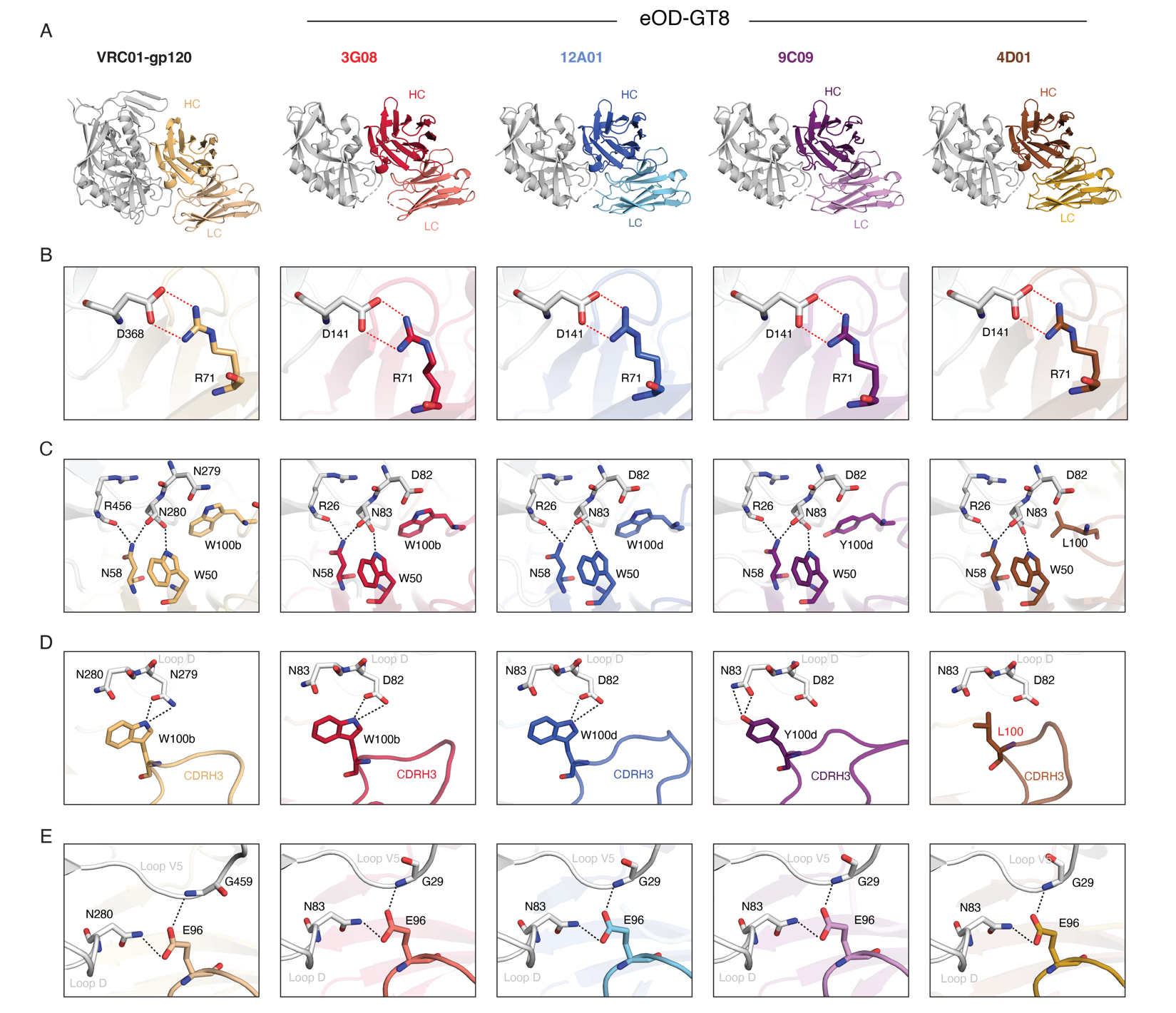


**Figure S36. Crystal structures of C101 VRC01-class antibodies bound to eOD-GT8. (A)** Structural comparison of Fabs from C101 VRC01-class antibodies bound to eOD-GT8 with the VRC01-gp120 complex (PDB ID: 3NGB). **(B)** Salt-bridge interactions (shown as red dashed lines) between R71_HC_ and D368_Env_, highlighting a key conserved feature of C101 VRC01-class antibodies in their interaction with eOD-GT8 and gp120. Antibody numbering follows the Kabat scheme. **(C)** Characteristic VRC01-like molecular interactions from IGHV1-2 germline-encoded residues W50 and N58 are shown for C101 VRC01-class antibodies with eOD-GT8. Hydrogen bonds depicted as black dashed lines. **(D)** Comparison of CDRH3 loops, showing the replacement of the conserved W103-5 residue in VRC01 with corresponding residues in C101 mAbs, and their interactions with loop D. **(E)** Comparison of CDRL3 loops, illustrating the conserved E96-mediated interactions, a hallmark of VRC01-class antibodies.


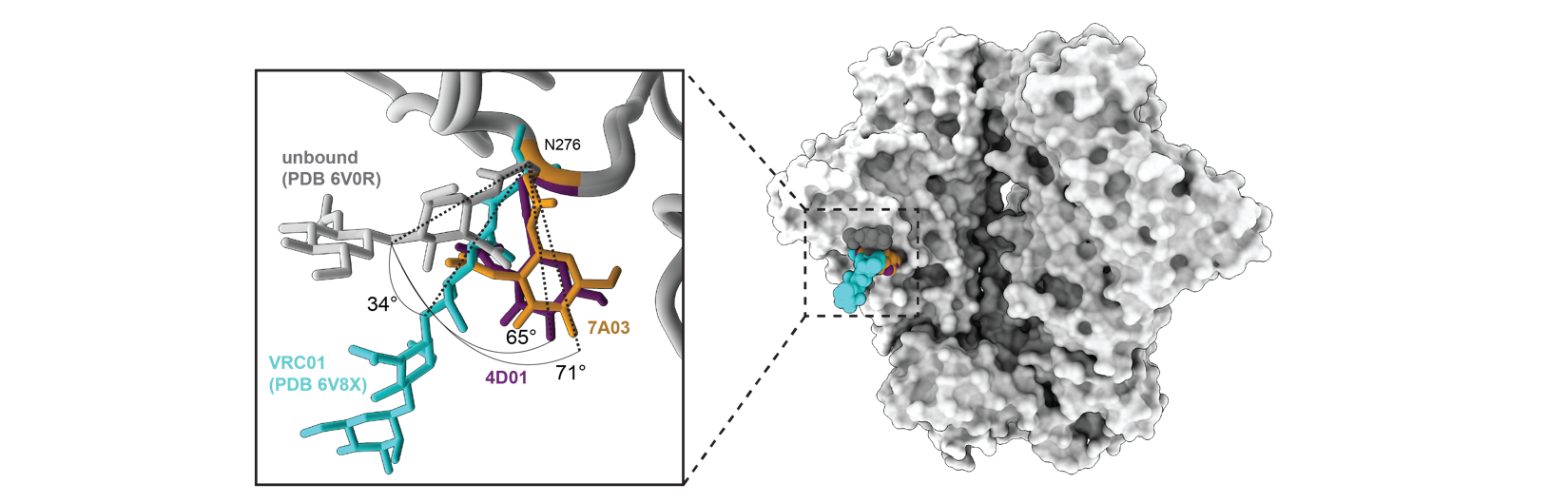


**Figure S37. Displacement of N276 glycan by VRC01-class antibodies**. Comparison of modeled N276 glycan atoms in the 7A03 (orange) and 4D01 (purple) Fab models (in complex with BG505 SOSIP) with VRC01 (PDB 6V8X, cyan) and unbound BG505 SOSIP (PDB 6V0R, gray). Inset summarizes the angle of displacement between the unbound N276 glycan and the glycans bound by antibodies from this study and VRC01. Angles were defined by the O4 atoms of the first NAG residue of each respective N276 glycan, and the Cα atom of residue N276.

**Table S1. Participant demographics.**

|  | **Overall** |  | **Placebo** |  | **Low Dose** | **High Dose** | **Overall** |
| --- | --- | --- | --- | --- | --- | --- | --- |
|  | **(N=47)** |  | **(N=8)** |  | **(N=20)** | **(N=19)** | **Vaccinees** |
|  | **n (%)** |  | **n (%)** |  | **n (%)** | **n (%)** | **(N=39)** |
|  |  |  |  |  |  |  | **n (%)** |
| **Sex at birth, n (%)** | | | | | | | |
| Female | 21 (44.7) |  | 5 (62.5) |  | 8 (40.0) | 8 (42.1) | 16 (41.0) |
| Male | 26 (55.3) |  | 3 (37.5) |  | 12 (60.0) | 11 (57.9) | 23 (59.0) |
|  |  |  |  |  |  |  |  |
| **Gender, n (%)** | | | | | | | |
| Female | 18 (38.3) |  | 3 (37.5) |  | 7 (35.0) | 8 (42.1) | 15 (38.5) |
| Male | 25 (53.2) |  | 3 (37.5) |  | 11 (55.0) | 11 (57.9) | 22 (56.4) |
| Transgender  Female | 1 (2.1) |  | 0 (0.0) |  | 1 (5.0) | 0 (0.0) | 1 (2.6) |
| Transgender Male | 2 (4.3) |  | 1 (12.5) |  | 1 (5.0) | 0 (0.0) | 1 (2.6) |
| Gender Variant or Non-Conforming | 1 (2.1) |  | 1 (12.5) |  | 0 (0.0) | 0 (0.0) | 0 (0.0) |
|  |  |  |  |  |  |  |  |
| **Ethnicity, n (%)** | | | | | | | |
| Not Hispanic and not Latino | 46 (97.9) |  | 7 (87.5) |  | 20 (100.0) | 19 (100.0) | 39 (100.0) |
| Hispanic or Latino | 1 (2.1) |  | 1 (12.5) |  | 0 (0.0) | 0 (0.0) | 0 (0.0) |
|  |  |  |  |  |  |  |  |
| **Race, n (%)** | | | | | | | |
| White | 30 (63.8) |  | 6 (75.0) |  | 12 (60.0) | 12 (63.2) | 24 (61.5) |
| Black or African American | 8 (17.0) |  | 1 (12.5) |  | 3 (15.0) | 4 (21.1) | 7 (17.9) |
| Asian | 3 (6.4) |  | 0 (0.0) |  | 2 (10.0) | 1 (5.3) | 3 (7.7) |
| Multiracial^1^ | 4 (8.5) |  | 1 (12.5) |  | 1 (5.0) | 2 (10.5) | 3 (7.7) |
| Other/Unknown | 2 (4.3) |  | 0 (0.0) |  | 2 (10.0) | 0 (0.0) | 2 (5.1) |
|  |  |  |  |  |  |  |  |
| **Age (years)** |  |  |  |  |  |  |  |
| Median | 30 |  | 30.5 |  | 29.5 | 29 | 29 |
| Range | 19 - 47 |  | 26 - 47 |  | 20 - 37 | 19 - 43 | 19 – 43 |
|  |  |  |  |  |  |  |  |
| **BMI (kg/m^2^)** |  |  |  |  |  |  |  |
| Median | 25.7 |  | 25.3 |  | 25.9 | 24.9 | 25.7 |
| Range | 18.9 - 34.8 |  | 21.0 - 33.8 |  | 18.9 - 34.8 | 19.3 - 32.3 | 18.9 - 34.8 |

N = Total number of volunteers in the randomized population within each dose level
n = Number of volunteers with non-missing data in each category
% = Percentage of volunteers in each category, i.e., 100 x n/N
^1^ Volunteers claiming to be of more than one race are classed as multiracial

**Table S2. Safety summary**

|  |  | **GT1.1 + AS01_B_** | | | |  | **Placebo** |
| --- | --- | --- | --- | --- | --- | --- | --- |
|  |  | **Low Dose** |  | **High Dose** | **All Vaccinees** |  |  |
|  |  | **(N=20)** |  | **(N=19)** | **(N=39)** |  | **(N=8)** |
|  |  | **n (%)** |  | **n (%)** | **n (%)** |  | **n (%)** |
| **Any AE^1,2,4^** |  | 19 (95) |  | 18 (95) | 37 (95) |  | 8 (100) |
|  |  |  |  |  |  |  |  |
| **Any Solicited AE^2,3^** |  | 19 (95) |  | 18 (95) | 37 (95) |  | 7 (88) |
| **Local Reactogenicity** |  | 19 (95) |  | 18 (95) | 37 (95) |  | 4 (50) |
| **Grade 2-4** |  | 15 (75) |  | 15 (79) | 30 (77) |  | 2 (25) |
| **Systemic Reactogenicity** |  | 18 (90) |  | 16 (84) | 34 (87) |  | 7 (88) |
| **Grade 2-4** |  | 13 (65) |  | 13 (68) | 26 (67) |  | 3 (38) |
|  |  |  |  |  |  |  |  |
| **Any Unsolicited AEs^4^** |  | 12 (60) |  | 13 (68) | 25 (64) |  | 8 (100) |
| **Related^5^** |  | 6 (30) |  | 6 (32) | 12 (31) |  | 3 (38) |
| **Grade 2-4** |  | 2 (10) |  | 6 (32) | 8 (21) |  | 1 (13) |
| **Related^5^ and Grade 2-4** |  | 0 (0) |  | 1 (5) | 1 (3) |  | 1 (13) |
|  |  |  |  |  |  |  |  |
| **Any SAEs** |  | 1 (5) |  | 0 (0) | 1 (3) |  | 0 (0) |
| **Related^5^** |  | 0 (0) |  | 0 (0) | 0 (0) |  | 0 (0) |
| **Any pIMDs** |  | 0 (0) |  | 0 (0) | 0 (0) |  | 0 (0) |
| **Related^5^** |  | 0 (0) |  | 0 (0) | 0 (0) |  | 0 (0) |
|  |  |  |  |  |  |  |  |
| **AE Leading to Treatment Discontinuation** |  | 0 (0) |  | 0 (0) | 0 (0) |  | 0 (0) |
| **AE Leading to Study Discontinuation** |  | 0 (0) |  | 0 (0) | 0 (0) |  | 0 (0) |
|  |  |  |  |  |  |  |  |
| **Any Grade 2-4 Laboratory Result^6^** |  | 0 (0) |  | 0 (0) | 0 (0) |  | 0 (0) |

N = Total number of volunteers in the safety analysis population in each dose level.
n = Number of volunteers who experienced at least one event (volunteers with >1 reported event are counted only once in each category).
% = Percentage of volunteers in each category relative to the total number within each study group.
95% CI = Exact, Clopper-Pearson 95% confidence interval for the percent.
SAE = Serious adverse event reported at any time during the study.
pIMD = Potential immune-mediated disease reported at any time during the study.

^1^ Includes solicited AEs and unsolicited AEs.
^2^ Solicited AEs are reported through 7 days post-administration (i.e., 8-day follow-up period).
^3^ By definition, solicited AEs (local and systemic reactogenicity) are considered related to IP.
^4^ Unsolicited AEs are reported through 28 days after each IP administration. Includes SAEs.
^4^ "Related" is defined as possibly, probably, or definitely related to IP.
^5^ Chemistry or hematology laboratory results post-IP administration and through 28 days after each administration.

**Table S3. Reported reactogenicity adverse events.**

|  |  | **GT1.1 + AS01_B_** | | |  | **Placebo** |
| --- | --- | --- | --- | --- | --- | --- |
| **Severity Grade** |  | **Low Dose** | **High Dose** | **All Vaccinees** |  |  |
|  |  | **(N=20)** | **(N=19)** | **(N=39)** |  | **(N=8)** |
|  |  | **n (%)** | **n (%)** | **n (%)** |  | **n (%)** |
| **Any Reactogenicity AE** | | | | |  |  |
| Any |  | 19 (95) | 18 (95) | 37 (95) |  | 7 (88) |
| Grade 1 |  | 1 (5) | 1 (5) | 2 (5) |  | 4 (50) |
| Grade 2 |  | 11 (55) | 15 (79) | 26 (67) |  | 3 (38) |
| Grade 3 |  | 7 (35) | 2 (11) | 9 (23) |  | 0 (0) |
|  |  |  |  |  |  |  |
| **Any Local Reactogenicity** | | | | |  |  |
| Any |  | 19 (95) | 18 (95) | 37 (95) |  | 4 (50) |
| Grade 1 |  | 4 (20) | 3 (16) | 7 (18) |  | 2 (25) |
| Grade 2 |  | 12 (60) | 14 (74) | 26 (67) |  | 2 (25) |
| Grade 3 |  | 3 (15) | 1 (5) | 4 (10) |  | 0 (0) |
| **Tenderness** | | | | |  |  |
| Any |  | 19 (95) | 18 (95) | 37 (95) |  | 3 (38) |
| Grade 1 |  | 6 (30) | 6 (32) | 12 (31) |  | 2 (25) |
| Grade 2 |  | 13 (65) | 11 (58) | 24 (62) |  | 1 (13) |
| Grade 3 |  | 0 (0) | 1 (5) | 1 (3) |  | 0 (0) |
| **Pain** | | | | |  |  |
| Any |  | 18 (90) | 17 (90) | 35 (90) |  | 3 (38) |
| Grade 1 |  | 7 (35) | 6 (32) | 13 (33) |  | 1 (13) |
| Grade 2 |  | 11 (55) | 10 (53) | 21 (54) |  | 2 (25) |
| Grade 3 |  | 0 (0) | 1 (5) | 1 (3) |  | 0 (0) |
| **Erythema** | | | | |  |  |
| Any |  | 6 (30) | 3 (16) | 9 (23) |  | 0 (0) |
| Grade 1 |  | 2 (10) | 0 (0) | 2 (5) |  | 0 (0) |
| Grade 2 |  | 4 (20) | 3 (16) | 7 (18) |  | 0 (0) |
| Grade 3 |  | 0 (0) | 0 (0) | 0 (0) |  | 0 (0) |
| **Swelling** | | | | |  |  |
| Any |  | 5 (25) | 2 (11) | 7 (18) |  | 0 (0) |
| Grade 1 |  | 1 (5) | 0 (0) | 1 (3) |  | 0 (0) |
| Grade 2 |  | 1 (5) | 2 (11) | 3 (8) |  | 0 (0) |
| Grade 3 |  | 3 (15) | 0 (0) | 3 (8) |  | 0 (0) |
|  |  |  |  |  |  |  |
| **Any Systemic Reactogenicity** | | | | |  |  |
| Any |  | 18 (90) | 16 (84) | 34 (87) |  | 7 (88) |
| Grade 1 |  | 5 (25) | 3 (16) | 8 (21) |  | 4 (50) |
| Grade 2 |  | 9 (45) | 11 (58) | 20 (51) |  | 3 (38) |
| Grade 3 |  | 4 (20) | 2 (11) | 6 (15) |  | 0 (0) |
| **Malaise** | | | | |  |  |
| Any |  | 17 (85) | 13 (68) | 30 (77) |  | 3 (38) |
| Grade 1 |  | 6 (30) | 5 (26) | 11 (28) |  | 2 (25) |
| Grade 2 |  | 9 (45) | 6 (32) | 15 (39) |  | 1 (13) |
| Grade 3 |  | 2 (10) | 2 (11) | 4 (10) |  | 0 (0) |
| **Headache** | | | | |  |  |
| Any |  | 14 (70) | 14 (74) | 28 (72) |  | 5 (63) |
| Grade 1 |  | 6 (30) | 4 (21) | 10 (26) |  | 5 (63) |
| Grade 2 |  | 7 (35) | 10 (53) | 17 (44) |  | 0 (0) |
| Grade 3 |  | 1 (5) | 0 (0) | 1 (3) |  | 0 (0) |
| **Myalgia** | | | | |  |  |
| Any |  | 16 (80) | 12 (63) | 28 (72) |  | 2 (25) |
| Grade 1 |  | 6 (30) | 3 (16) | 9 (23) |  | 1 (13) |
| Grade 2 |  | 10 (50) | 8 (42) | 18 (46) |  | 1 (13) |
| Grade 3 |  | 0 (0) | 1 (5) | 1 (3) |  | 0 (0) |
| **Chills** | | | | |  |  |
| Any |  | 15 (75) | 8 (42) | 23 (59) |  | 0 (0) |
| Grade 1 |  | 5 (25) | 1 (5) | 6 (15) |  | 0 (0) |
| Grade 2 |  | 8 (40) | 5 (26) | 13 (33) |  | 0 (0) |
| Grade 3 |  | 2 (10) | 2 (11) | 4 (10) |  | 0 (0) |
| **Nausea** | | | | |  |  |
| Any |  | 10 (50) | 8 (42) | 18 (46) |  | 1 (13) |
| Grade 1 |  | 9 (45) | 2 (11) | 11 (28) |  | 1 (13) |
| Grade 2 |  | 1 (5) | 5 (26) | 6 (15) |  | 0 (0) |
| Grade 3 |  | 0 (0) | 1 (5) | 1 (3) |  | 0 (0) |
| **Arthralgia** | | | | |  |  |
| Any |  | 10 (50) | 7 (37) | 17 (44) |  | 2 (25) |
| Grade 1 |  | 7 (35) | 3 (16) | 10 (26) |  | 1 (13) |
| Grade 2 |  | 3 (15) | 3 (16) | 6 (15) |  | 1 (13) |
| Grade 3 |  | 0 (0) | 1 (5) | 1 (3) |  | 0 (0) |
| **Abdominal Pain** | | | | |  |  |
| Any |  | 5 (25) | 6 (32) | 11 (28) |  | 3 (38) |
| Grade 1 |  | 3 (15) | 3 (16) | 6 (15) |  | 2 (25) |
| Grade 2 |  | 2 (10) | 3 (16) | 5 (13) |  | 1 (13) |
| Grade 3 |  | 0 (0) | 0 (0) | 0 (0) |  | 0 (0) |
| **Diarrhea** | | | | |  |  |
| Any |  | 2 (10) | 7 (37) | 9 (23) |  | 2 (25) |
| Grade 1 |  | 2 (10) | 6 (32) | 8 (21) |  | 2 (25) |
| Grade 2 |  | 0 (0) | 1 (5) | 1 (3) |  | 0 (0) |
| Grade 3 |  | 0 (0) | 0 (0) | 0 (0) |  | 0 (0) |
| **Fever** | | | | |  |  |
| Any |  | 4 (20) | 1 (5) | 5 (13) |  | 1 (13) |
| Grade 1 |  | 3 (15) | 0 (0) | 3 (8) |  | 0 (0) |
| Grade 2 |  | 0 (0) | 1 (5) | 1 (3) |  | 1 (13) |
| Grade 3 |  | 1 (5) | 0 (0) | 1 (3) |  | 0 (0) |
| **Vomiting** | | | | |  |  |
| Any |  | 1 (5) | 1 (5) | 2 (5) |  | 0 (0) |
| Grade 1 |  | 1 (5) | 0 (0) | 1 (3) |  | 0 (0) |
| Grade 2 |  | 0 (0) | 1 (5) | 1 (3) |  | 0 (0) |
| Grade 3 |  | 0 (0) | 0 (0) | 0 (0) |  | 0 (0) |
| N = Total number of volunteers in the safety analysis population. | | | | | | |
| n (%) = Number of volunteers with an AE (% of N). | | | | |  |  |
| The maximum reported severity over all events was Grade 3. | | | | |  |  |

**Table S4. Related non-reactogenicity events.**

|  |  |  |  | **GT1.1 + AS01_B_** | | |  | | **Placebo** | |
| --- | --- | --- | --- | --- | --- | --- | --- | --- | --- | --- |
| **Adverse Event** |  | **Severity Grade** |  | **Low Dose** | **High Dose** | **All Vaccinees** |  | |  | |
|  |  |  |  | **(n=20)** | **(n=19)** | **(n=39)** |  | | **(n=8)** | |
|  |  |  |  | **n (%)** | **n (%)** | **n (%)** |  | | **n (%)** | |
| Any Non-reactogenicity AE |  | Any |  | 6 (30) | 6 (32) | 12 (31) |  | | 3 (38) | |
|  |  | Grade 1 |  | 6 (30) | 5 (26) | 11 (28) |  | | 2 (25) | |
|  |  | Grade 2 |  | 0 (0) | 1 (5) | 1 (3) |  | | 1 (13) | |
| Fatigue |  | Any |  | 0 (0) | 1 (5) | 1 (3) |  | | 2 (25) | |
|  |  | Grade 1 |  | 0 (0) | 0 (0) | 0 (0) |  | | 1 (13) | |
|  |  | Grade 2 |  | 0 (0) | 1 (5) | 1 (3) |  | | 1 (13) | |
| Injection site pruritus |  | Any |  | 1 (5) | 1 (5) | 2 (5) |  | | 0 (0) | |
|  |  | Grade 1 |  | 1 (5) | 1 (5) | 2 (5) |  | | 0 (0) | |
|  |  | Grade 2 |  | 0 (0) | 0 (0) | 0 (0) |  | | 0 (0) | |
| Injection site bruising |  | Any |  | 1 (5) | 0 (0) | 1 (3) |  | | 0 (0) | |
|  |  | Grade 1 |  | 1 (5) | 0 (0) | 1 (3) |  | | 0 (0) | |
|  |  | Grade 2 |  | 0 (0) | 0 (0) | 0 (0) |  | | 0 (0) | |
| Injection site erythema |  | Any |  | 1 (5) | 0 (0) | 1 (3) |  | | 0 (0) | |
|  |  | Grade 1 |  | 1 (5) | 0 (0) | 1 (3) |  | | 0 (0) | |
|  |  | Grade 2 |  | 0 (0) | 0 (0) | 0 (0) |  | | 0 (0) | |
| Injection site swelling |  | Any |  | 1 (5) | 0 (0) | 1 (3) |  | | 0 (0) | |
|  |  | Grade 1 |  | 1 (5) | 0 (0) | 1 (3) |  | | 0 (0) | |
|  |  | Grade 2 |  | 0 (0) | 0 (0) | 0 (0) |  | | 0 (0) | |
| Swelling |  | Any |  | 1 (5) | 0 (0) | 1 (3) |  | | 0 (0) | |
|  |  | Grade 1 |  | 1 (5) | 0 (0) | 1 (3) |  | | 0 (0) | |
|  |  | Grade 2 |  | 0 (0) | 0 (0) | 0 (0) |  | | 0 (0) | |
| Dizziness |  | Any |  | 1 (5) | 3 (16) | 4 (10) |  | | 1 (13) | |
|  |  | Grade 1 |  | 1 (5) | 3 (16) | 4 (10) |  | | 1 (13) | |
|  |  | Grade 2 |  | 0 (0) | 0 (0) | 0 (0) |  | | 0 (0) | |
| Hypoaesthesia |  | Any |  | 1 (5) | 0 (0) | 1 (3) |  | | 0 (0) | |
|  |  | Grade 1 |  | 1 (5) | 0 (0) | 1 (3) |  | | 0 (0) | |
|  |  | Grade 2 |  | 0 (0) | 0 (0) | 0 (0) |  | | 0 (0) | |
| Tremor |  | Any |  | 0 (0) | 1 (5) | 1 (3) |  | | 0 (0) | |
|  |  | Grade 1 |  | 0 (0) | 1 (5) | 1 (3) |  | | 0 (0) | |
|  |  | Grade 2 |  | 0 (0) | 0 (0) | 0 (0) |  | | 0 (0) | |
| Lymph node pain |  | Any |  | 0 (0) | 1 (5) | 1 (3) |  | | 0 (0) | |
|  |  | Grade 1 |  | 0 (0) | 1 (5) | 1 (3) |  | | 0 (0) | |
|  |  | Grade 2 |  | 0 (0) | 0 (0) | 0 (0) |  | | 0 (0) | |
| Constipation |  | Any |  | 0 (0) | 1 (5) | 1 (3) |  | | 0 (0) | |
|  |  | Grade 1 |  | 0 (0) | 1 (5) | 1 (3) |  | | 0 (0) | |
|  |  | Grade 2 |  | 0 (0) | 0 (0) | 0 (0) |  | | 0 (0) | |
| Gingivitis |  | Any |  | 0 (0) | 1 (5) | 1 (3) |  | | 0 (0) | |
|  |  | Grade 1 |  | 0 (0) | 1 (5) | 1 (3) |  | | 0 (0) | |
|  |  | Grade 2 |  | 0 (0) | 0 (0) | 0 (0) |  | | 0 (0) | |
| Mouth injury |  | Any |  | 1 (5) | 0 (0) | 1 (3) |  | | 0 (0) | |
|  |  | Grade 1 |  | 1 (5) | 0 (0) | 1 (3) |  | | 0 (0) | |
|  |  | Grade 2 |  | 0 (0) | 0 (0) | 0 (0) |  | | 0 (0) | |
| Neck pain |  | Any |  | 1 (5) | 0 (0) | 1 (3) |  | | 0 (0) | |
|  |  | Grade 1 |  | 1 (5) | 0 (0) | 1 (3) |  | | 0 (0) | |
|  |  | Grade 2 |  | 0 (0) | 0 (0) | 0 (0) |  | | 0 (0) | |
| Oropharyngeal pain |  | Any |  | 0 (0) | 0 (0) | 0 (0) |  | | 1 (13) | |
|  |  | Grade 1 |  | 0 (0) | 0 (0) | 0 (0) |  | | 1 (13) | |
|  |  | Grade 2 |  | 0 (0) | 0 (0) | 0 (0) |  | | 0 (0) | |
| Pruritus |  | Any |  | 1 (5) | 0 (0) | 1 (3) |  | | 0 (0) | |
|  |  | Grade 1 |  | 1 (5) | 0 (0) | 1 (3) |  | | 0 (0) | |
|  |  | Grade 2 |  | 0 (0) | 0 (0) | 0 (0) |  | | 0 (0) | |
| N = Total number of volunteers in the safety analysis population. | | | | | | | |  | |  |
| n (%) = Number of volunteers with an event (% of N) | | | | | | | |  | |  |
| The maximum reported severity over all events was Grade 2. | | | | | | | |  | |  |

**Table S5. IAVI C101 schedule of procedures.**

**(continued) Table S5.** **IAVI C101 schedule of procedures.**

**Table S6. IAVI C101 fluorophore panel for flow cytometry.**

| **Probe or antibody** | **Fluorophore** | **Clone** | **Vendor** | **Cat** |
| --- | --- | --- | --- | --- |
| IgM | BB700 | G20-127 | custom | N/A |
| CD11c | BB790 | bly6 | custom | N/A |
| CD21 | PE594 | bly4 | BD | 563474 |
| CD85J | PeCy7 | GH1/75 | Biolegend | 333712 |
| CD20 | APC/Fire | 2H7 | Biolegend | 302358 |
| IgG | BUV395 | G18-145 | BD | 564229 |
| Viability | N/A | N/A | Invitrogen | L34962 |
| CD38 | BUV661 | HIT2 | BD | 612969 |
| CD62L | BUV805 | DREG-56 | BD |  |
| CD3 | BV510 | OKT3 | Biolegend | 317332 |
| CD14 | BV510 | NCAM16.2 | Biolegend | 301842 |
| CD56 | BV510 | M5E2 | Biolegend | 563041 |
| CD16 | BV511 | 3G8 | Biolegend | 302048 |
| IgD | BV570 | IA6-2 | BD | Custom |
| CD27 | BV605 | O323 | Biolegend | 302830 |
| CD71 | BV650 | CY1G4 | Biolegend | 334116 |
| CD19 | BV750 | SJ25C1 | BD | 747161 |
| GT1.1 super-KO probe | Ax488 | N/A | N/A | N/A |
| GT1.1 apex-KO probe | BV786 | N/A | N/A | N/A |
| GT1.1 probe | Ax647 | N/A | N/A | N/A |
| GT1.1 probe | PE | N/A | N/A | N/A |
| GT1.1 CD4bs-KO probe | BV711 | N/A | N/A | N/A |

**Table S7. Numbers of processed PBMCs per dose and time point (millions).**

|  |  | **Week** |  |
| --- | --- | --- | --- |
|  | **-4** | **10** | **26** |
| **High dose** | 300 | 300 | 200 |
| **Low dose** | 300 | 300 | 200 |

**Table S8. Frequency of key cell populations in total IgG memory B cells.**

|  |  |  | **Frequency of cell population in IgG memory B cells (%)** | | | | |
| --- | --- | --- | --- | --- | --- | --- | --- |
| Cell population | Study timepoint | Group | Median | Mean | Minimum | Maximum | 1 in |
| GT1.1++ | week -4 | p | 0.00283746 | 0.01126684 | 0.00067114 | 0.06430318 | 35243 |
| GT1.1++ | week -4 | LD | 0.00223325 | 0.00761731 | 0.00048544 | 0.06944444 | 44778 |
| GT1.1++ | week -4 | HD | 0.00323834 | 0.0055125 | 0.00072115 | 0.0293609 | 30880 |
| GT1.1++ | week 10 | p | 0.00518591 | 0.01033589 | 0.0012987 | 0.0518315 | 19283 |
| GT1.1++ | week 10 | LD | 1.43725664 | 2.11628418 | 0.1651145 | 12.5032787 | 70 |
| GT1.1++ | week 10 | HD | 1.72916158 | 2.05096143 | 0.3917753 | 4.59452555 | 58 |
| GT1.1++ | week 26 | p | 0.00427014 | 0.013307 | 0.00042965 | 0.07118088 | 23418 |
| GT1.1++ | week 26 | LD | 1.73029678 | 1.86018625 | 0.72754286 | 3.45571809 | 58 |
| GT1.1++ | week 26 | HD | 3.16322242 | 3.44189538 | 1.1697191 | 7.84232491 | 32 |
| CD4bs+ | week -4 | p | 0.00017369 | 0.00017369 | 0.00010288 | 0.0002445 | 575739 |
| CD4bs+ | week -4 | LD | 0.00013514 | 0.00037185 | 0.00012077 | 0.00089286 | 740000 |
| CD4bs+ | week -4 | HD | 0.00012953 | 0.00017149 | 0.00011261 | 0.00036496 | 772000 |
| CD4bs+ | week 10 | p | 0.00025957 | 0.00026538 | 0.00017271 | 0.0003663 | 385246 |
| CD4bs+ | week 10 | LD | 0.00389474 | 0.00434838 | 0.00084926 | 0.01064815 | 25676 |
| CD4bs+ | week 10 | HD | 0.006168 | 0.00959298 | 0.00059829 | 0.04806771 | 16213 |
| CD4bs+ | week 26 | p | 0.00032652 | 0.00034358 | 0.00030864 | 0.00039559 | 306262 |
| CD4bs+ | week 26 | LD | 0.01365129 | 0.03283524 | 8.00E-04 | 0.16934174 | 7325 |
| CD4bs+ | week 26 | HD | 0.11961471 | 0.12127175 | 0.0079191 | 0.34659418 | 836 |
| Apex+ | week -4 | p | 0.00055825 | 0.00055825 | 0.0001385 | 0.000978 | 179131 |
| Apex+ | week -4 | LD | 0.00015576 | 0.00051167 | 0.00012407 | 0.00240741 | 642000 |
| Apex+ | week -4 | HD | 0.00033059 | 0.0004568 | 0.00011001 | 0.00123874 | 302494 |
| Apex+ | week 10 | p | 0.00044714 | 0.0004508 | 0.00025957 | 0.00064935 | 223644 |
| Apex+ | week 10 | LD | 0.00112125 | 0.00737605 | 0.00010989 | 0.08330849 | 89186 |
| Apex+ | week 10 | HD | 0.01010789 | 0.01348707 | 0.00153333 | 0.0649635 | 9893 |
| Apex+ | week 26 | p | 0.00031009 | 0.00057453 | 0.00010741 | 0.00130607 | 322482 |
| Apex+ | week 26 | LD | 0.00161905 | 0.00188072 | 0.00028102 | 0.00505659 | 61765 |
| Apex+ | week 26 | HD | 0.04017279 | 0.04622544 | 0.0122746 | 0.138041 | 2489 |
| Epitope-unclassified | week -4 | p | 0.00283746 | 0.01086981 | 0.00060403 | 0.06234719 | 35243 |
| Epitope-unclassified | week -4 | LD | 0.00212054 | 0.00718811 | 0.00038835 | 0.06694444 | 47158 |
| Epitope-unclassified | week -4 | HD | 0.0027027 | 0.00489028 | 0.00072115 | 0.02774766 | 37000 |
| Epitope-unclassified | week 10 | p | 0.00475362 | 0.00948674 | 0.00048701 | 0.0507326 | 21037 |
| Epitope-unclassified | week 10 | LD | 0.96530973 | 1.14310275 | 0.13671756 | 5.80044709 | 104 |
| Epitope-unclassified | week 10 | HD | 0.90969773 | 0.93173952 | 0.22724261 | 2.28467153 | 110 |
| Epitope-unclassified | week 26 | p | 0.00373933 | 0.01275625 | 0.00032223 | 0.0695483 | 26743 |
| Epitope-unclassified | week 26 | LD | 0.94758915 | 1.0107016 | 0.2158237 | 1.74426667 | 106 |
| Epitope-unclassified | week 26 | HD | 1.30020683 | 1.32290559 | 0.61011236 | 2.90209472 | 77 |

**Table S9. Frequency of key cell populations in total IgD naive B cells.**

|  |  |  | **Frequency of cell population in IgD naive B cells (%)** | | | | |
| --- | --- | --- | --- | --- | --- | --- | --- |
| Cell population | Study timepoint | Group | Median | Mean | Minimum | Maximum | 1 in |
| GT1.1++ | week -4 | p | 0.000878043 | 0.00851338 | 0.00033163 | 0.06050485 | 113890 |
| GT1.1++ | week -4 | LD | 0.00172 | 0.00687246 | 0.00030172 | 0.0780914 | 58140 |
| GT1.1++ | week -4 | HD | 0.000747126 | 0.00091876 | 0.00028696 | 0.00217765 | 133846 |
| GT1.1++ | week 10 | p | 0.001095464 | 0.00783641 | 0.0004401 | 0.04918058 | 91286 |
| GT1.1++ | week 10 | LD | 0.015415861 | 0.02135797 | 0.00541379 | 0.09624339 | 6487 |
| GT1.1++ | week 10 | HD | 0.013643844 | 0.01698033 | 0.0011828 | 0.05485342 | 7329 |
| GT1.1++ | week 26 | p | 0.001575875 | 0.01256813 | 0.00052778 | 0.09033113 | 63457 |
| GT1.1++ | week 26 | LD | 0.005965299 | 0.01499947 | 0.00218579 | 0.1225969 | 16764 |
| GT1.1++ | week 26 | HD | 0.005062762 | 0.00707229 | 0.00193384 | 0.02348276 | 19752 |
| CD4bs+ | week -4 | p | 0.000102249 | 0.00010225 | 0.00010225 | 0.00010225 | 978000 |
| CD4bs+ | week -4 | LD | 0.000148649 | 0.00025378 | 0.00010283 | 0.00047511 | 672727 |
| CD4bs+ | week 10 | LD | 0.000232558 | 0.00027526 | 0.00013228 | 0.00056391 | 430000 |
| CD4bs+ | week 26 | p | 0.000172959 | 0.00017296 | 0.00010309 | 0.00024283 | 578171 |
| CD4bs+ | week 26 | LD | 0.000245098 | 0.00024154 | 0.00010545 | 0.00042821 | 408000 |
| CD4bs+ | week 26 | HD | 0.000167013 | 0.00016701 | 0.00015385 | 0.00018018 | 598755 |
| Apex+ | week -4 | p | 0.000102249 | 0.00010225 | 0.00010225 | 0.00010225 | 978000 |
| Apex+ | week -4 | LD | 0.000340845 | 0.00034084 | 0.00010753 | 0.00057416 | 293389 |
| Apex+ | week 10 | LD | 0.000141343 | 0.00014134 | 0.00014134 | 0.00014134 | 707500 |
| Apex+ | week 26 | LD | 0.000198246 | 0.00019825 | 0.00016393 | 0.00023256 | 504423 |
| Epitope-unclassified | week -4 | p | 0.00077883 | 0.00836951 | 0.00020918 | 0.06019417 | 128398 |
| Epitope-unclassified | week -4 | LD | 0.001311054 | 0.00656046 | 0.0002069 | 0.07733871 | 76275 |
| Epitope-unclassified | week -4 | HD | 0.000533333 | 0.00078833 | 0.00027826 | 0.00213467 | 187500 |
| Epitope-unclassified | week 10 | p | 0.000888166 | 0.00765915 | 0.00025974 | 0.04899848 | 112592 |
| Epitope-unclassified | week 10 | LD | 0.012862669 | 0.01774902 | 0.00410345 | 0.09513228 | 7774 |
| Epitope-unclassified | week 10 | HD | 0.009984844 | 0.01222746 | 0.00083333 | 0.04589577 | 10015 |
| Epitope-unclassified | week 26 | p | 0.001330977 | 0.01238355 | 0.00037963 | 0.08988962 | 75133 |
| Epitope-unclassified | week 26 | LD | 0.004245946 | 0.01396706 | 0.00191257 | 0.12151163 | 23552 |
| Epitope-unclassified | week 26 | HD | 0.004204204 | 0.00518945 | 0.0013486 | 0.01532759 | 23786 |

**Table S10. IAVI C101 primers to amplify BCR genes.**

| **Primer name** | **Sequence** |
| --- | --- |
| TSoligo2_polydT | CAAGCAGAAGACGGCATACGAGTTTTTTTTTTTTTTTTTTTTTTTTTTTTTTVN |
| A-tag TSO | /5Me-isodC//iisodG//iMe-isodC/AAGCAGTGGTATCAACGCAGAGTACATrGrGrG |
| TSoligo2 | CAAGCAGAAGACGGCATACGAG |
| TSO_FWD | AAGCAGTGGTATCAACGCAGAGT |
| IgG_REV | GCCAGGGGGAAGACCGATGGGCCCTTGGTGGA |
| IgK_REV | GCGGGAAGATGAAGACAGATGGTGCAGCCACAG |
| IgL_REV | GGCCTTGTTGGCTTGAAGCTCCTCAGAGGAGGG |
| IgM_REV | AGGAGACGAGGGGGAAAAGGGTTGGGGCGGATG |

**Table S11. IGHV alleles per participant and study group.** Fractions in parentheses show number of VRC01-class B cells detected over number of CD4bs+ B cells sequenced. Responders are highlighted in yellow, and genotypes non-compatible with the definition of a VRC01-class response are highlighted in red.

**
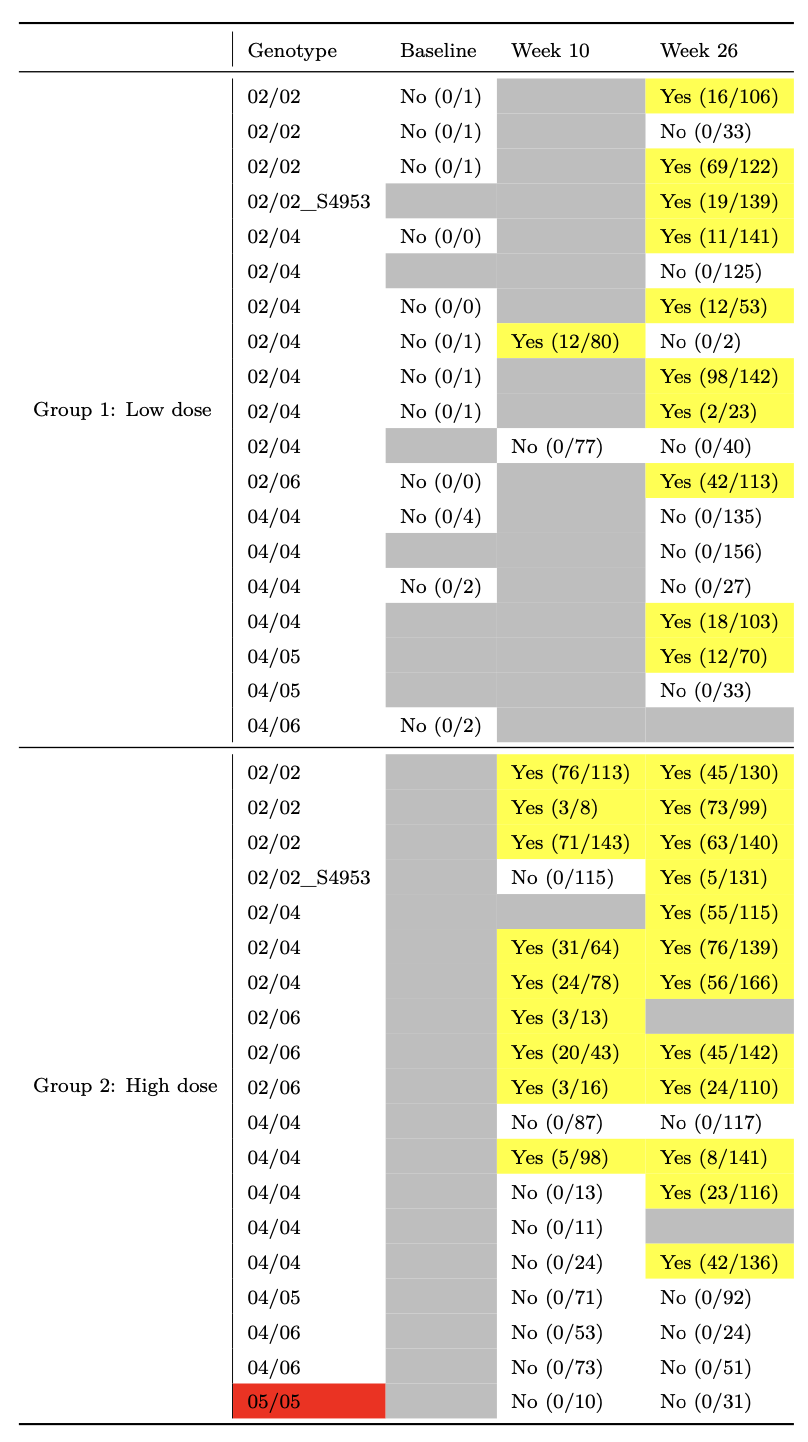
 Table S12. mAb binding to germline-adapted, N276-mutant, and non-adapted variants of HIV-1 BG505 Env trimers.**

1. **Langmuir model**

| **mAbs** | **HIV-1 BG505 Env trimer** | ***k_on_***  (1/Ms) | ***k_off_***  (1/s) | ***K_D_***  (nM) | ***S_m_*** |
| --- | --- | --- | --- | --- | --- |
| **12A01** | SOSIP.v8 GT1.1 | 7.3 **^.^** 10^5^  ± 2.3 **^.^** 10^3^ *^a^* | 3.3 **^.^** 10^-6^  ± 5.0 **^.^** 10^-8^ | 4.5 **^.^** 10^-3^  ± 8.4 **^.^** 10^-5^ | 2.6  ± 9.4 **^.^** 10^-3^ |
|  | SOSIP.664 | LB *^b^* | | | |
| **3G08** | SOSIP.v8 GT1.1 | 1.5 **^.^** 10^5^  ± 7.7 **^.^** 10^2^ | 2.9 **^.^** 10^-4^  ± 8.3 **^.^** 10^-8^ | 2.0  ± 1.1 **^.^** 10^-2^ | 2.8  ± 2.9 **^.^** 10^-3^ |
|  | SOSIP.v8 GT1.1-N276 | LB | | | |
|  | SOSIP.664 |  |  |  |  |
| **9C09** | SOSIP.v8 GT1.1 | 1.0 **^.^** 10^5^  ± 1.0 **^.^** 10^3^ | 6.2 **^.^** 10^-6^  ± 6.1 **^.^** 10^-8^ | 6.2 **^.^** 10^-2^  ± 1.2 **^.^** 10^-3^ | 3.0  ± 2.3 **^.^** 10^-2^ |
|  | SOSIP.664 | LB | | | |
| **7A03** | SOSIP.v8 GT1.1 | 5.2 **^.^** 10^5^  ± 1.6 **^.^** 10^3^ | 3.7 **^.^** 10^-6^  ± 2.2 **^.^** 10^-7^ | 7.2 **^.^** 10^-3^  ± 4.4 **^.^** 10^-4^ | 2.6  ± 1.2 **^.^** 10^-3^ |
|  | SOSIP.v8 GT1.1-N276 | 5.5 **^.^** 10^4^  ± 1.7 **^.^** 10^2^ | 2.0 **^.^** 10^-3^  ± 6.0 **^.^** 10^-6^ | 36  ± 0 | 2.7  ± 7.1 **^.^** 10^-3^ |
| **4D01** | SOSIP.v8 GT1.1 | 1.6 **^.^** 10^5^  ± 7.6 **^.^** 10^2^ | 6.0 **^.^** 10^-6^  ± 1.9 **^.^** 10^-7^ | 3.7 **^.^** 10^-2^  ± 1.0 **^.^** 10^-3^ | 2.9  ± 2.6 **^.^** 10^-2^ |

1. **Conformational-change** **model**

| **mAbs** | **HIV-1 BG505 Env trimer** | ***k_on_***  (1/Ms) | ***k_off_***  (1/s) | ***k_f_***  (1/s) | ***k_b_***  (1/s) | ***K_D_***  (nM) | ***K_F_*** | ***K_D(conf)_***  (nM) | ***S_m_*** |
| --- | --- | --- | --- | --- | --- | --- | --- | --- | --- |
| **12A01** | SOSIP.v8 GT1.1-N276 | 1.8 **^.^** 10^5^  ± 5.0 **^.^** 10^3^ *^a^* | 1.4 **^.^** 10^-2^  ± 7.5 **^.^** 10^-4^ | 5.9 **^.^** 10^-4^  ± 1.1 **^.^** 10^-4^ | 3.6 **^.^** 10^-4^  ± 1.8 **^.^** 10^-4^ | 77  ± 2.1 | 2.0  ± 0.66 | 28  ± 6.8 | 1.5  ± 1.6 **^.^** 10^-2^ |
| **9C09** | SOSIP.v8 GT1.1-N276 | 1.7 **^.^** 10^4^  ± 4.4 **^.^** 10^2^ | 3.6 **^.^** 10^-3^  ± 1.2 **^.^** 10^-4^ | 5.2 **^.^** 10^-4^  ± 5.2 **^.^** 10^-6^ | 1.0 **^.^** 10^-4^  ± 1.2 **^.^** 10^-5^ | 2.1 **^.^** 10^2^  ± 12 | 5.2  ± 0.67 | 35  ± 5.8 | 0.94  ± 1.8 **^.^** 10^-3^ |
| **A03** | SOSIP.664 | 2.4 **^.^** 10^4^  ± 2.9 **^.^** 10^2^ | 1.2 **^.^** 10^-3^  ± 3.6 **^.^** 10^-5^ | 5.9 **^.^** 10^-4^  ± 2.1 **^.^** 10^-5^ | 1.2 **^.^** 10^-4^  ± 6.9 **^.^** 10^-6^ | 50  ± 2.2 | 5.0  ± 0.28 | 8.3  ± 0.72 | 1.1  ± 2.0 **^.^** 10^-2^ |
| **4D01** | SOSIP.v8 GT1.1-N276 | 1.6 **^.^** 10^5^  ± 1.3 **^.^** 10^4^ | 1.1 **^.^** 10^-2^  ± 1.0 **^.^** 10^-4^ | 1.0 **^.^** 10^-3^  ± 3.1 **^.^** 10^-5^ | 7.2 **^.^** 10^-5^  ± 6.6 **^.^** 10^-6^ | 70  ± 6.5 | 14  ± 0.88 | 4.5  ± 0.16 | 0.39  ± 2.4 **^.^** 10^-3^ |
|  | SOSIP.664 | 1.1 **^.^** 10^5^  ± 9.1 **^.^** 10^3^ | 3.7 **^.^** 10^-3^  ± 1.0 **^.^** 10^-4^ | 1.5 **^.^** 10^-3^  ± 4.9 **^.^** 10^-5^ | 1.1 **^.^** 10^-4^  ± 9.4 **^.^** 10^-6^ | 35  ± 4.0 | 14  ± 0.75 | 2.4  ± 0.39 | 0.54  ± 9.4 **^.^** 10^-3^ |

*^a^* Tabulated values are means ± SEM of two independent replicates. Two significant digits are given for each of the mean and SEM values.

*^b^* LB: Low binding; MAb binding was detectable (> 1 RU) but too low to be modeled.

**Table S13. Validation of SPR modeling parameters.**

1. **Validation of Langmuir modeling.*^a^***

| **mAb** | **HIV-1 BG505 Env trimer** | ***T_kon_ ^b^*** | ***T_koff_*** | ***χ^2^*** (RU^2^) ***^c^*** | ***U ^d^*** |
| --- | --- | --- | --- | --- | --- |
| **12A01** | SOSIP.v8 GT1.1 | 1.8 **^.^** 10^3^  ± 14 | 2.1 **^.^** 10^2^  ± 3.0 | 3.9 **^.^** 10^-2^  ± 1.7 **^.^** 10^-4^ | 6.9  ± 0 |
| **3G08** | SOSIP.v8 GT1.1 | 3.5 **^.^** 10^2^  ± 72 | 6.1 **^.^** 10^2^  ± 1.3 **^.^** 10^2^ | 0.31  ± 0.12 | 1.5  ± 0.38 |
| **9C09** | SOSIP.v8 GT1.1 | 1.7 **^.^** 10^3^  ± 96 | 1.5 **^.^** 10^2^  ± 7.1 | 6.1 **^.^** 10^-2^  ± 7.8 **^.^** 10^-3^ | 7.9  ± 1.0 |
| **7A03** | SOSIP.v8 GT1.1 | 2.6 **^.^** 10^3^  ± 34 | 3.8 **^.^** 10^2^  ± 23 | 1.4 **^.^** 10^-2^  ± 5.2 **^.^** 10^-4^ | 4.7  ± 0.61 |
|  | SOSIP.v8 GT1.1-N276 | 5.1 **^.^** 10^2^  ± 10 | 6.6 **^.^** 10^2^  ± 10 | 0.26  ± 1.2 **^.^** 10^-2^ | 1.0  ± 0 |
| **4D01** | SOSIP.v8 GT1.1 | 1.1 **^.^** 10^3^  ± 13 | 95  ± 4.3 | 0.14  ± 1.4 **^.^** 10^-3^ | 12  ± 0 |

*^a^* Tabulated values are means ± SEM of two independent replicates. Two significant digits are given for each of the mean and SEM Only MAbs for which binding data could modeled are included in the tables.

*^b^ T* values = mean / SEM for the kinetic constant indicated by the subscripts. The higher the *T* value*,* the more significant the value of the kinetic constant. *T* > 100 indicates excellent significance.

*^c^* *χ^2^* indicates the goodness of the overall modeling; the lower the value, the better is the fit.

*^d^* *U* values indicate the uniqueness of the overall modeling. The minimum *U* value, 1, is the best; *U* < 15 is acceptable. Only Langmuir modeling yields *U* values*.*

1. **Validation of conformational-change modeling.*^a^***

| **mAb** | **HIV-1 BG505 Env trimer** | ***T_kon_*** *^b^* | ***T_koff_*** | ***T_kf_*** | ***T_kb_*** | ***χ^2^*** (RU^2^) ***^c^*** | RRB (%) ***^d^*** |
| --- | --- | --- | --- | --- | --- | --- | --- |
| **12A01** | SOSIP.v8 GT1.1-N276 | 1.1 **^.^** 10^2^  ± 1.1 | 85  ± 0.55 | 45  ± 6.6 | 17  ± 10 | 0.12  ± 6.6 **^.^** 10^-3^ | < 0 |
| **9C09** | SOSIP.v8 GT1.1-N276 | 4.0 **^.^** 10^2^  ± 10 | 4.2 **^.^** 10^2^  ± 24 | 5.0 **^.^** 10^2^  ± 17 | 1.8 **^.^** 10^2^  ± 25 | 1.5 **^.^** 10^-2^  ± 2.0 **^.^** 10^-3^ | < 0 |
| **7A03** | SOSIP.664 | 3.0 **^.^** 10^2^  ± 6.7 | 1.0 **^.^** 10^2^  ± 3.6 | 1.0 **^.^** 10^2^  ± 1.7 | 67  ± 5.2 | 9.7 **^.^** 10^-2^  ± 3.2 **^.^** 10^-3^ | 59  ± 1.6 |
| **4D01** | SOSIP.v8 GT1.1-N276 | 95  ± 7.3 | 89  ± 7.3 | 3.9 **^.^** 10^2^  ± 26 | 1.9 **^.^** 10^2^  ± 11 | 1.1 **^.^** 10^-2^  ± 1.3 **^.^** 10^-3^ | 34  ± 1.1 |
|  | SOSIP.664 | 1.4 **^.^** 10^2^  ± 8.3 | 84  ± 4.4 | 2.3 **^.^** 10^2^  ± 6.9 | 2.1 **^.^** 10^2^  ± 6.0 | 1.8 **^.^** 10^-2^  ± 3.7 **^.^** 10^-4^ | 50  ± 3.8 |

*^a^* Tabulated values are means ± SEM of two independent replicates. Two significant digits are given for each of the mean and SEM values. Only MAbs for which binding data could modeled are included in the tables.

*^b^ T* values = mean / SEM for the kinetic constant indicated by the subscripts. The higher the *T* value*,* the more significant the value of the kinetic constant. *T* > 100 indicates excellent significance.

*^c^* *χ^2^* indicates the goodness of the overall modeling; the lower the value, the better is the fit.

*^d^* Injection time variation test is reported as Relative Residual Binding (RRB (%)). Analyte was infected at varied time (30 s or 600 s) followed by dissociation for 600s. Combinations of MAb and Env that did not pass the test are not included in the table.

**Table S14. Validation of SPR modeling parameters excluding mass transfer limitation.**

1. **Excluding mass transfer limitation in Langmuir modeling** *^a^*

| **mAb** | **HIV-1 BG505 Env trimer** | ***t_c_ ^b^***  (RU ^.^ M^-1^s^-2/3^m^-1^) | ***T_tc_ ^b^*** | ***t_c_* */ k_on_ ^b^***  (RU ^.^ s^1/3^m^-1^) |
| --- | --- | --- | --- | --- |
| **12A01** | SOSIP.v8 GT1.1 | 1.2 **^.^** 10^21^  ± 1.2 **^.^** 10^21^ | 0.14  ± 0.14 | 1.6 **^.^** 10^15^  ± 1.6 **^.^** 10^15^ |
| **3G08** | SOSIP.v8 GT1.1 | 7.3 **^.^** 10^6^  ± 4.4 **^.^** 10^5^ | 32  ± 4.5 | 50  ± 3.2 |
| **9C09** | SOSIP.v8 GT1.1 | 3.3 **^.^** 10^21^  ± 3.3 **^.^** 10^21^ | 3.9 **^.^** 10^-2^  ± 3.9 **^.^** 10^-2^ | 3.2 **^.^** 10^16^  ± 3.2 **^.^** 10^16^ |
| **7A03** | SOSIP.v8 GT1.1 | 1.0 **^.^** 10^21^  ± 1.0 **^.^** 10^21^ | 1.9 **^.^** 10^-3^  ± 1.9 **^.^** 10^-3^ | 2.0 **^.^** 10^15^  ± 2.0 **^.^** 10^15^ |
|  | SOSIP.v8 GT1.1-N276 | 1.7 **^.^** 10^19^  ± 1.2 **^.^** 10^19^ | 3.8 **^.^** 10^-4^  ± 6.0 **^.^** 10^-5^ | 3.0 **^.^** 10^14^  ± 2.1 **^.^** 10^14^ |
| **4D01** | SOSIP.v8 GT1.1 | 5.9 **^.^** 10^22^  ± 3.6 **^.^** 10^22^ | 4.6 **^.^** 10^-4^  ± 2.4 **^.^** 10^-5^ | 3.7 **^.^** 10^17^  ± 2.2 **^.^** 10^17^ |

*^a^* Tabulated values are means ± SEM of two independent replicates. Two significant digits are given for each of the mean and SEM values. Only MAbs for which binding data could modeled are included in the tables.

*^b^* The flow rate-independent mass-transfer constant, ***t_c_*** , should be ≥ 10^8^ and its *T* <10 to indicate absence of mass-transfer limitation. Failing that, *t_c_* */ k_on_* should be > 10 for passable and > 100 for excellent evidence against mass-transfer limitation, in favor of kinetic limitation of the data.

1. **Excluding mass transfer limitation in conformational-change modeling** *^a^*

| **mAb** | **HIV-1 BG505 Env trimer** | ***t_c_ ^b^***  (RU.M^-1^s^-2/3^m^-1^) | ***T_tc_ ^b^*** | ***t_c_* */ k_on_ ^b^***  (RU ^.^ s^1/3^m^-1^) |
| --- | --- | --- | --- | --- |
| **12A01** | SOSIP.v8 GT1.1-N276 | 9.6 **^.^** 10^7^  ± 3.3 **^.^** 10^5^ | 0.47  ± 2.0 **^.^** 10^-2^ | 5.4 **^.^** 10^2^  ± 17 |
| **9C09** | SOSIP.v8 GT1.1-N276 | 3.2 **^.^** 10^13^  ± 4.1 **^.^** 10^12^ | 3.8 **^.^** 10^-2^  ± 3.6 **^.^** 10^-2^ | 1.9 **^.^** 10^9^  ± 2.9 **^.^** 10^8^ |
| **7A03** | SOSIP.664 | 1.3 **^.^** 10^19^  ± 1.3 **^.^** 10^19^ | 3.7 **^.^** 10^-2^  ± 3.7 **^.^** 10^-2^ | 5.7 **^.^** 10^14^  ± 5.7 **^.^** 10^14^ |
| **4D01** | SOSIP.v8 GT1.1-N276 | 2.4 **^.^** 10^7^  ± 1.1 **^.^** 10^7^ | 0.39  ± 0.17 | 1.6 **^.^** 10^2^  ± 82 |
|  | SOSIP.664 | 8.4 **^.^** 10^11^  ± 6.5 **^.^** 10^11^ | 3.9  ± 3.8 | 8.6 **^.^** 10^6^  ± 6.9 **^.^** 10^6^ |

*^a^* Tabulated values are means ± SEM of two independent replicates. Two significant digits are given for each of the mean and SEM values. Only MAbs for which binding data could modeled are included in the tables.

*^b^* The flow rate-independent mass-transfer constant, ***t_c_*** , should be ≥ 10^8^ and its *T* <10 to indicate absence of mass-transfer limitation. Failing that, *t_c_* */ k_on_* should be > 10 for passable and > 100 for excellent evidence against mass-transfer limitation, in favor of kinetic limitation of the data.

**Table S15. Cryo-EM data collection, refinement and validation statistics.**

|  | 3G08 + GT1.1 + RM20A3  (EMD-48286)  (PDB 9MIA) | 12A01 + GT1.1 + RM20A3  (EMD-48283) (PDB 9MI0) | 9C09 + GT1.1 + RM20A3  (EMD-48287) (PDB 9MIB) | 7A03 + BG505 + RM20A3  (EMD-48291) (PDB 9MII) | 4D01 + BG505 + RM20A3  (EMD-48290) (PDB 9MIH) |
| --- | --- | --- | --- | --- | --- |
| **Data collection and processing** |  |  |  |  |  |
| Microscope | TFS Glacios | TFS Glacios | TFS Glacios | TFS Glacios | TFS Glacios |
| Voltage (keV) | 200 | 200 | 200 | 200 | 200 |
| Camera | TFS Falcon 4i | TFS Falcon 4i | TFS Falcon 4i | TFS Falcon 4i | TFS Falcon 4i |
| Collection mode | Counting | Counting | Counting | Counting | Counting |
| Magnification | 190,000x | 190,000x | 190,000x | 190,000x | 190,000x |
| Pixel size at detector (Å) | 0.718 | 0.718 | 0.718 | 0.718 | 0.718 |
| Total electron exposure (e–/Å^2^) | 45.2 | 45.2 | 45.3 | 45.2 | 45.3 |
| Exposure rate (e-/pixel/sec) | 9.034 | 9.034 | 8.937 | 9.034 | 8.937 |
| Number of EER frames | 40 | 40 | 40 | 40 | 40 |
| Defocus range (μm) | -0.7 to -1.8 | -0.7 to -1.8 | -0.7 to -1.8 | -0.7 to -1.8 | -0.7 to -1.8 |
| Automation software | EPU | EPU | EPU | EPU | EPU |
| Micrographs collected (no.) | 5,001 | 3,867 | 4,718 | 3,185 | 6,830 |
| Micrographs used (no.) | 4,198 | 3,644 | 4,699 | 3,134 | 6,734 |
| Initial particle images (no.) | 464,530 | 399,688 | 560,365 | 371,969 | 813,100 |
| Final particle images (no.) | 99,609 | 165,097 | 133,910 | 51,005 | 10,765 |
| Symmetry | C3 | C3 | C3 | C1 | C1 |
| Map resolution (masked/unmasked Å) | 2.8/3.3 | 2.8/3.2 | 2.8/3.2 | 3.3/4.1 | 3.9/7.6 |
| FSC threshold | 0.143 | 0.143 | 0.143 | 0.143 | 0.143 |
| Map sharpening *B* factor (Å^2^) | -71.3 | -79.2 | -77.3 | -49.9 | -29.5 |
| Map pixel size (Å) | 1.034 | 1.034 | 1.034 | 1.034 | 1.034 |
| Map resolution range (Å) | 2.5-3.5 | 2.5-3.5 | 2.5-3.5 | 3.0-4.5 | 3.5-6.0 |
|  |  |  |  |  |  |
| **Refinement** |  |  |  |  |  |
| Initial model used (PDB code) | 8SW3 | 8SW3 | 8SW3 | 6X9R | 6X9R |
| Refinement package | Phenix RSR | Phenix RSR | Phenix RSR | Phenix RSR | Phenix RSR |
| Model resolution (Å) | 2.9 | 2.9 | 2.9 | 3.5 | 4.2 |
| FSC threshold | 0.5 | 0.5 | 0.5 | 0.5 | 0.5 |
| EMRinger score | 4.11 | 3.36 | 3.88 | 3.10 | 1.80 |
| CC (mask) | 0.83 | 0.82 | 0.78 | 0.82 | 0.81 |
| *Model composition* |  |  |  |  |  |
| Non-hydrogen atoms | 24,660 | 25,002 | 25,266 | 20,957 | 20,955 |
| Protein residues | 3,069 | 3,099 | 3,075 | 2,579 | 2,577 |
| Ligands | 60 | 60 | 96 | 66 | 66 |
| *Mean B factors (Å^2^)* |  |  |  |  |  |
| Protein | 38.17 | 41.13 | 44.97 | 40.68 | 122.67 |
| Ligand | 58.21 | 62.35 | 50.57 | 50.28 | 134.66 |
| *R.m.s. deviations* |  |  |  |  |  |
| Bond lengths (Å) | 0.005 | 0.005 | 0.005 | 0.006 | 0.005 |
| Bond angles (°) | 0.986 | 0.999 | 0.946 | 1.139 | 0.943 |
| *Validation* |  |  |  |  |  |
| MolProbity score | 1.55 | 1.11 | 1.14 | 1.38 | 1.73 |
| Clashscore | 5.30 | 3.17 | 2.09 | 2.91 | 5.92 |
| Poor rotamers (%) | 0.15 | 0.00 | 0.11 | 0.40 | 0.13 |
| *Ramachandran plot* |  |  |  |  |  |
| Favored (%) | 96.12 | 98.43 | 97.11 | 95.72 | 93.93 |
| Allowed (%) | 3.88 | 1.57 | 2.89 | 4.24 | 6.07 |
| Disallowed (%) | 0.00 | 0.00 | 0.00 | 0.04 | 0.00 |
| Cβ outliers (%) | 0.00 | 0.00 | 0.00 | 0.00 | 0.00 |
| CaBLAM outliers (%) | 1.45 | 1.30 | 1.32 | 2.51 | 3.81 |

| **Table S16. X-ray data collection and refinement statistics for eOD-GT8 Fab complexes** | | | | |
| --- | --- | --- | --- | --- |
| **Data Collection** | **4D01_eOD-GT8** | **3G08_eOD-GT8** | **9C09_eOD-GT8** | **12A01_eOD-GT8** |
| Crystallization Condition | 1.2M NaH_2_PO_4_, 0.1M CAPS, 0.8M K₂HPO₄, 25% glycerol, 0.2M lithium sulfate | 0.2M ammonium formate, 10% ethylene glycol, 20% PEG 3350 | 20% PEG-300, 10% glycerol, 5% PEG-8000, 0.1M Tris pH 8.5 | 1.2M NaH_2_PO_4_, 0.1M CAPS,  0.8M K₂HPO₄, 25% glycerol, 0.2M lithium sulfate |
| Wavelength (Å) | 0.92015 | 0.92019 | 0.92015 | 0.92015 |
| Resolution (Å) | 33.90 - 1.97 (2.00 - 1.97) | 34.70 - 1.74  (1.77 - 1.74) | 33.23 - 1.93  (1.96 - 1.93) | 33.90 - 2.20 (2.24 - 2.20) |
| Space group | P 2_1_ 2_1_ 2_1_ | C 1 2 1 | P 2_1_ 2_1_ 2_1_ | P 2_1_ 2_1_ 2_1_ |
| Unit cell a, b, c (Å) | 53.4 71.2 219.3 | 141.1 44.4 103.9 | 53.0 74.4 208.1 | 53.6 72.3 218.6 |
| α, β, γ (°) | 90 90 90 | 90 119.9 90 | 90 90 90 | 90 90 90 |
| Total reflections | 779,877 | 407,069 | 859,399 | 608,728 |
| Unique reflections | 60,382 | 58,145 | 62,686 | 44,221 |
| Multiplicity | 12.9 (12.9) | 7.0 (7.2) | 13.7 (13.9) | 13.8 (14.1) |
| Completeness (%) | 99.9 (99.6) | 99.9 (99.6) | 99.4 (93.5) | 99.9 (99.8) |
| Mean I/sigma(I) | 5.8 (1.0) | 6.8 (1.0) | 10.6 (0.6) | 10.6 (2.4) |
| Rsym | 0.37 (>1) | 0.17 (>1) | 0.16 (>1) | 0.19 (>1) |
| Rpim | 0.11 (1.5) | 0.07 (0.68) | 0.04 (1.49) | 0.05 (0.37) |
| CC1/2 | 0.99 (0.39) | 0.99 (0.35) | 0.99 (0.32) | 0.99 (0.80) |
| **Refinement Statistics** | | | | |
| Resolution (Å) | 33.90 - 1.97 | 34.70 - 1.74 | 33.23 - 1.93 | 33.90 - 2.20 |
| Reflections total / Rfree | 60,352 / 2987 | 58,131 / 2884 | 62,338 / 3096 | 44,147 / 1998 |
| Rcryst / Rfreee | 0.22 / 0.25 | 0.21 / 0.25 | 0.23 / 0.27 | 0.22 / 0.25 |
| No. of copies in ASU | 1 | 1 | 1 | 1 |
| Number of atoms | 4740 | 4632 | 4750 | 4728 |
| macromolecules | 4542 | 4379 | 4596 | 4538 |
| ligands | 28 | 28 | 28 | 28 |
| solvent | 170 | 225 | 126 | 162 |
| Average B-factor (Å^2^) | 31 | 26 | 44 | 34 |
| macromolecules | 31 | 26 | 44 | 34 |
| solvent | 31 | 28 | 39 | 34 |
| Wilson B-factor (Å^2^) | 28 | 20 | 36 | 31 |
| **RMSD from ideal geometry** | | | | |
| Bond angle (^o^) | 1.35 | 0.77 | 1.13 | 0.53 |
| Bond length (Å) | 0.021 | 0.005 | 0.01 | 0.002 |
| **Ramachandran statistics (%)** | | | | |
| Favored | 96.1 | 97.3 | 95.5 | 97.2 |
| Allowed | 3.9 | 2.7 | 4.5 | 2.8 |
| Outliers | 0 | 0 | 0 | 0 |
| **PDB Code** | **9MIC** | **9MID** | **9MIF** | **9MJ3** |
| Statistics for the highest-resolution shell are shown in parentheses. | | | | |

| **Table S17.  X-ray data collection and refinement statistics for unliganded Fabs** | | | | | | | | | |
| --- | --- | --- | --- | --- | --- | --- | --- | --- | --- |
| **Data Collection** | **4D01** | **3G08** | | | **9C09** | | **7A03** | | **12A01** |
| Crystallization Condition | 20% PEG-8000, 0.2M NaCl, 0.1M phosphate-citrate, pH 4.2 | 1M Na/K tartrate, 0.2M LiSO_4_, 0.1M CHES, pH 9.5 | | | 20% PEG 3350,  0.2M K-fluoride, pH 7.2 | | 20% PEG-1000, 0.2M LiSO_4_,  0.1M phosphate-citrate, pH 4.2 | | 50% PEG-200, 0.2M MgCl_2_, 0.1M cacodylate, pH 6.5 |
| Wavelength (Å) | 0.92015 | 0.92026 | | | 0.92015 | | 0.92026 | | 0.92015 |
| Resolution (Å) | 33.47 - 2.70  (2.75 - 2.70) | 50.00 - 3.49 (3.56 - 3.49) | | | 34.78 - 2.40  (2.44 - 2.40) | | 34.47 - 3.06  (3.11 - 3.06) | | 30.00 - 3.00  (3.05 - 3.00) |
| Space group | P 2_1_ 2_1_ 2_1_ | P 3_2_ | | | P 2_1_ 2_1_ 2_1_ | | P 1 2_1_ 1 | | P 4_3_ |
| Unit cell a, b, c (Å,) | 73.0 89.9 165.8 | 107.9 107.9 223.5 | | | 70.8 82.3 162.9 | | 50.4 137.8 62.9 | | 73.2 73.2 274.4 |
| α, β, γ (°) | 90 90 90 | 90 90 120 | | | 90 90 90 | | 90 108.3 90 | | 90 90 90 |
| Total reflections | 416,515 | | 350,694 | 431,056 | | 105,282 | | 386,509 | |
| Unique reflections | 30,747 (1498) | 36,692 (1811) | | | 38,042 (1887) | | 15,508 (3080) | | 28,654 (1477) |
| Multiplicity | 13.5 (14.1) | 9.6 (6.7) | | | 11.3 (11.8) | | 6.8 (7.0) | | 13.5 (12.5) |
| Completeness (%) | 100.0 (99.8) | 99.7 (98.3) | | | 100.0 (100.0) | | 99.8 (99.4) | | 99.9 (100.0) |
| Mean I/sigma(I) | 8.5 (1.7) | 2.8 (0.4) | | | 8.2 (2.5) | | 3.37 (1.2) | | 19.5 (0.9) |
| Rsym | 0.22 (1.85) | 0.36 (1.90) | | | 0.28 (1.09) | | 0.53 (1.78) | | 0.11 (1.69) |
| Rpim | 0.06 (0.51) | 0.12 (0.76) | | | 0.09 (0.33) | | 0.22 (0.72) | | 0.03 (0.49) |
| CC1/2 | 0.99 (0.81) | 0.99 (0.46) | | | 0.98 (0.78) | | 0.91 (0.43) | | 0.99 (0.56) |
| **Refinement Statistics** | | | | | | | | | |
| Resolution (Å) | 33.47 - 2.70 | 39.59 - 3.49 | | | 34.78 - 2.40 | | 34.47 - 3.06 | | 29.55 - 3.00 |
| Reflections total / Rfree | 30,676 / 1996 | 34,760 / 1962 | | | 37,670 / 1976 | | 15,483 / 744 | | 28,260 / 2019 |
| Rcryst / Rfreee | 0.24 / 0.28 | 0.30 / 0.34 | | | 0.23 / 0.28 | | 0.26 / 0.32 | | 0.24 / 0.29 |
| No. of copies in ASU | 2 | 4 | | | 2 | | 2 | | 2 |
| Number of atoms | 6490 | 12328 | | | 6730 | | 6199 | | 6618 |
| macromolecules | 6458 | 12328 | | | 6548 | | 6199 | | 6618 |
| ligands | 0 | 0 | | | 0 | | 0 | | 0 |
| solvent | 32 | 0 | | | 182 | | 0 | | 0 |
| Average B-factor (Å^2^) | 44 | 139 | | | 21 | | 44 | | 111 |
| macromolecules | 44 | 139 | | | 21 | | 44 | | 111 |
| solvent | 34 | - | | | 19 | | - | | - |
| Wilson B-factor (Å^2^) | 44 | 103 | | | 20 | | 44 | | 99 |
| **RMSD from ideal geometry** | | | | | | | | | |
| Bond angle (^o^) | 0.5 | 0.4 | | | 0.5 | | 0.49 | | 0.44 |
| Bond length (Å) | 0.002 | 0.001 | | | 0.002 | | 0.002 | | 0.001 |
| Ramachandran statistics (%) | | | | | | | | | |
| Favored | 96.2 | 97.3 | | | 97.5 | | 95.2 | | 96.9 |
| Allowed | 3.8 | 2.7 | | | 2.5 | | 4.8 | | 3.1 |
| Outliers | 0 | 0 | | | 0 | | 0 | | 0 |
| **PDB Code** | **9MJC** | **9MJD** | | | **9MJI** | | **9MJ6** | | **9MK4** |
| Statistics for the highest-resolution shell are shown in parentheses. | | | | | | | | | |
